# Fully synthetic platform to rapidly generate tetravalent bispecific nanobody–based immunoglobulins

**DOI:** 10.1073/pnas.2216612120

**Published:** 2023-06-05

**Authors:** Laetitia Misson Mindrebo, Hejun Liu, Gabriel Ozorowski, Quoc Tran, Jordan Woehl, Irene Khalek, Jessica M. Smith, Shawn Barman, Fangzhu Zhao, Celina Keating, Oliver Limbo, Megan Verma, Jingjia Liu, Robyn L. Stanfield, Xueyong Zhu, Hannah L. Turner, Devin Sok, Po-Ssu Huang, Dennis R. Burton, Andrew B. Ward, Ian A. Wilson, Joseph G. Jardine

**Affiliations:** ^a^International AIDS Vaccine Initiative Neutralizing Antibody Center, The Scripps Research Institute, La Jolla, CA 92037; ^b^International AIDS Vaccine Initiative, New York, NY 10004; ^c^Department of Integrative Structural and Computational Biology, The Scripps Research Institute, La Jolla, CA 92037; ^d^Consortium for HIV/AIDS Vaccine Development, The Scripps Research Institute, La Jolla, CA 92037; ^e^Department of Immunology and Microbiology, The Scripps Research Institute, La Jolla, CA 92037; ^f^Department of Bioengineering, Stanford University, Stanford, CA 94305; ^g^Ragon Institute of Massachusetts General Hospital, Massachusetts Institute of Technology, and Harvard University, Cambridge, MA 02139; ^h^Skaggs Institute for Chemical Biology, The Scripps Research Institute, La Jolla, CA 92037

**Keywords:** synthetic library, bispecific antibody, nanobody, SARS-CoV-2 neutralization

## Abstract

Nanobodies bind a target antigen with a kinetic profile similar to a conventional antibody, but exist as a single heavy chain domain that can be readily multimerized to engage antigen via multiple interactions. Presently, most nanobodies are produced by immunizing camelids; however, platforms for animal-free production are growing in popularity. Here, we describe the development of a fully synthetic nanobody library based on an engineered human V_H_3-23 variable gene and a multispecific antibody-like format designed for biparatopic target engagement. To validate our library, we selected nanobodies against the SARS-CoV-2 receptor–binding domain and employed an on-yeast epitope binning strategy to rapidly map the specificities of the selected nanobodies. We then generated antibody-like molecules by replacing the V_H_ and V_L_ domains of a conventional antibody with two different nanobodies, designed as a molecular clamp to engage the receptor-binding domain biparatopically. The resulting bispecific tetra-nanobody immunoglobulins neutralized diverse SARS-CoV-2 variants with potencies similar to antibodies isolated from convalescent donors. Subsequent biochemical analyses confirmed the accuracy of the on-yeast epitope binning and structures of both individual nanobodies, and a tetra-nanobody immunoglobulin revealed that the intended mode of interaction had been achieved. This overall workflow is applicable to nearly any protein target and provides a blueprint for a modular workflow for the development of multispecific molecules.

## Significance

Nanobodies are a promising class of biologics that can be used to prevent or treat viral infections. Here, we describe the production and validation of a discovery library that produces single-domain nanobodies using an engineered human antibody variable gene segment. As a test case, anti-SARS-CoV-2 nanobodies were isolated from this library and pairs of complementary nanobodies were incorporated into an antibody-like molecule that targets the receptor-binding domain using a biparatopic mode of engagement. This modular bispecific format enabled the rapid testing of nanobody pairs, and we show that incorporating pairs of nanobodies with different specificities can have synergistic effects on neutralization breadth and potency.

The use of biologics for the prevention or treatment of viral disease is increasing. Palivizumab was the first monoclonal antibody approved for the prevention of respiratory syncytial virus (RSV) disease in infants, but Ebola virus outbreaks and the COVID-19 pandemic have led to the development of several other monoclonal antibodies. Antibodies have the potential to be transformative within this space due to their high specificity, long half-life, and ability to coordinate the response from the innate immune system via Fc-mediated interactions. One of the main challenges in using antibodies for antiviral indications is the ever-present risk of resistant viral variants. This was exemplified by the evolution of severe acute respiratory syndrome coronavirus 2 (SARS-CoV-2) variants of concern (VOCs) during the COVID-19 pandemic that were partially or fully resistant to the therapeutic antibody countermeasures (1). Strategies to counter this viral escape generally focus on targeting functionally conserved epitopes and/or the use of a cocktail of multiple antibodies that recognize nonoverlapping epitopes, thus requiring multiple mutations for the virus to effectively evade all antibodies in the cocktail (2). Single-domain “nanobodies” are promising as antiviral biologics because they recognize their targets using a single-variable heavy (V_HH_) domain. One advantage of this smaller size is that nanobodies can target epitopes that are sterically inaccessible to bulkier antibodies that require both variable heavy (V_H_) and light (V_L_) domains to form the antibody paratope (3). The lack of light chain pairing greatly simplifies the expression of multiple nanobodies targeting different specificities as a single molecule. The most common method to obtain nanobodies requires immunizing camelids (llamas or alpacas) that produce V_HH_s as part of their Ig repertoire, but phage- and yeast-based synthetic platforms can also be used for de novo discovery. To date, neutralizing nanobodies have been developed for numerous viruses including HIV (4, 5), influenza (6), RSV (7, 8), SARS-CoV-2 (9, 10), hepatitis C (11), and rabies virus (12). In most instances, monomeric V_HH_ exhibits moderate neutralizing potency, but when homo- or hetero-nanobodies are placed in tandem, avid interactions greatly increase the overall neutralization potency (9, 13, 14). While the value of combining multiple nanobodies into a single molecule has been clearly demonstrated (6, 9, 10, 13, 15), selecting the candidate nanobodies to include generally requires either a priori structural information, or the screening of many constructs to find synergistic nanobody combinations.

Because nanobodies from other species are often immunogenic in humans, we created a human-based nanobody library and validated this library using SARS-CoV-2 as a test antigen. We developed an on-yeast competitive selection strategy paired with deep sequencing to rapidly determine the specificity of the selected nanobodies. The epitope binning information was then used to select candidate nanobodies targeting nonoverlapping epitopes that can be incorporated into a chimeric nanobody/antibody hybrid architecture by replacing the conventional antibody V_H_ and V_L_ domains with different nanobodies. This modular format facilitates bispecific nanobody–based creation through the native Ig C_H_1/C_L_ domain pairing and allows for the rapid production of different nanobody combinations by mixing different plasmids during the transfection. The resulting bispecific tetra-nanobody immunoglobulin (bsNb_4_-Ig) can achieve multivalent interactions through the two nanobodies on a single fragment antigen-binding region (Fab) and/or through the contribution of nanobodies on adjacent Fab arms of the molecule. Furthermore, nanobodies that recognize nonoverlapping epitopes may facilitate biparatopic engagement of a single target molecule, provided the linkers that connect the nanobodies to the constant domain are sufficiently long. The presence of the Fc domain also enables the use of conventional purification strategies during production and provides the benefit of Fc interactions in vivo, including long serum half-life and antibody effector functions. Lastly, we structurally characterized the selected nanobodies to confirm the mode of interaction compared to conventional antibodies.

## Results

### hV_HH_323 Construction and Validation.

**Fig. 1.**
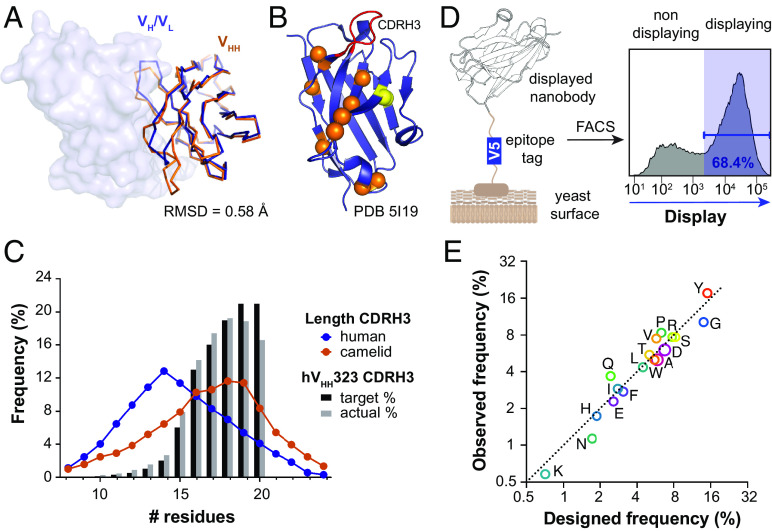
Properties of the synthetic human-based hV_HH_323 library. (*A*) Overlay of the V_H_ domain from a conventional human IgG (PDB 5I19, blue) with a representative camel V_HH_ (PDB 5U65, orange). Structures were aligned on the V_HH_, and the rmsd of the alpha carbons is indicated. The V_L_ domain is shown as surface. (*B*) V_H_ domain from a human antibody that utilizes V_H_3-23 (PDB 5I19) with the positions of the mutations that were introduced to produce the hV_HH_323 scaffold highlighted as spheres. Orange spheres are “camelizing” mutations to remove the need for LC pairing. Yellow spheres indicate the additional disulfide bond that was introduced to increase stability. The variable CDRH3 is colored in red. (*C*) CDRH3 loop length distribution from the transformed hV_HH_323 library obtained by deep sequencing analysis compared to human (conventional antibodies) (22) and camelids (HC-only antibodies) (23) CDRH3 repertoires. (*D*) Schematic yeast display of the hV_HH_323 nanobodies with a V5 epitope tag. (*E*) Deep sequencing analysis of AA frequencies of diversified positions in CDRH3s, showing both the intended frequencies and those observed in the transformed library.

The human-based nanobody library was designed to have a conserved variable domain (including CDRH1 and CDRH2) with all the diversity localized in the CDRH3 loop, mimicking the diversification of a naïve immune repertoire. Based on the high sequence identity between camelids V_HH_s and human V_H_3s (16) (Fig. 1*A*), a synthetic V_HH_ scaffold was developed by incorporating common V_HH_ mutations into the human V_H_3-23 heavy chain (HC) gene (Fig. 1*B*). Our rationale for starting with the human V_H_ gene and adding “camelizing” mutations was that it would be more human than a camelid V_HH_, thus contains fewer nonself epitopes, potentially reducing the likelihood of antidrug antibody development. We looked at predicted class II binding during the design of the hV_HH_323 base, but rational deimmunization is poorly understood for humans and we decided to instead maximize the similarity of our starting scaffold with human V_H_ genes. T cells should be tolerized against those peptides, thus minimizing potential epitopes. The starting scaffold (hV_H_323) was modified by including previously reported “camelizing” mutations that remove the need for light chain (LC) pairing (17–20), and mutations from consensus V_HH_ sequences that removed surface-exposed hydrophobic residues (*SI Appendix*, Fig. S1). To further stabilize the V_HH_ scaffold, an additional internal disulfide bond was introduced by mutating Ser49 and Ile69 to Cys (21).

The CDRH3 loops all begin and end with the CAR and FDYW motifs, respectively. Between these conserved amino acids (AA), 5 to 15 randomized AAs were inserted, resulting in nanobodies with CDRH3 loop lengths of 10 to 20 AAs. The AA frequencies in the CDRH3 loops were designed based on the nontemplated AA frequencies found in naturally occurring antibodies and nanobodies, excluding Met and Cys to avoid producing nanobodies with unwanted chemical liabilities (*SI Appendix*, *Text*). CDRH3 lengths were distributed following a sigmoidal distribution rather than mimicking the naturally occurring normal distribution (Fig. 1*C*). The right-shifted distribution was selected because the theoretical diversity of the shorter CDRH3 loops is relatively small and would be oversampled if a normal distribution were used (*SI Appendix*, Fig. S2). We also hypothesized that longer CDRH3 loops could help better access recessed epitopes. DNA encoding the human-based nanobody library was transformed into a yeast surface display vector via homologous recombination with an estimated diversity of 3 × 10^9^, based on a colony formation assay performed after the transformation (*SI Appendix*, *Text*). The nanobodies displayed well on the yeast surface, with ~67% of the library showing surface display and a median 180-fold increase in signal between the displaying and nondisplaying cell populations (Fig. 1*D*). Deep sequence analysis of nanobody-encoding DNA recovered from the transformed cells showed a good agreement with the target library specifications for both CDRH3 length distributions and AA frequencies (Fig. 1 *C* and *E*).

### Discovery of Binders Using SARS-CoV-2 Receptor–Binding Domain (RBD).

**Fig. 2.**
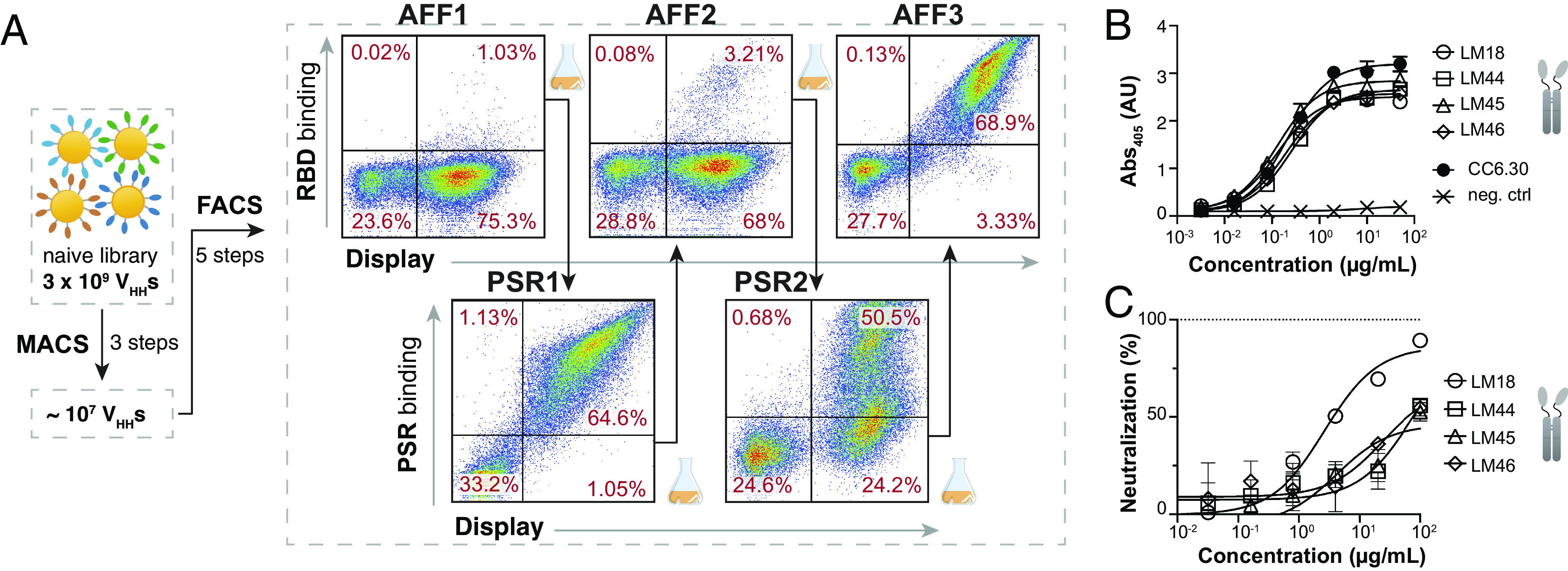
Selection of SARS-CoV-2 RBD–specific nanobodies. (*A*) FACS workflow to enrich populations in antigen-specific clones. Cells in AFF1, AFF2, and AFF3 are labeled with 100 nM biotinylated RBD to depict the enrichment overtime. Cells in PSR1 and PSR2 are labeled with biotinylated PSR preparation. The RBD and PSR concentrations used for sorting are indicated in *SI Appendix*, *Text*. (*B*) ELISA plot showing Fc-fused LM18, LM44, LM45, and LM46 binding to SARS-CoV-2 RBD. CC6.30 was used as a positive control and a nanobody-Fc from our library not selected for RBD binding was used as a negative control. The assay was run in duplicate. (*C*) Neutralization assay of Wuhan-1 SARS-CoV-2 PSV for the four nanobodies tested showing neutralization by LM18. Assay was run in triplicate. Error bars indicate the SD of the mean.

Upon completion of the library, we next set out to validate it using SARS-CoV-2 RBD as a test antigen. The overall library screening strategy was performed using a combination of magnetic-activated cell sorting (MACS) followed by fluorescence-activated cell sorting (FACS) (Fig. 2*A*). After each round of selection, the collected cell population was expanded and reinduced prior to the next round. The naïve library was too large to be screened using conventional FACS, so three rounds of MACS were used to bulk enrich the small number of SARS-CoV-2-reactive clones. RBD-reactive cells were enriched in the first round of MACS by labeling 2 × 10^11^ cells from the induced library with biotinylated SARS-CoV-2 RBD. Excess antigen was removed, and the cells were then incubated with streptavidin-conjugated magnetic microbeads. Cells bound to the magnetic microbeads were magnetically enriched, expanded, and reinduced for additional rounds of selection. In this first positive MACS selection, the collected cells were enriched for SARS-CoV-2 RBD–reactive clones, but also were enriched for streptavidin- and microbead-reactive clones. All selected cells were expanded and reinduced for additional rounds of selections. To deplete the latter two populations, the second round of MACS was a counterselection, where induced cells were incubated with the streptavidin-conjugated magnetic microbeads only and the nonreactive population was collected. Immediately following the counterselection and without expanding the collected cells, a third MACS selection was used to further enrich the RBD-reactive clones.

After MACS enrichments, five additional rounds of multicolor FACS were used to isolate clones with high binding affinity and specificity for RBD. In FACS rounds 1, 3, and 5 (AFF1, 2, and 3), cells were labeled with subsaturating concentrations of biotinylated RBD, followed by fluorescently conjugated streptavidin and an anti-V5 antibody to quantify the amount of RBD and the amount of surface-displayed nanobody, respectively. Normalizing for the level of nanobody displayed on the surface of the cell, the high RBD-binding clones were enriched. To help ensure the selected nanobodies were specific for RBD, negative selections were interspersed at FACS round 2 and 4 (PSR1 and 2) to remove polyreactive “sticky” clones (24). In this counterselection, cells were labeled with a complex preparation of biotinylated and detergent-solubilized HEK cell membrane proteins (polyspecificity reagent or PSR) and the PSR-low fraction was collected, again normalizing for the level of nanobody display on the cell surface (Fig. 2*A*).

As this was the first antigen screened against this library, we sought to quickly test the specificity and biochemical behavior of the selected nanobodies before conducting a more in-depth analysis of the enriched population. Sanger sequencing of 96 AFF3 clones found the library to be highly enriched, recovering a total of four unique clones. These four nanobodies were expressed in Expi293 cells as both the nanobody alone (His-tagged) and linked to a human Fc to create a homodimeric nanobody fusion protein. Nanobodies expressed well in both formats, producing a normalized expression titer of 50 to 200 mg/L and 220 to 450 mg/L for the His-tagged nanobodies and Fc fusions, respectively. Both formats were monodispersed by analytical size-exclusion chromatography (SEC). Enzyme-linked immunosorbent assay (ELISA) confirmed RBD binding of the four variants (Fig. 2*B*), and no polyspecific binding was observed in our standard assays (*SI Appendix*, Fig. S3). Finally, we tested the four nanobody–Fc fusions in a SARS-CoV-2 pseudovirus (PSV) neutralization assay and found that one of the nanobodies, LM18, neutralized with an IC_50_ of 2.65 µg/mL (66 nM) (Fig. 2*C* and *SI Appendix*, Table S1). Interestingly, LM18 also neutralized SARS-CoV-1 PSV with an IC_50_ of 0.5 µg/mL (or 12 nM) (*SI Appendix*, Fig. S4) as it serendipitously targeted a conserved epitope between the two variants.

### FACS-Based Epitope Binning and Deep Sequencing to Determine Library Specificities.

**Fig. 3.**
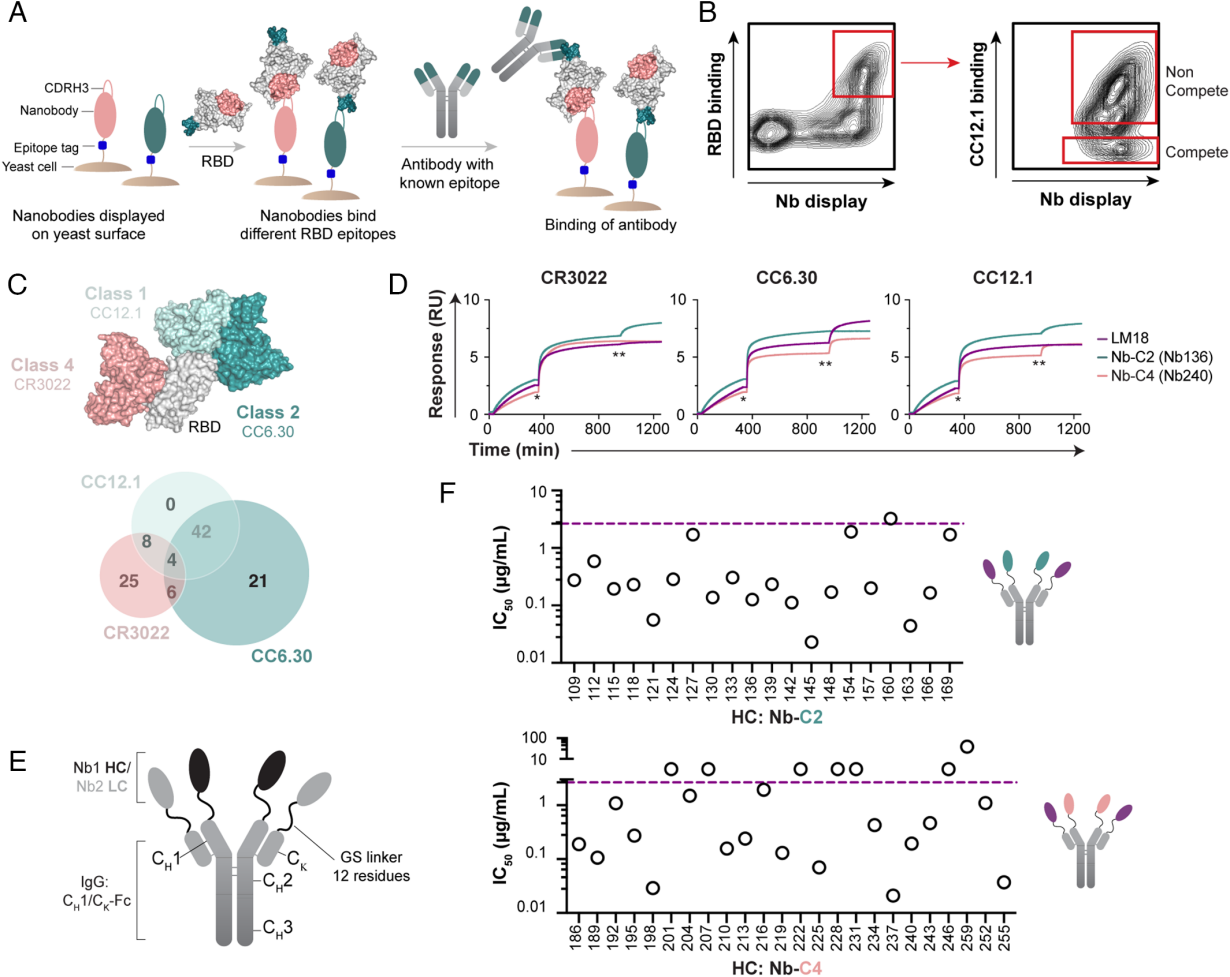
Design of bispecific antibodies and neutralization of Wuhan-1 SARS-CoV-2 PSV. (*A*) FACS-based epitope binning on yeast cell surface. RBD was first added to the induced yeast cells and binds to the nanobodies displayed on the surface. Then, the competitive antibody (CR3022, CC12.1, or CC6.30) was incubated and binds RBD only if the nanobody does not bind a similar epitope, or approaches RBD at an angle that is not blocked by the nanobody. (*B*) FACS plot showing the selection of competitive (C) and noncompetitive (NC) RBD-specific clones with CC12.1. (*C*) Nanobody binning based on C/NC profile and NGS analysis. Graphical representation of the three antibodies bound to RBD to show the location of their respective epitopes and Venn diagram with counts of competing nanobodies.  (*D*) Confirmation of nanobody epitope by BLI-based assay. One representative for each of the class 2 (Nb-C2-136) and class 4 (Nb-C4-240) nanobodies is displayed. RBD was first added to the BLI sensor. Then, the competitive antibody (CR3022, CC12.1, or CC6.30) was then incubated (*) until signal saturation, followed by the tested nanobodies from our library (**). (*E*) Design of the bsNb_4_-Igs with one nanobody linked to C_H_1 and another nanobody linked to C_L_ (*kappa* chain C_K_). (*F*) IC_50_ values (µg/mL) of bsNb_4_-Igs (Nb-C2 HC/LM18 LC, *Top* and Nb-C4 HC/ LM18 LC, *Bottom*) tested for neutralization of Wuhan-1 SARS-CoV-2 PSV. The IC_50_ value of LM18-Fc (2.65 µg/mL) is indicated by the purple dotted line.

After confirming that the base hV_HH_323 could produce biochemically well-behaved nanobodies that bound a target antigen with high specificity, we next set out to determine how many nanobodies were enriched during the selections and what epitopes on RBD were targeted by these nanobodies. Epitope specificity was determined using an on-yeast competition assay with three structurally characterized SARS-CoV-2 antibodies: CC12.1 (class 1), CC6.30 (class 2), and CR3022 (class 4) (Fig. 3 *A*–*C*) (25–27). Cells from the outgrowth following the FACS 4-negative selection (PSR2) were analyzed, as a large fraction of clones in this population were RBD reactive and had undergone both polyreactive negative selections, and thus should have relatively few sticky clones. The induced library was labeled with SARS-CoV-2 RBD, and then incubated with one of the three anti-SARS-CoV-2 antibodies. Fluorescent secondaries were used to measure the amount of nanobody on the surface of the cell, the amount of RBD bound, and the amount of the SARS-CoV-2 antibody bound. In this format, nanobodies that recognized overlapping epitopes with the human IgGs would block the antibody from binding. For analysis, both the competitive (C) population (nanobody competes with the human antibody for binding) and the noncompetitive (NC) population (both the nanobody and antibody engage RBD simultaneously) were sorted. The starting population and competition sorts for CC12.1, CC6.30, and CR3022 were deep sequenced to analyze the nanobodies in each population. After filtering to remove likely sequencing artifacts, 123 unique nanobodies were observed in the datasets (*SI Appendix*, Table S2). Epitope bins were assigned by defining an overlapping epitope with a tested SARS-CoV-2 antibody as having a C/NC ratio >10, and C/NC ratio <10 for a nonoverlapping epitope (*SI Appendix*, *Text*). From the 123 unique nanobodies identified, 100 nanobodies have a clear competitive binding profile with at least one of the three tested antibodies (Fig. 3*C*), whereas 13 nanobodies were identified as NC with all the three antibodies, indicating they likely bind elsewhere on the RBD. Four nanobodies seemed to compete with all the three antibodies, and six others seemed to compete with both CR3022 and CC6.30. Structurally, the CR3022 and CC6.30 epitopes are situated so that one nanobody should not be able to simultaneously overlap with both (Fig. 3*C*), but it is possible that nanobody binding induces a conformational change in RBD that prevents the antibodies from simultaneous binding. Ultimately, the epitopes targeted by these 10 nanobodies remain ambiguous.

A total of 45 nanobodies were selected for expression and validation to further examine the biochemical behavior of nanobodies from this library, as well as to confirm the accuracy of the on-yeast epitope binning. Twenty-one nanobodies binned to the Class 2, CC6.30-compete epitope (referred to as Nb-C2) and 24 nanobodies to the Class 4, CR3022-compete epitope (referred to as Nb-C4). Nanobodies were expressed as Fc fusion constructs, their size distribution was checked by SEC (*SI Appendix*, Fig. S5), and specific binding to SARS-CoV-2 RBD was confirmed by ELISA (*SI Appendix*, Figs. S6 and S7). To evaluate the accuracy of our FACS-based epitope binning, we performed biolayer interferometry (BLI)–based competition assays with the three antibodies used for the selection (CR3022, CC12.1, or CC6.30). The results are consistent with the on-yeast binning and validate the approach used to select our nanobodies (Fig. 3*D* and *SI Appendix*, Fig. S8).

### Using Epitope Information to Develop Bispecific Constructs.

To improve the functional properties of the selected nanobodies, we sought strategies to format several nanobodies into a single molecule, aided by the epitope binning data. It has been shown that nanobodies can be readily assembled into multimers using peptide linkers, which can result in improved binding, and consequently, neutralization (6, 9, 10, 13, 15). Multimerizing a single nanobody can improve neutralization by facilitating interspike and/or intraspike cross-linking (9), and incorporating nanobodies targeting nonoverlapping epitopes has the potential added benefits of facilitating biparatopic interactions with a single subunit and consequently greater resistance to neutralization escape (6, 28). Our approach to combine multiple nanobodies was to construct a bispecific tetra-nanobody-based Ig (bsNb_4_-Ig) by replacing the antibody V_H_ and V_L_ domains with two nanobodies that target distinct RBD epitopes (Fig. 3*E*) (29–33). Nanobodies were linked to the C_H_1 and C_L_ (*kappa* chain C_K_) domains by a flexible Gly/Ser linker, ensuring a 2:2 ratio in the bsNb_4_-Ig that could be easily expressed and purified using standard antibody production protocols. This linkage potentially facilitated simultaneous nanobody engagement on a single RBD, on multiple RBDs with SARS-CoV-2 spike trimer, or potentially through interspike cross-linking.

Based on our preliminary analysis of LM18 and its interesting breadth of neutralization for both SARS-CoV-1 and SARS-CoV-2 PSVs, we choose to construct bispecific designs around LM18. Deep sequencing analysis found that LM18 competed with CR3022 and CC12.1 but not CC6.30, suggesting that it may target an epitope similar to ADG20 (34). This specificity indicated that class 2 nanobodies (compete with CC6.30 only) should be capable of biparatopic RBD engagement when paired with LM18, forming a “clamp” that engages the two opposite faces of RBD. We also hypothesized that some class 4 nanobodies (compete with CR3022 only) may also be able to simultaneously engage with LM18, as LM18 binned to the compete bin for both CR3022 and CC12.1 (Fig. 3*D*), thus likely targets the edge of the CR3022 epitope. All nanobodies showing both specific RBD binding and monodispersed size-exclusion profiles by SEC were reformatted into the bsNb_4_-Ig format with a class 2 or 4 nanobody as the HC, and LM18 as the LC. SARS-CoV-2 RBD binding was first confirmed by ELISA (*SI Appendix*, Figs. S6 and S7) and then tested in a SARS-CoV-2 PSV neutralization assay. Sixteen out of the 20 Nb-C2/LM18 bsNb_4_Igs and 13 out of the 23 Nb-C4/LM18 bsNb_4_-Igs showed at least a 10-fold (1 µg/mL, 7 nM) and up to a 470-fold (14 ng/mL, 0.14 nM) improvement in neutralization IC_50_ compared to LM18-Fc, demonstrating that the additional interactions can produce large gains in neutralization potency (Fig. 3*F* and *SI Appendix*, Table S3).

**Fig. 4.**
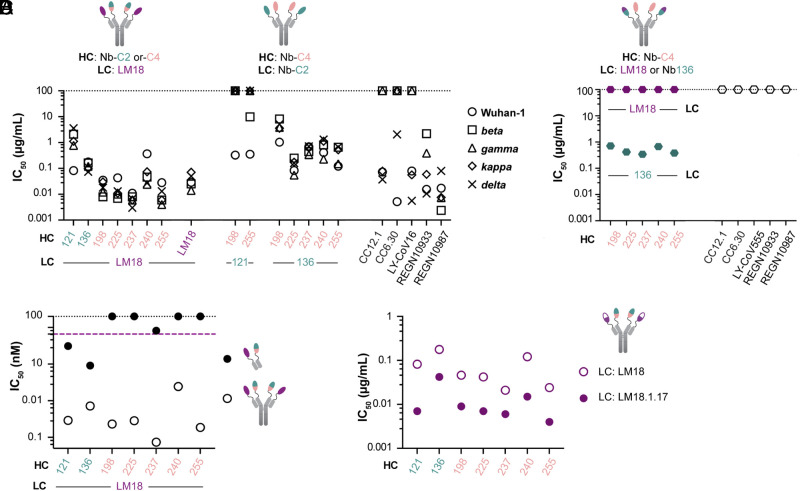
Neutralization of PSV variants by bsNb_4_-Igs. (*A*) Neutralization of SARS-CoV-2 PSVs by LM18-based bsNb_4_-Igs, LM18-Nb_4_-Ig, and Nb-C2/Nb-C4 bsNb_4_-Igs. IC_50_ values of clinical antibody candidates (LY-CoV16, REGN10933, and REGN-10987), CC12.1, and CC6.30 from our previous study (35) were added for reference. (*B*) Neutralization of *omicron* PSV by LM18- or Nb-C2-136-based bsNb_4_Igs. *Omicron* escaped from the clinical antibody candidates LY-CoV555, REGN-10933, and REGN-10987 and the monoclonal antibodies CC12.1 and CC6.30 (36). (*C*) Comparison between bsNb_4_-Ig and the corresponding Fabs, with IC_50_ as molar concentration. The black and purple dotted lines indicate the absence of neutralization (≥100 µg/mL) and the IC_50_ value of LM18-Fc, respectively. (*D*) Neutralization of Wuhan-1 SARS-CoV-2 PSV by LM18-based and affinity-matured LM18-based bsNb_4_-Igs.

A selection of the 10 most potently neutralizing bsNb_4_-Igs (Fig. 3*F*) was then assessed on a panel of SARS-CoV-2 PSV variants (*SI Appendix*, Table S4). Initially, all 10 bsNb_4_-Igs were screened against PSV variants containing single L452R (present in *kappa* and *delta* variants) and E484Q (present in *kappa* variant) point mutations. Two out of the five Nb-C2/LM18 bsNb_4_-Igs and all five Nb-C4/LM18 bsNb_4_-Igs neutralized these variants. This neutralization profile was consistent with the epitope binning data, as the tested mutations disrupt class 2 antibody (e.g., CC6.30) binding (1), but also demonstrated that the nanobodies within an epitope have subtly different specificities that can enhance or reduce their susceptibility to antigenic variability. The seven bsNb_4_-Igs that neutralized the L452R and E484Q mutants were then tested against SARS-CoV-2 VOC PSVs: *beta* (B.1.351), *gamma* (P.1), *kappa* (B.1.617.1), and *delta* (B.1.617.2) variants (Fig. 4*A*). Although the neutralization potency varied across the bsNb_4_Igs in our panel, 6/7 in our panel neutralized all 5 PSVs with an IC_50_ < 1 µg/mL. Several of the bsNb_4_-Ig where LM18 was paired with a class 4 nanobody (LM18 as LC and Nb-C4-198, Nb-C4-225, Nb-C4-237, or Nb-C4-255 as HC) neutralized all the PSVs with an average IC_50_ of 30 ng/mL (0.2 nM).

We next constructed seven bsNb_4_-Igs from class 2 and 4 building blocks, predicting that these should also be capable of the biparatopic clamping interaction made by the Nb-C2/LM18 bsNb_4_-Igs. Nb-C2-121 or Nb-C2-136 was combined with nanobodies Nb-C4-198, Nb-C4-225, Nb-C4-237, Nb-C4-240, and Nb-C4-255. All the five of Nb-C2-136-based bsNb_4_-Igs could neutralize the five viral variants, albeit less potently than the clinical antibodies and the LM18-based bsNb_4_-Igs (Fig. 4*A* and *SI Appendix*, Table S5). When the *omicron* variant (B.1.1.529) was reported, all bsNb_4_-Igs mentioned here were tested and we found that bsNb_4_-Igs with Nb-C2-136 on one arm and an Nb-C4 on the other could neutralize this new variant. *Omicron* was resistant to LM18 and all of the bispecifics that included LM18 (Fig. 4*B* and *SI Appendix*, Fig. S9 and Table S6).

### Neutralization Potency Gains from Tetravalent-Ig Format.

Several of the bsNb_4_-Igs constructs were able to achieve neutralization breadth and potency comparable to best-in-class antibodies recovered from SARS-CoV-2 convalescent donors (37, 38), and we wanted to better understand what enabled this activity. First, we tested all eight of the nanobody-building blocks (LM18, Nb-C2-121, Nb-C2-136, Nb-C4-198, Nb-C4-225, Nb-C4-237, Nb-C4-240, and Nb-C4-255) from our most potent bsNb_4_-Igs expressed as homomeric Fc fusions for the ability to neutralize Wuhan-1 SARS-CoV-2 PSV. Fc-fused LM18 and Nb-C2-136 neutralized, while the other six nanobodies-Fc (Nb-C2-121, Nb-C4-198, Nb-C4-225, Nb-C4-237, Nb-C4-240, Nb-C4-255) did not at a maximum concentration of 100 µg/mL (2.5 µM), ruling out the possibility that our tetravalent bsNb_4_-Ig potency was primarily due to one of the nanobodies with potent neutralizing ability (*SI Appendix*, Fig. S10). Next, cocktails with equimolar equivalents of LM18-Fc and either Nbs-C2-Fc or Nbs-C4-Fc were tested (*SI Appendix*, Fig. S10). The neutralization potency of these cocktails was roughly equivalent to that of the LM18-Fc fusion alone, indicating that the increased potency was not due to synergy between the two nanobody specificities in monospecific format but required them to be on a single molecule in the bsNb_4_-Igs.

To test the contributions of the two nanobody specificities formatted into a single molecule, molecular “Fabs” were produced that consisted of a single arm from the bsNb_4_-Igs. Bispecific nanobody-based Fabs with LM18 fused to the C_L_1 domain and one of the class 2 nanobodies (Nb-C2-121 or Nb-C2-136) fused to the C_H_1 domain showed modest increase in neutralization potency compared to the homomeric nanobody-Fc format, (Fig. 4*C*). In contrast, four of the five bispecific Fabs with an Nb-C4 (Nb-C4-198, Nb-C4-225, Nb-C4-237, Nb-C4-240, or Nb-C4-255) fused to the C_H_1 domain failed to neutralize at concentrations of 100 µg/mL (1.9 µM), and the sole variant that showed any neutralization (Fab Nb-C4-237/LM18) had reduced potency compared to the LM18 homomeric Fc fusion. These findings were unexpected, as the Nb-C4/LM18 bsNb_4_-Igs were more potent than the Nb-C2/LM18 bsNb_4_-Igs (Fig. 3*A*) despite the fact that class 4 nanobodies and LM18 cannot engage the same RBD simultaneously (*SI Appendix*, Fig. S8).

Lastly, we expressed the tetravalent Igs with four copies of the same nanobody (Nb_4_-Igs). In this format (Fig. 4*A*), LM18-Nb_4_-Ig showed a 340-fold increase in potency compared to the LM18 bivalent homomeric Fc fusion against Wuhan-1 SARS-CoV-2 PSV (IC_50_ of 0.029 µg/mL or 0.2 nM). Nb-C2-136-Nb_4_-Ig also showed a 20-fold increase in neutralization potency over the corresponding bivalent homomeric Fc fusion with an IC_50_ of 0.36 µg/mL (2.4 nM) for SARS-CoV-2 PSV. Nb-C2-121-Nb_4_-Ig, Nb-C4-225-Nb_4_-Ig, Nb-C4-240-Nb_4_-Ig, and Nb-246-C4-Nb_4_-Ig did not neutralize at concentrations of 100 µg/mL (*SI Appendix*, Table S7). These results highlight the LM18 specific epitope and neutralization properties, but also the advantages of bispecific constructs rather than monospecific tetravalent for neutralization. Several of the Nb-C4/LM18 bsNb_4_-Ig neutralized more potently than LM18-Nb_4_-Igs across the panel of SARS-CoV-2 PSVs, despite the inability of the Nbs-C4 to neutralize independently.

To evaluate the correlation between binding affinity and neutralization potency, we determined the binding affinities of the nanobody building blocks LM18, Nb-C2-136, and Nb-C4-225 as well as the corresponding bsNb_4_-Igs for SARS-CoV-2 RBD by surface plasmon resonance (SPR). All three of the individual nanobodies had relatively weak binding for SARS-CoV-2 RBD, with dissociation constants (*K*_D_s) between 143 nM and 391 nM and exhibiting a rapid dissociation rate, commonly observed with synthetic nanobodies from discovery libraries (*SI Appendix*, Fig. S11 and Tables S8 and S9). In the tetra-Ig format, LM18-Nb_4_-Ig and the Nb-C4-225/LM18 bsNb_4_-Ig had kinetics that were similar to the individual nanobodies (*SI Appendix*, Fig. S11). The Nb-C2-136/LM18 bsNb_4_-Ig showed a 630-fold improvement in *K*_D_ to 500 pM due to a nearly nonexistent off-rate, consistent with the epitope binning that showed LM18 and Nb-C2-136 bind to nonoverlapping epitopes and could be capable of biparatopic engagement (Fig. 3*D*). Finally, we tested the prefusion-state-stabilized SARS-CoV-2 S trimer (38) against all the three formats, all of which bound with high affinity and had a very slow dissociation rate (*SI Appendix*, Fig. S11). Of note, the Nb-C2-136/LM18 bsNb_4_-Ig had a notably slower dissociation rate compared to the other two variants, yet this construct was the least potent neutralizer of the group. The mode of binding to the S trimer for the bsNb_4_-Ig is unclear and potentially heterogeneous; however, it is noted that all tetra-Igs are able to achieve intraspike avidity and substantially reduce the apparent off-rate. There was nothing in the binding data to definitively suggest why Nb-C4/LM18-based constructs would neutralize as potently as they do, but this may be partially attributed to the RBD subunits being preferentially in the “down” conformation on the stabilized S trimers (39).

### Affinity Maturation of Nanobody Building Blocks.

All of the above work explored how the nanobody potency can be improved through modular multivalent formats. However, it is well established that increased antibody- or nanobody-binding affinity for a target antigen can also enhance neutralization potency. We hypothesized that affinity maturation of the base nanobodies would translate to an increase in potency for the resulting bsNb_4_-Igs. To test this notion, an affinity maturation library of LM18 was created using our SAMPLER maturation strategy (40), where one of all single-mutation variants of each CDR loop is generated and combined into a combinatorial library (*SI Appendix*, *Text*). This library was displayed on the surface of yeast and subjected to four rounds of FACS selections–two rounds with RBD to enrich higher affinity clones, one PSR round to deplete sticky clones, and a final round with RBD to further enrich higher affinity clones (*SI Appendix*, Fig. S12). At the conclusion of the selections, 96 colonies were submitted for Sanger sequencing. No clear consensus sequence was present; therefore, we selected 10 constructs for recombinant production as Fc fusions. The IC_50_ value of the best variant, LM18.1.17-Fc, improved neutralization potency by a factor of 6 (0.41 µg/mL or 10 nM) and 13 (0.037 µg/mL or 0.9 nM) for SARS-CoV-2 and SARS-CoV, respectively (*SI Appendix*, Figs. S4 and S13). SPR analysis of LM18.1.17 binding to RBD also identified a 6-fold improvement in binding affinity compared to the parental LM18 (*SI Appendix*, Table S8). We then incorporated LM18.1.17 instead of LM18 into the bsNb_4_-Igs format and measured the neutralization potency against SARS-CoV-2 PSV. In all cases, LM18.1.17-based bsNb_4_-Igs showed more potent neutralization compared to LM18-based bsNb_4_-Igs (Fig. 4*D* and *SI Appendix*, Fig. S14 and Table S10). As expected, affinity maturation of the base nanobody components translated to potency gains of the bsNb_4_-Igs, demonstrating that the overall function can be further improved.

### Structural Validation of Engineered Nanobody Design.

**Fig. 5.**
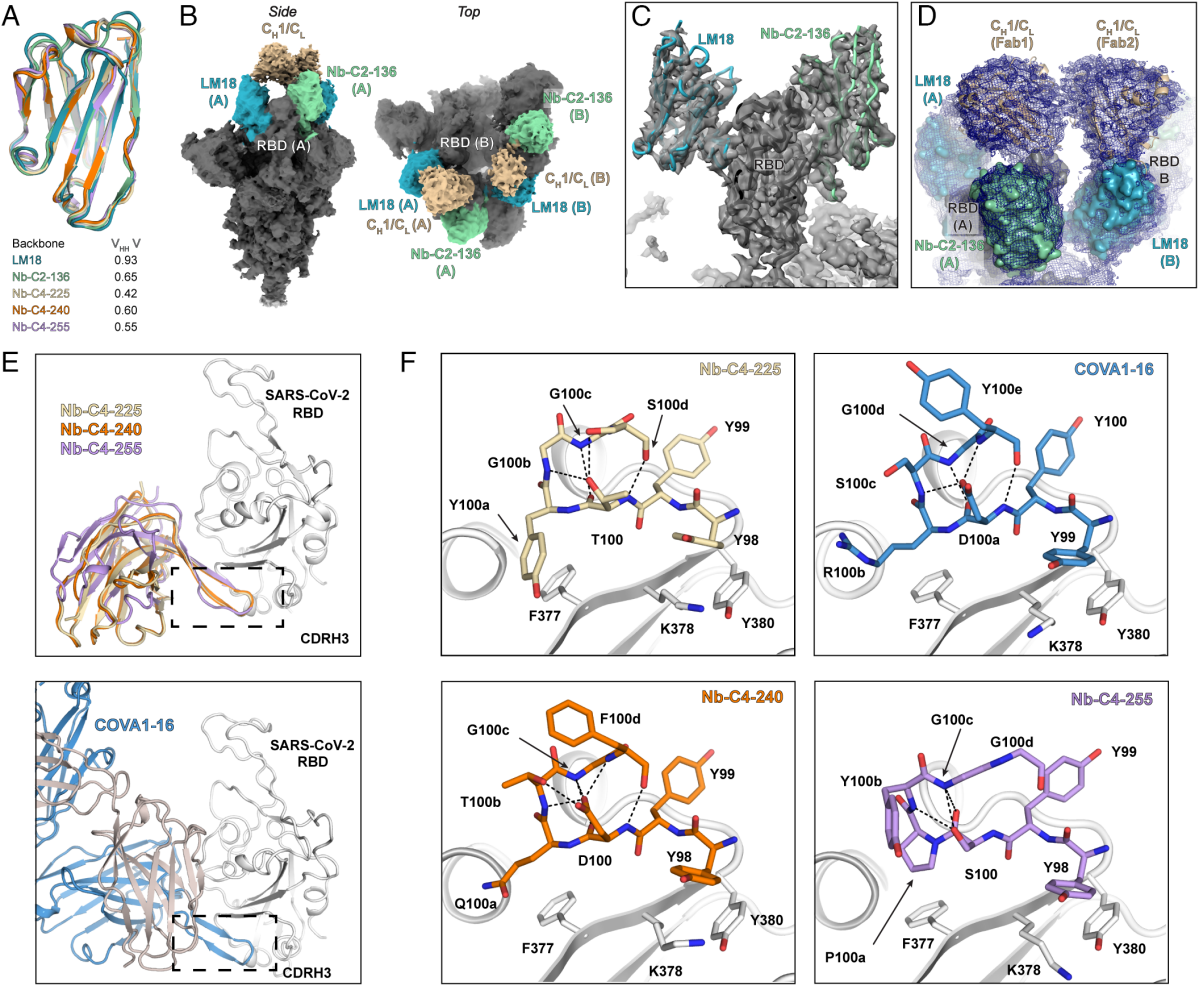
Structural validation of engineered nanobody design. (*A*) Our engineered nanobodies adopt the same backbone structure as representative llama nanobodies. Structures of engineered nanobodies (i.e., LM18, class 2 Nb-C2-136, class 4 Nb-C4-225, 240, and 255) were overlapped with V_HH_ V, a SARS-CoV-2 nanobody (PDB ID: 7KN6) isolated from an immunized llama (13). The CDRH3 of these nanobodies was not considered for rmsd calculations due to its high sequence diversity. The backbone structures of these engineered nanobodies are virtually the same as the representative llama nanobodies, V_HH_ V (rmsd<1 Å). (*B*) Cryo-EM reconstruction of LM18/Nb-C2-136 bsNb_4_-Ig in complex with SARS-CoV-2 6Pmut7 S protein colored by subunit/domain. (*C*) Cryo-EM focused classification and local refinement of a single RBD bound to LM18 and Nb-C2-136. The EM map is shown in transparent gray, with fitted atomic models in backbone tube representation. (*D*) Modeling of RBD-LM18/Nb-C2-136 Fab complex into cryo-EM reconstruction density of LM18/Nb-C2-136 bsNb_4_-Ig in complex with SARS-CoV-2 6Pmut7 S protein. The density allows for approximate (not atomic resolution) placement of C_H_1/C_L_. LM18 is in blue; Nb-C2-136 in green; C_H_1/C_L_ in wheat color. (*E*) The crystal structures of class 4 Nb-C4-225, Nb-C4-240, and Nb-C4-255 share the same binding mode to the SARS-CoV-2 RBD (*Top*). These three nanobodies bind the conserved epitope site previously described for CR3022 and COVA1-16 (41, 42). Coincidently, these nanobodies bind SARS-CoV-2 RBD in a highly similar way as YYDRxG antibodies, e.g., COVA1-16 (*Bottom*) and ADI-62113 (43). (*F*) Nb-C4-225, Nb-C4-240, and Nb-C4-255 share similar CDRH3 motifs as COVA1-16. CDRH3 motifs are shown in color and stick representation and SARS-CoV-2 RBD in gray and ribbon representation. Dashed lines indicate internal polar interactions within the CDRH3 motif.

To understand the structural integrity and epitope propensity of our engineered nanobodies, we determined atomic structures of a selection of our lead nanobodies (LM18, Nb-C2-136, Nb-C4-225, Nb-C4-240, and Nb-C4-255) in complex with SARS-CoV-2 RBD (X-ray crystallography, *SI Appendix*, Table S11) and SARS-CoV-2 S protein (cryo-electron microscopy, *SI Appendix*, Table S12). Our structures reveal that these nanobodies adopt a V_HH_ fold as expected (Fig. 5*A*). The average rmsd of protein backbone structures between our five engineered nanobodies and a representative SARS-CoV-2 camelid nanobody (PDB ID: 7KN6) is 0.63 (0.42 to 0.93) Å when the conformationally variable CDRH3 is excluded, confirming that our synthetic V_HH_ scaffold mimic naturally occurring nanobodies in terms of overall topology. The engineered Cys49 and Cys69 disulfide designed to maintain the structural stability in these nanobodies is evident in the X-ray electron density maps (*SI Appendix*, Fig. S15*A*).

The cryo-EM structure of the LM18/Nb-C2-136/ bsNb_4_-Ig in complex with trimeric S protein helped elucidate the mode of binding that facilitated the high binding affinity and potent neutralization. The structure shows LM18 and Nb-C2-136 binding to the same RBD in the up conformation with two of the three RBDs bound on the S protein (Fig. 5 *B* and *C*). Consistent with the binding data and our molecular clamp hypothesis, it appears that the LM18 and Nb-C2-136 nanobodies are linked to the same C_H_1/C_L_ domain. A small amount of density is observed in the EM map (*SI Appendix*, Fig. S15*C*), and modeling suggests that the C_H_1/C_L_ domain can adopt an ensemble of conformations with the 12 AA linkers that connect the nanobodies to the C_H_1/C_L_ domain (Fig. 5*D* and *SI Appendix*, Fig. S16*A*). No density is visible for the Fc domain; however, the orientation of the C_H_1/C_L_ domains is compatible with a single bsNb4-Ig using both arms to engage the two up RBDs on S protein (*SI Appendix*, Fig. S16*B*).

### Three Different SARS-CoV-2 RBD Epitopes Targeted by Engineered Nanobodies.

The X-ray structures of the three class 4 nanobodies, Nb-C4-225, Nb-C4-240, and Nb-C4-255, in complex with SARS-CoV-2 RBD, revealed that these nanobodies bind to the CR3022 epitope site in an approach angle similar to YYDRxG antibodies (e.g., COVA1-16 and ADI-62113) that we reported recently (42, 43)(Fig. 5*E*). The CDRH3s of these nanobodies contribute to >90% of the buried surface area on the RBD, and structural analysis showed that the CDRH3 of Nb-C4-225, Nb-C4-240, and Nb-C4-255 forms remarkably similar β-bulge structures to those in the YYDRxG antibodies (Fig. 5*F*). These structures use the same tyrosines (as YY in the YYDRxG motif) to form hydrophobic and π–π interaction with highly conserved residues in the SARS-CoV-2 RBD. Glycine at 100c (corresponding to G100d in COVA1-16 CDRH3) also shifts the following residue from a down to an up register as also reported in the ADI-62113 crystal structure (43). Meanwhile, similar hydrogen bonds and salt bridges are used to maintain these β-bulge structures. Structural superimposition of these nanobodies with COVA1-16 showed that these residues are positioned in the same location in binding to the highly conserved neutralizing epitope site of SARS-CoV-2 RBD (*SI Appendix*, Fig. S15*B*). Although those three nanobodies did not compete with ACE2 binding due to their smaller size compared to COVA1-16 and other YYDRxG antibodies (*SI Appendix*, Fig. S8), combination of these nanobodies with LM18 or Nb-C2-136 in the bsNb_4_-Ig format demonstrated broad neutralization against SARS-CoV-2 variants, including *omicron* (Fig. 4 *A* and *B*).

### Structural Validation of On-Yeast Epitope-Binning Strategy.

**Fig. 6.**
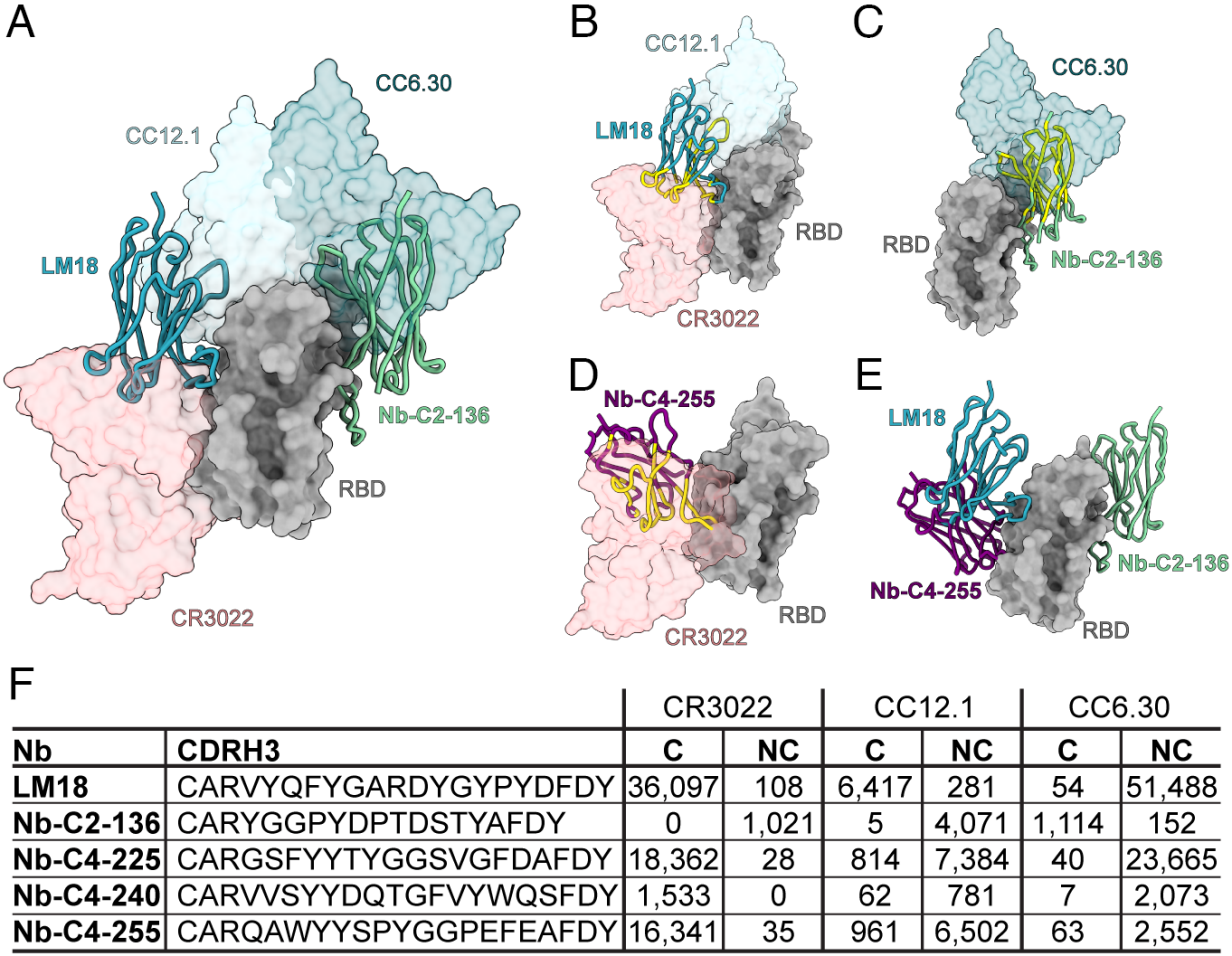
Structural validation of on-yeast epitope binning. (*A*) Binding of LM18 and Nb-C2-136 with respect to antibodies representing the three epitopes used for FACS-based binding. Nanobodies were displayed as ribbons and binning epitope antibodies as transparent surfaces. Comparison of (*B*) LM18-, (*C*) Nb-C2-136-, and (*D*) Nb-C4-255-binding sites on RBD with representative neutralizing antibodies (space-filled structures). Nanobody atoms predicted to clash with representative antibodies are colored in yellow. (*E*) Three different classes of nanobodies (LM18, Nb-C2-136, and Nb-C4-255) in complex with RBD. (*F*) NGS analysis and CDRH3 sequences from competitive sorts with CC12.1, CR3022, and CC6.30. Counts for C and NC population with the three antibodies are indicated.

An atomic model of LM18 and Nb-C2-136 was generated using focused classification and local refinement methods on a single RBD in the cryo-EM data (Fig. 5 *B*–*D* and *SI Appendix*, Fig. S15*C*). LM18 binds an epitope between the class 1 (CC12.1) and class 4 (CR3022) binding sites in a manner that is predicted to sterically clash with and compete with these two antibodies (Fig. 6 *A*, *B*, and *E* and *SI Appendix*, Fig. S15). Similar to the class 4 nanobodies, approximately 88% of the buried surface area is contributed by CDRH3. Nb-C2-136 binds on the opposite face of the RBD in a region that would sterically clash with class 2 antibodies (e.g., CC6.30) (Fig. 6 *A*, *C*, and *E*). Unlike the other nanobodies, two CDRs are involved with binding to RBD, with CDRH2 and CDRH3 contributing approximately 30% and 50%, respectively, to the buried surface area. The epitopes of all five nanobodies, as determined by high-resolution structural analysis, validate the FACS-based epitope-binning strategy used for selection and engineering (Fig. 6 *B*–*E*).

## Discussion

The use of recombinantly produced monoclonal antibodies for the prophylaxis and/or therapy of RSV, Ebola virus, and SARS-CoV-2 infections has provided the foundation for a novel class of antivirals. Single-domain nanobodies offer many of the same beneficial properties of monoclonal antibodies: high binding affinity, target specificity, and, when expressed as Fc fusions, long in vivo half-life via FcRn recycling and Fc effector functions. Additionally, nanobodies offer some unique opportunities owing to their small size and the ease with which they can be multimerized. Single-domain nanobodies can access cryptic epitopes on immunization-resistant viruses, such as HIV, that are sterically less accessible to two domain antibodies (4). It also has been well established that oligomerization of nanobodies to create multimers that can simultaneously engage multiple epitopes can produce substantial gains in binding affinity and neutralization potency (6, 9, 10, 13, 15). These two distinct properties of nanobodies allow for the rational design of antivirals that can overcome some of the mechanisms that viruses use to evade antibody-mediated neutralization.

Our bsNb4-Ig platform was designed to leverage the power of nanobodies—specifically the creation of heterodimers for biparatopic target engagement—in an antibody-like molecule. The modular nature of the chain pairing enabled the rapid production and functional screening of nanobody combinations to find productive pairs. In this work, all bsNb4-Igs were expressed as human IgG1; however, the V_H_/V_L_ heterodimerization strategy should be compatible with any IgG isotype, so the Fc domain can be selected for the intended in vivo application and can include mutations to extend half-life and/or modulate effector functions. Our human-based nanobody library provides a source of biochemically well-behaved nanobodies with minimal modifications from a starting human antibody V_H_ gene, potentially reducing immunogenicity compared to camelid-derived nanobodies. The resulting tetra-specific nanobody-based constructs achieved a neutralization potency on par with SARS-CoV-2 antibodies isolated from convalescent donors, some in the ng/ml range (37, 38); meanwhile, constructs with more modest IC_50_ values showed breadth of neutralization across all tested variants (Fig. 4 *A* and *B*). The bsNb_4_-Ig format also contributed significantly to overall efficacy. As an Fc fused dimer, the nanobody LM18 neutralized SARS-CoV-2 but had a 340-fold potency increase when expressed as an Nb_4_-Ig homotetramer and a 175- and 470-fold increase when expressed as a bsNb4-Ig heterotetramer with the best class 2 and 4 nanobodies, respectively. We had hypothesized that the best combinations would either be Nb-C2/LM18 or Nb-C2/Nb-C4, where both nanobodies could simultaneously engage RBD. Unexpectedly, Nb-C4-237/LM18 was our most potently neutralizing variant despite the two nanobodies being sterically unable to simultaneously engage the same RBD (*SI Appendix*, Fig. S8).

Another unexpected finding was that Nb-C4-237 does not neutralize as an Nb_4_-Ig homotetramer, but the combination of this nonneutralizing nanobody with the neutralizing LM18 was more effective than the LM18 Nb_4_-Ig homotetramer (with an average of 10-fold more potent across the panel of VOCs). It is not clear why this combination was so effective in the bsNb_4_-Ig format, but these results highlight the need to have a platform that allows rapid experimental validation of different combinations as they cannot necessarily be predicted. Another unexpected finding with the class 4 nanobodies 225, 240, and 255 was their convergent mode of binding (Fig. 5 and *SI Appendix*, Fig. S15*B*). The YYDRxG-like motif ([YY(D/T/S)xxG]) in the CDRH3 found in these three nanobodies was also identified in several antibodies isolated from different convalescent COVID donors (43). While the concept of a shared antibody response is not new, previous examples of structurally convergent antibody/antigen solutions could often be attributed to biases in naïve antibody repertoire due to the limited number of immune gene segments that rearrange during B cell development. Structurally conserved responses against the influenza HA stem (44), HIV CD4–binding site (45), malaria major CSP repeat region (46), and SARS-CoV-2 RBD have been reported (47), where antibodies isolated from different donors achieve a nearly identical mode of recognition through contacts encoded largely in the starting V_H_ gene. Examples of CDRH3 convergence have also been reported, such as for HIV apex-targeting antibodies (48); for influenza hemagglutinin receptor–binding site binders (49), and more recently the SARS-CoV-2 RBD YYDRxG–targeting antibodies (43) where a shared D gene segment encodes many of the critical contacts. Neither of these biases can explain the convergence observed with the class 4 nanobodies, as the constant V_HH_ region does not make significant contacts with RBD and the CDRH3 diversity was randomly generated using mixed trimer phosphoramidites during oligonucleotide synthesis. Here, a synthetic antibody/nanobody has converged to the same structural motif as a B cell–derived antibody/nanobody. We also note that there are additional nanobody sequences that bin with the CR3022 site that contain a YYDRxG-like motif (*SI Appendix*, Fig. S17). These data would suggest that the convergent binding solutions are more attributed to the geometric compatibility of this epitope, i.e., the formation of the backbone hydrogen–bonding network and aromatic packing, than to the presence of starting antibody gene segments. It also raises the possibility that epitope immunodominance may be largely dictated by the “ease of targetability,” where certain protein surfaces are just more geometrically compatible to form extended interaction networks with the spacing of AAs coming off from a normal beta-hairpin CDRH3 loop.

In summary, our platform enables the incorporation of individual nanobody binders into an IgG-like scaffold, and the resulting biparatopic constructs present an equivalent neutralization potential to canonical antibodies (37, 38). Additionally, this approach offers the possibility to further affinity mature each building block separately, and therefore increase potency. We rapidly identified nanobodies targeting specific epitopes from our naïve library using on-yeast surface epitope binning. This workflow and the resulting bsNb_4_-Ig provide a unique biologic that can target viruses in ways not possible with conventional antibodies.

## Materials and Methods

### Discovery of Specific SARS-CoV-2 RBD Binders.

We used a combination of three rounds of MACS to deplete the library of nonbinding clones, with five rounds of FACS to enrich the population in nanobodies that target SARS-CoV-2 RBD. Specific details can be found in *SI Appendix*, *Materials and Methods*.

### hV_HH_323 Sequencing and Analysis.

Libraries were deep sequenced to determine the CDRH3 at each round of selection on an Illumina MiSeq (Illumina Inc., San Diego, CA, USA) with the paired-end MiSeq v2 500 bp kit. Paired-end fastqs were analyzed for sequence quality and forward and reverse reads were merged, then clustered in groups with fully identical sequences. Specific details can be found in *SI Appendix*, *Materials and Methods*.

### Protein Expression and Purification.

All recombinant soluble proteins from SARS-CoV, SARS-CoV-2, and their truncated protein versions (RBD) were expressed and purified as previously described (38). All antibodies, nanobodies, and nanobody-based constructs were expressed and purified from Expi293F (Thermo Fisher Scientific), and methods for purification are described in *SI Appendix*, *Materials and Methods*.

### PSV Assay.

PSV assays were performed according to the protocol described by Roger et al. (38).

### Crystallization and X-Ray Structure Determination.

The RBD-Nb-C4-CC12.1 complexes were crystallized by vapor diffusion using the hanging drop method. All X-ray datasets were collected at the Stanford Synchrotron Radiation Lightsource (SSRL) and the Advanced Photon Source (APS) at Argonne National Laboratory. Information regarding X-ray data indexing, processing, and model building is described in *SI Appendix*, *Materials and Methods*.

### Cryo-EM Sample Preparation and Data Acquisition.

Trimeric SARS-CoV-2 6P-Mut7 S protein was incubated with a threefold molar excess of LM18/Nb-C2-136 bsNb_4_-Ig at room temperature for 100 min at a concentration of 0.85 mg/mL as determined by A_280_. n-dodecyl-β-D-maltopyranoside was added to a final concentration of 0.06 mM and the sample deposited on plasma-cleaned Quantifoil 1.2/1.3 300 mesh grids. A Thermo Fisher Vitrobot Mark IV set to 4 °C, 100% humidity, 3 s wait time, and a 3 s blot time was used to vitrify samples in liquid ethane. Data were collected on a Thermo Fisher Titan Krios operating at 300 keV and equipped with a Gatan K2 Summit direct electron detector. Information regarding processing and model building is described in *SI Appendix*, *Materials and Methods*.

## Supplementary Material

Appendix 01 (PDF)

## Data Availability

X-ray coordinates and structure factors were deposited in the RCSB Protein Data Bank (PDB) under accession code 8ELO for Nb-C4-225 in complex with SARS-CoV-2 RBD and CC12.1 Fab (50), 8ELP for Nb-C4-240 in complex with SARS-CoV-2 RBD and CC12.1 Fab (51), and 8ELQ for Nb-C4-255 in complex with SARS-CoV-2 RBD and CC12.1 Fab (52). Cryo-EM maps were deposited to the Electron Microscopy Data Bank (EMDB) under accession codes EMD-27692 (focused refinement) (53) and EMD-27693 (global refinement) (54). Atomic coordinates for the focused refinement model were deposited to the PDB under accession code 8DT8 (55). Some study data are available: the plasmids described in the manuscript will be available by MTA.
