# Supplementary material for: Fully synthetic platform to rapidly generate tetravalent bispecific nanobody–based immunoglobulins
Source: Proc Natl Acad Sci U S A. 2023 Jun 5;120(24):e2216612120. doi: 10.1073/pnas.2216612120 (PMC10268213; doi:10.1073/pnas.2216612120)
Supplement: Supplementary file 1 — Appendix 01 (PDF) [file pnas.2216612120.sapp.pdf]

**Supplementary Information for**
# Fully synthetic platform to rapidly generate tetravalent bispecific nanobody-based immunoglobulins

Laetitia Misson Mindrebo[1,2,9], Hejun Liu[3,9], Gabriel Ozorowski[1,3,4,9], Quoc Tran[1,2], Jordan Woehl[1,2], Irene Khalek[1,2], Jessica M. Smith[2], Shawn Barman[1,4,5], Fangzhu Zhao[1,4,5], Celina Keating[1,4,5], Oliver Limbo[1,2], Megan Verma[1,2], Jingjia Liu[6], Robyn L. Stanfield[3], Xueyong Zhu[3], Hannah L. Turner[1,3,4], Devin Sok[1,2,4,5], Po-Ssu Huang[6], Dennis R. Burton[1,4,5,7], Andrew B. Ward[1,3,4], Ian A. Wilson [3,8], Joseph G. Jardine*[1,2]

[1] IAVI Neutralizing Antibody Center, The Scripps Research Institute, La Jolla, CA 92037, USA
[2] IAVI, New York, NY 10004, USA
[3] Department of Integrative Structural and Computational Biology, The Scripps Research Institute, La Jolla, CA 92037, USA
[4] Consortium for HIV/AIDS Vaccine Development (CHAVD), The Scripps Research Institute, La Jolla, CA 92037, USA
[5] Department of Immunology and Microbiology, The Scripps Research Institute, La Jolla, CA 92037, USA
[6] Department of Bioengineering, Stanford University, Stanford, CA 94305, USA
[7] Ragon Institute of Massachusetts General Hospital, Massachusetts Institute of Technology, and Harvard University, Cambridge, MA 02139, USA
[8] Skaggs Institute for Chemical Biology, The Scripps Research Institute, La Jolla, CA 92037, USA
[9] These authors contributed equally to this work

* Corresponding author: Joseph G. Jardine
**Email:** jjardine@iavi.org

**This file includes:**

Supplementary text
Figures S1 to S17
Tables S1 to S14
SI References

## Supplementary Information Text

**Library construction.** The diversity in the CDRH3 was introduced with oligonucleotides synthesized with trimer phosphoramidite mixtures based on the frequencies of amino acids (AAs) found in antibody CDR3 sequences (originally synthesized for the production of a human antibody library, Table S13). All CDRH3 sequences begin in CAR and end in FDY, with 5-15 randomized AAs between the consensus residues. The oligonucleotides that were used, with [TriMix1], [TriMix2], [TriMix3], [TriMix4] and [TriMix5] representing different trimer phosphoramidite mixtures are shown in Table S14.

Double-stranded DNA was generated by combining the CDRH3 oligonucleotides with an invariant oligonucleotide encoding the nanobody framework 4 segment and 3' homology for the pYDSI2u_SiDir vector
(CTCCTAGGAGTTCAGGTGCTGGTGATGGAGGTGACGTGTGAGTCTTGTCACCGGATCCAG
ATGAAACAGTGACCTGCGTACCTTGTCCCCAGTAGTCG). This CDRH3/FW4 fragment was then combined with the invariant 5' fragment consisting of the $hV_{HH}323$ (Fig. S1, codon optimized for yeast expression) and 5' homology for the pYDSI2u_SiDir vector
(GGTTTGTCATCTACAAATACAACAATCGCATCCATAGCAGCTAAAGAGGAGGGTGTTCAGCT
GGACAAGAGAGAAGCTAGTGAAGTTCAATTGCAAGAATCTGGTGGTGGTTTGGTTCAACCAG
GTGGTTCTTTGCGTTTGTCTTGTGCTGCGTCTGGTTTTACTTTTTCTTCTTATGCTATGGGTT
GGTATAGACAAGCTCCAGGTAAAGAAAGAGAATGGGTTTGTGCTATTTCCGGTTCTGGCGGT
TCTACTTATTATGCTGATTCTGTTAAAGGTAGATTTACTTGTTCTAGAGATAATTCTAAAAACA
CTTTGTATCTTCAAATGAATTCTTTGAAGCCAGAGGACACAGCTGTCTACTACTGCGCC)
using isothermal assembly. Upon assembling the full nanobody library containing the necessary 5' and 3' homology for the target vector, the DNA was amplified and PCR cleaned for transformation. Each CDRH3 length was prepared independently and mixed at the desired distribution prior to transformation (Fig. S2).

The nanobody library was then cloned into pYDSI2u_SiDir via homologous recombination in *Saccharomyces cerevisiae* YVH10 cells (ATCC, MYA4940) using protocol described in the reference (1). A total of 32 transformations were performed and pooled to achieve the desired library size. Dilutions of transformed yeast were then plated on dropout medium without uracil (SD–Ura Sunrise Science) as single colonies to obtain the estimate of library diversity of $3x10^9$ unique clones.

The plasmids described in the manuscript will be available by MTA.

## Supplementary materials and methods

**Magnetic-activated cell sorting (MACS) of naïve library.** Initially, 400 µL of Super Mag Streptavidin Beads (50 nm diameter Ocean Nanotech) were pre-coupled with biotinylated SARS-CoV-2 RBD for 30 min at 4 °C in PBS, 2% (w/v) bovine serum albumin (PBSA 2%). $2 \times 10^{11}$ induced yeast cells from our naïve library (oversampling our library by a factor of ~100) were subsequently washed with PBSA 2% and incubated with the pre-coupled magnetic beads o/n at 4 °C. All three rounds of MACS were performed on an autoMACS Pro Separator using autoMACS columns (Miltenyi Biotec). In the first round of MACS (positive selection, Posseld2 program), 20 runs of $1 \times 10^{10}$ cells were sorted and the binders to the magnetic beads were selected and grown in SD-Ura o/n at 30 °C, and the following day induced o/n at 30 °C in in SGCAA + Trp induction medium (20 g galactose, 1 g glucose, 6.7 g yeast nitrogen base without amino acid, 5 g bacto casamino acids, 5.4 g $Na_2HPO_4$, 8.56 g $NaH_2PO_4 \cdot H_2O$, 8.56 mg Trp in 1 L deionized water, pH 6.5, sterilized by filtration). To remove yeast-expressing nanobody that bound nonspecifically to magnetic beads, we performed a negative selection using only the magnetic beads. $2 \times 10^{10}$ induced cells were incubated with 200 µL Super Mag Streptavidin Beads in PBSA 2% for 30 min at 4 °C. The yeast cells that didn't bind to the magnetic beads were selected (negative selection, Possel program) and subsequently incubated with pre-coupled magnetic beads to RBD (as described above) o/n at 4 °C. For the third and final round of MACS (positive selection, Posseld2 program), binders to the magnetic beads were selected and grown in SD-Ura o/n at 30 °C.

**Fluorescence-activated cell sorting (FACS).** FACS is used after depleting the library of non-binding clones by MACS to enrich the yeast cells in RBD-specific clones. *In vitro* engineering of antibodies or nanobodies can lead to constructs that are polyspecific (2), so we alternated between positive and negative selections to enhance the specificity of our synthetic constructs. The cells were alternatively selected as RBD binders (affinity sorts, AFF), or depleted against a biotinylated preparation of detergent solubilized biotinylated membrane proteins (polyspecific reagent or PSR) non-binders (negative sorts, PSR). In each round of selection, 1-5 x$10^7$ induced yeast cells were incubated for 60 min at 4 °C (rotating at 50 rpm) with biotinylated SARS-CoV-2 RBD for AFF sorts, or biotinylated HEK-cell soluble membrane protein extracts (3) for PSR sorts in 500 µl 1% PBSA (PBS containing 1% BSA) or PBS, respectively. Yeast cells were then washed twice with 1% PBSA (affinity sorts) or PBS (PSR sorts) and coupled to 2 fluorophores (1µg/mL) for 20 min: anti-V5-AF405-conjugated to check the yeast display, and anti-biotin-APC or streptavidin-PE to check RBD binding (we alternated the use of biotin-specific secondary antibody to prevent the enrichment in secondary antibody/fluorophore specific clones). Yeast cells were then washed once with PBSA 1% and resuspended in 1 mL PBSA 1% for sorting on a FACS Melody (BD Biosciences). Selected yeast cells were sorted into SD-Ura medium, grown and induced for consecutive rounds of selection. For AFF1, AFF2 and AFF3, 100, 20 and 4 nM of biotinylated SARS-CoV-2 RBD were used, respectively. For PSR1 and PSR2, 10 µg of biotinylated HEK-cell soluble membrane protein extracts were used. Sorted cells were either prepared for NGS sequencing, or serial dilutions of the last affinity sorts were plated on SD-Ura agar. After 3 days at 30°C, DNA of single colonies was amplified using Phire Plant PCR kit (Thermo Fisher) and sent for Sanger sequencing.

**h$V_{HH}$323 sequencing and analysis.** Libraries were deep sequenced to determine the CDRH3 at each round of selection**.** The DNA from the sorted yeast cells was miniprepped (Qiagen) in the presence of zymolyase (Zymo Research) and amplified through two rounds of PCR as previously described (1). The first PCR reaction generates a ~ 200 bp amplicon containing flanking universal Nextera sequencing adapters using a set of six primers: hNb323_NGSSeq_Fa, hNb323_NGSSeq_Fb, hNb323_NGSSeq_Fc, hNb323_NGSSeq_Ra, hNb323_NGSSeq_Rb and hNb323_NGSSeq_Rc (Table S14). The second round of PCR adds a specific index primer pair (i5/i7) so the library could be pooled, cleaned, and sent for deep sequencing on an Illumina MiSeq (Illumina Incorporated, San Diego CA) with the paired-end MiSeq v2 500 bp kit.

**Deep sequencing analysis.** Paired-end fastqs were analyzed for sequence quality using the FastQC package (FastQC v0.11.9) (4). The forward and reverse reads were merged using BBMerge (version 38.87) from the BBTools suite using the default parameters (5). Merged reads were clustered using VSEARCH (v2.15.1) to quickly group reads with fully identical sequences (6). Clustering was done using the "cluster_fast" method and fasta files were written including the abundance of each unique sequence in the fasta header. This step substantially improved performance of downstream fasta parsing as each unique sequence was only analyzed once. A custom python (Python 3.7) script was written to parse the clustered fasta output, remove primer sequences, and translate the DNA sequences to amino acid sequences. The script then quantified the unique CDRH3 positions.

**NGS analysis of the on-yeast epitope binning** (Table S2). The deep sequencing datasets were concatenated based on the CDRH3 sequence. To remove likely sequencing errors, a filter was applied to remove nanobodies that did not appear in either the C (compete) or the NC (noncompete) datasets of at least two of the antibody datasets. Additionally, nanobodies that had <10 counts were also removed. With these criteria, a total of 123 unique nanobody CDRH3s were obtained. Epitope bins were assigned by defining an overlapping epitope with a tested SARS-CoV-2 antibody as having a C/NC (compete/noncompete) ratio >10, and NC/C ratio >10 for a nonoverlapping epitope. All analysis was based on raw sequencing count data.

**Affinity-maturation of LM18.** To select high-affinity nanobody variants, an affinity maturation library was prepared based on our previously reported SAMPLER strategy (1). The size of the theoretical starting library was 4.2 x $10^6$ unique nanobodies (138 CDRH1 variants, 116 CDRH2 variants and 261 CDRH3 variants). We also included an M34L mutation that we identified as being

potentially stabilizing. The nanobody library was cloned into pYDSI2u_SiDir using homologous recombination as described above. Four rounds of FACS-based selection (AFF1-AFF2-PSR1-AFF3) were performed to isolate populations of high-affinity clones. Serial dilutions of the AFF3 sort were plated on SD-Ura agar. After 3 days at 30°C, DNA of 96 single colonies was amplified using Phire Plant PCR kit (Thermo Fisher) and sent for Sanger sequencing.

**Protein expression and purification.** All recombinant soluble proteins from SARS-CoV, SARS-CoV-2 and their truncated protein versions (RBD) were expressed and purified as previously described (7).

**Nanobody and antibody expression and purification.** Nanobodies-Fc and antibodies (HC and LC constructs) were transiently expressed with the Expi293 Expression System (Thermo Fisher). After five days, 24-deep well culture supernatants were harvested and purified using protein A magnetic beads (Thermo Fisher) and tested for binding and neutralization. Selected nanobodies and antibodies were re-expressed in small to medium scale cultures and IgG-purified on Protein A sepharose (GE Healthcare). Constructs were buffer exchanged in PBS and stored at 4°C. $His_{10}$-tagged Nbs used for SPR and crystallization were purified with the HisPur Ni-NTA Resin (Thermo Fisher). To eliminate nonspecific binding proteins, each column was washed with at least 3 bed volumes of wash buffer (25 mM Imidazole in TBS, pH 7.4). To elute the purified proteins from the column, we used five bed volumes of elution buffer (250 mM Imidazole in TBS, pH 7.4). Constructs were buffer exchanged in TBS and stored at room temperature.

**Recombinant Protein ELISAs**. Anti Histag monoclonal antibody (Invitrogen, MA1-21315-1MG) was coated onto high-binding 96-well plates (Corning, 3690) at 2 µg/mL overnight at 4 °C. After washing, plates were blocked with PBSA 3% (3% BSA in PBS) for 1 h. Then $His_{10}$-tagged recombinant RBD or spike protein were captured at 1 µg/mL in PBSA 1% and incubated for 1 h at room temperature. After washing, serially diluted nanobodies or antibodies were added into wells and incubated for 1 h at room temperature. Detection was measured with alkaline phosphatase-conjugated goat anti-human IgG Fcγ (Jackson ImmunoResearch 109-005-008) at 1:1000 dilution for 1h. After the final wash, phosphatase substrate (Sigma-Aldrich, S0942-200TAB) was added into wells. Absorption was measured at 405 nm after less than 15 min. Positive and negative controls were systematically used. Non-linear regression curves were plotted using Prism 8 software.

**Polyspecificity reagent ELISA**. According to the protocol described by Roger *et al*. (7), solubilized CHO-cell membrane proteins (SMP) and single strand (SS) DNA (Sigma-Aldrich, D8899) were used. SMP or SS were coated onto 96-well half-area high-binding ELISA plates (Corning, 3690) at 5 µg/mL in PBS overnight at 4˚C. After washing, plates were blocked with PBSA 3% for 1 h at RT. Antibody samples were diluted at 100 µg/mL in PBSA 1% with serial dilution and then added in plates and incubated for 1 h at RT. After washing, alkaline phosphatase-conjugated goat anti-human IgG Fcγ secondary antibody (Jackson ImmunoResearch, 109-055-008) was added in 1:1000 dilution and incubated for 1h at RT. After final wash, phosphatase substrate (Sigma-Aldrich, S0942-200TAB) was added into each well. Absorption was measured at 405 nm after 15 min.

**Pseudovirus (PSV) Assay.** PSV assays were performed according to the protocol described by Roger *et al*. (7). Assays were run with multiple batches of PSVs and at least in duplicate. As PSV titers can vary, values indicated in each graph or table were obtained with the same batch of PSV to enable accurate comparison between the tested constructs.

**Epitope binning by bio-layer interferometry (BLI).** Nanobodies were binned into epitope specificities using an Octet RED384 system. 50 nM of $His_{10}$-tagged RBD protein antigen were captured using anti-Penta-HIS biosensors (18-5120, Molecular Devices). After RBD loading for 5 min, a saturating concentration of monoclonal antibodies (CR3022, CC6.30 or CC12.1), LM18-Fc or ACE2 (100 µg/mL) was added until saturation. Competing concentrations of nanobodies (25 µg/mL) were then added for 5 min to measure binding in the presence of saturating monoclonal antibody (or LM18 or ACE2). All incubation steps were performed in PBS with 0.1% TWEEN 20.

**Surface Plasmon Resonance (SPR) Methods.** SPR measurements were collected using a Biacore 8K instrument at 25°C. All experiments were carried out with a flow rate of 30 µL/min in a mobile phase of HBS-EP+ [0.01 M HEPES (pH 7.4), 0.15 M NaCl, 3 mM EDTA, 0.0005% (v/v) Surfactant P20]. Two chips were prepared in order to obtain data for the nanobodies (His-tagged) and nanobodies-Fc and $bsNb_4$-Igs. One, anti-Human IgG (Fc) antibody (Cytiva) was immobilized to a density of ~2000-4000 RU via standard NHS/EDC coupling to a Series S CM-3 (Cytiva) sensor chip; a reference surface was generated through the same method. Two, recombinant CoV-2-RBD was immobilized to a density of ~250 RU via standard NHS/EDC coupling to flow cell 2 of a Series S CM-5 (Cytiva) sensor chip; a reference surface was generated through activation/deactivation of flow cell 1.
For conventional kinetic/dose-response, $bsNb_4$-Igs were captured to ~50-100 RU via Fc-capture on the active flow cell prior to analyte injection. A concentration series of CoV-2-RBD or CoV-2-Spike were injected across the antibody and control surface for 2 min, followed by a 20 min dissociation phase using a multi-cycle method. Regeneration of the surface in between injections of antigen was achieved by two 120 s injections of 3 M $MgCl_2$. For conventional kinetics/dose-response of the nanobodies, a CoV-2-RBD sensor chip was prepared as stated above. A concentration series of each nanobody was injected over CoV-2-RBD and a control surface for 3 min, followed by a 15 min dissociation phase using a multi-cycle method. Regeneration of the surface in between injections of nanobody was achieved with a single, 60 s injection of 10 mM glycine (pH 1.5), 200 mM NaCl. Kinetic analysis of each reference subtracted injection series was performed using the BIAEvaluation software (Cytiva). Sensorgrams were fit to either a 1:1 (Langmuir) binding or heterogeneous ligand model.

**Crystallization and X-ray structure determination.** The engineered class 4 nanobodies, *i.e*. Nb-C4-225, Nb-C4-240, and Nb-C4-255, were mixed with equimolar SARS-CoV-2 RBD and CC12.1 Fab and incubated overnight at 4°C. 384 conditions of the JCSG Core Suite (Qiagen) were used for setting-up trays for crystal screening on the robotic CrystalMation system (Rigaku) at Scripps Research. Crystallization trials were set-up by the vapor diffusion method in sitting drops containing 0.1 µl of protein complex and 0.1 µl of reservoir solution. Crystals appeared on day 7, were harvested on day 12, pre-equilibrated in cryoprotectant containing 0-10% ethylene glycol, and then flash cooled and stored in liquid nitrogen until data collection. Diffraction quality crystals were obtained in solution containing 0.2 M di-ammonium citrate, 20% (w/v) polyethylene glycol 3350 for Nb-C4-225 complex, 0.16 M ammonium sulfate, 0.08 M sodium acetate pH 4.6, 20% (w/v) polyethylene glycol 4000, 20% (v/v) glycerol for Nb-C4-240 complex, and 0.2 M ammonium sulfate, 0.1 M sodium acetate pH 4.6, 25% (w/v) polyethylene glycol 4000 for Nb-C4-255 complex. Diffraction data were collected at cryogenic temperature (100 K) at the Stanford Synchrotron Radiation Lightsource (SSRL) on beamlines 12-1 and 12-2 for Nb-C4-225 and Nb-C4-240 complexes, and at beamline 23-ID-B of the Advanced Photon Source (APS) at Argonne National Laboratory for Nb-C4-255 complex. The X-ray data were processed with HKL2000 (8). The X-ray structures were solved by molecular replacement (MR) using PHASER (9) with MR models for the RBD and Nbs from 7KN5 (10) and for the Fab from 6XC3(11). Iterative model building and refinement were carried out in COOT (12) and PHENIX (12, 13), respectively.

**Cryo-electron microscopy.** Trimeric SARS-CoV-2 6P-Mut7 S protein was incubated with a threefold molar excess of LM18/Nb-C2-136 $bsNb_4$-Ig at room temperature for 100 minutes at a concentration of 0.85 mg/mL as determined by $A_{280}$. n-dodecyl-β-D-maltopyranoside (DDM) was added to a final concentration of 0.06 mM and the sample deposited on plasma-cleaned Quantifoil 1.2/1.3 300 mesh grids. A Thermo Fisher Vitrobot Mark IV set to 4°C, 100% humidity, 3 s wait time, and a 3 s blot time was used to vitrify samples in liquid ethane.
Data were collected using Leginon (14) on a Thermo Fisher Titan Krios operating at 300 keV and equipped with a Gatan K2 Summit direct electron detector. Movies were aligned and dose weighted using MotionCor2 (15). Aligned frames were imported into cryoSPARC v3.2 (15, 16) and the contrast transfer function (CTF) was estimated using GCTF (15-17). Particle picking was done by automated picking using templates created from an initial round of 2D classification, then extracted and subjected to multiple rounds of 2D classification for cleaning. An *ab initio* model was generated and several rounds of non-uniform refinement (18), CTF refinement and 3D variability analysis

were performed, resulting in a final global reconstruction (Fig. 5, Table S12). To further improve the resolution of the RBD and nanobody interactions, particles were exported to Relion 3.1 (19) and subjected to C3 symmetry expansion. A mask around a single RBD and nanobodies was created using University of California San Francisco Chimera (19, 20) and used for focused 3D classifications without alignment. During focused 3D refinement, a mask of the trimeric core and a single RBD with nanobodies was applied, and angular sampling was restricted to prevent rotation of one protomer onto another. A summary of data collection and processing statistics can be found in Table S12.

Initial models were generated by fitting coordinates from sAbPred (21) for the nanobodies and PDB 6VYB for the S protein into the focused refinement cryo-EM map. Several rounds of iterative manual and automated model building and relaxed refinement were performed using Coot 0.9.8 (22) and Phenix real_space_refine (23). Models were validated using EMRinger (24) and MolProbity (25). Kabat numbering was applied to the nanobody chains. Final refinement statistics and PDB/EMDB deposition codes can be found in Table S12.

**Modeling LM18/Nb-C2-136 bsNb$_4$-Ig**. Based on the approximate placement of $C_H1/C_L$, we used RosettaRemodel (26) to build the bsNb$_4$-Ig in three different steps. First, we modeled just the linkers (with the corresponding sequences) connecting $C_H1$ and Fc domains, requiring the C$\alpha$ and C$\beta$ atoms on the cysteine residues from the pairing heavy chain be 5.6 ± 1 Å and 4.0 ± 1 Å. Second, with “-bypass_fragments,” “-remodel:match_rt_limit 2,” and “-build_disulf” settings in RosettaRemodel, the geometries between the cysteine residues in models from step one were evaluated according to their closeness to known disulfide geometries in PDB. Third, for the structures with proper disulfide bond geometry, we modeled the linker region downstream from the disulfide and the Fc domains, requiring 5 sets of C$\alpha$ distance pairs derived from a native Fc dimer interface be satisfied. We generated 926 samples from step 1, of which 406 passed step 2, and we built 474 representative Fc models in step 3. After filtering out models with severe clashes based on Rosetta scores, the calculations resulted in 191 final models (Fig. S15). We computed the center of mass for the Fc domains and showed them in Figure S15B to illustrate the range of motion for the Fc domain with respect to $C_H1/C_L$ via flexible linkers.

1 10 20 30 40 50 60

EVQL**Q**ESGGGLVQPGGSLRLSCAASGFTFSSYAM**G**W**Y**RQAPGK**ER**EWVCAISGSGGSTYYA

70 80 90 101 110

DSVKGRFTCSRDNSKNTLYLQMNSL**KP**EDTAVYYCARXXXXXXXXXXFD**Y**WGQGT**Q**VTVSS

**Fig. S1.** Sequence of the $hV_{HH}323$ scaffold. The mutated position compared to $V_H3$ lineage are indicated in bold and blue. The positions mutated to Cys are indicated in yellow. The diversity of the CDRH3 is indicated in red. Residue numbers were assigned according to the Kabat numbering system.

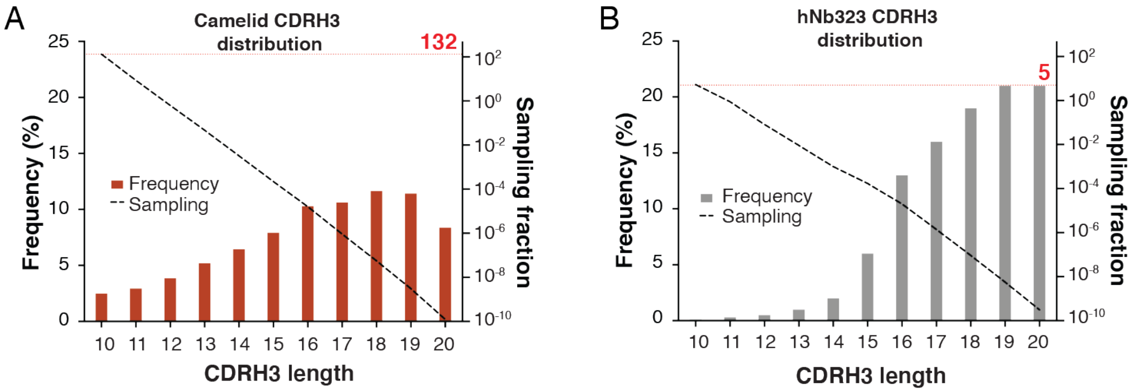


**Fig. S2.** Sampling fraction (indicated by the dotted line) of CDRH3s containing 10 to 20 residues depending on the frequency. A normal distribution of the CDRH3 length, such as for camelid repertoires, would result in an oversampling of the smaller CDRH3 by a factor of 132 (**A**). A left-skewed distribution prevents such oversampling and the smaller CDRH3 (10 residues) would be sampled by a factor of 5 (**B**).

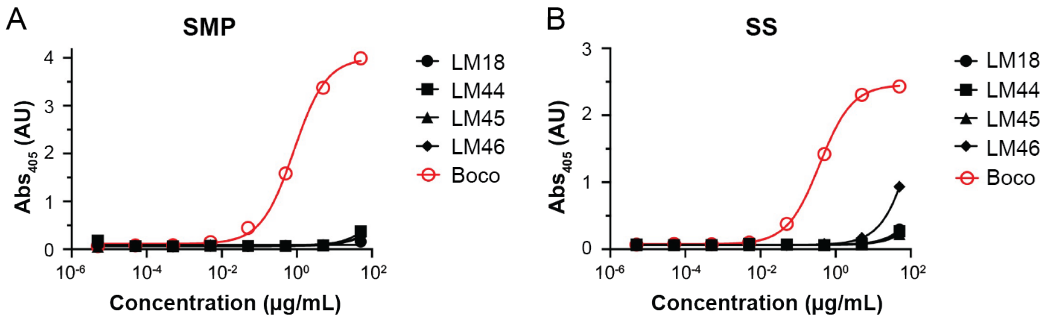


**Fig. S3.** Evaluation of the selected four nanobodies for polyreactivity. Fc-fused LM18, LM44, LM45 and LM46 were tested by ELISA for binding to the polyspecificity reagents: **A)** solubilized CHO-cell soluble membrane protein extracts (SMP) and **B)** single strand DNA (SS) (Sigma-Aldrich, D8899). *Bococizumab* ("Boco" CAS: 1407495-02-6) was used as a positive control to determine nonspecific binding. Error bars indicate the standard deviation of the mean.

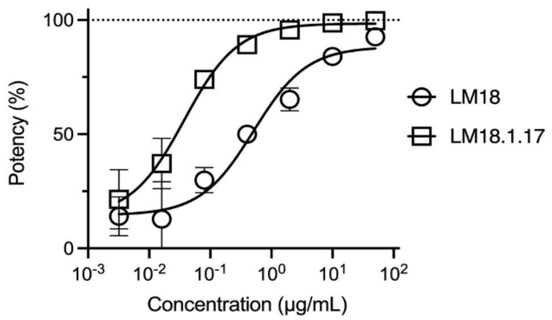


**Fig. S4.** Neutralization of SARS-CoV-1 PSV by LM18-Fc and LM18.1.17-Fc. The $IC_{50}$ values are 12 nM or 0.51 µg/mL and 0.9 nM or 0.037 µg/mL for LM18 and LM18.1.17, respectively. Assays were run in duplicate with a nanobody starting concentration of 50 µg/mL. Error bars indicate the standard deviation of the mean.

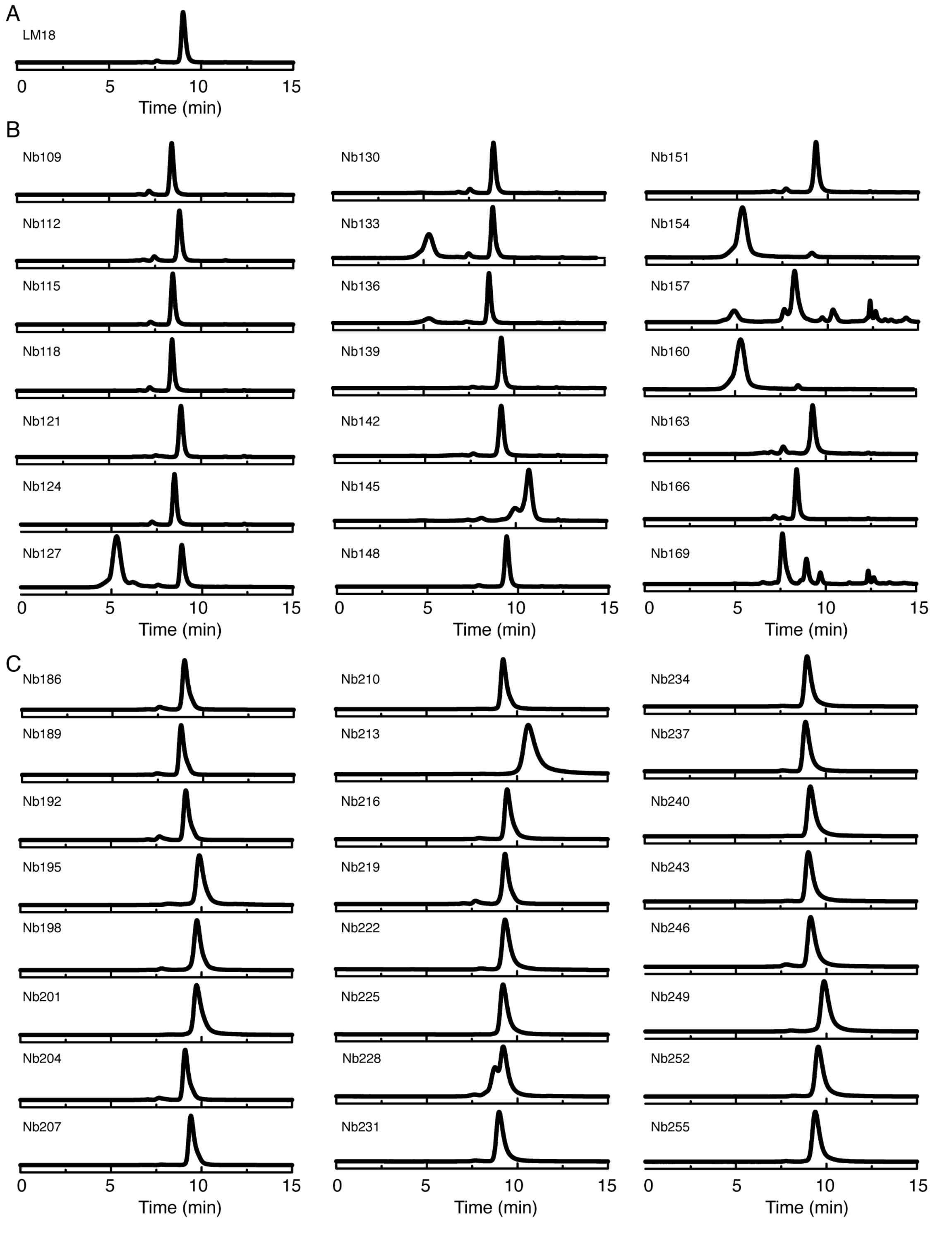


**Fig. S5.** Evaluation of nanobody-Fc by size-exclusion chromatography (SEC) using an Agilent 1260 Infinity II HPLC equipped with a TSKgel SuperSW mAb HR column (Tosoh, 7.8 mm I.D. x 30 cm, 4 µm) with a 1 mL/min flow rate and detection wavelength at 280 nm. An isocratic gradient of 100% PBS was used. **A)** LM18; **B)** Class 2 nanobodies and **C)** Class 4 nanobodies.

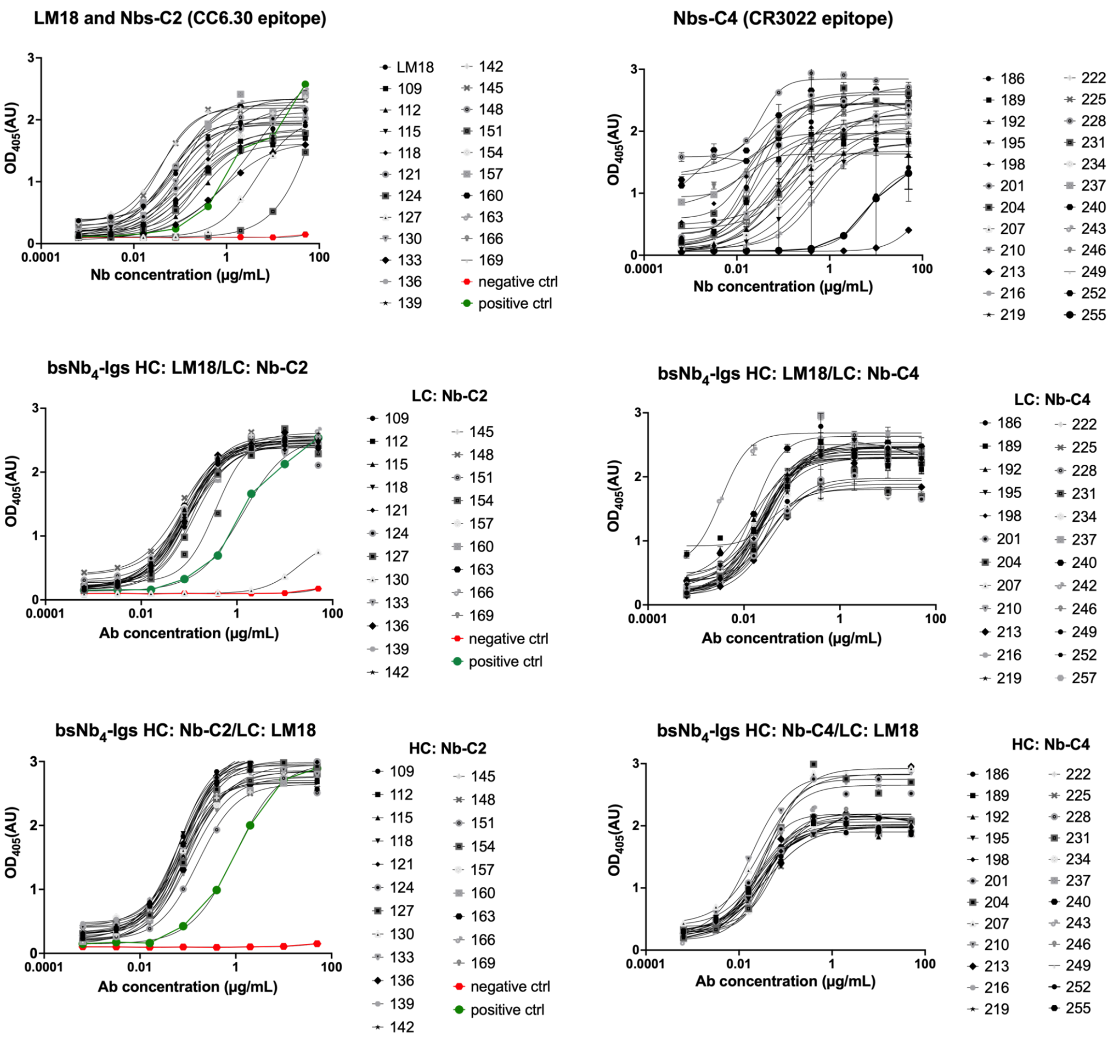


**Fig. S6.** Evaluation of nanobodies and bsNb$_4$-Igs for specific binding to Wuhan-1 SARS-CoV-2. Nanobodies-Fc (top two panels) and bsNb$_4$-Igs were tested by ELISA for binding to Wuhan-1 SARS-CoV-2 RBD. CC6.30 was used as a positive control (green) and a nanobody-Fc from our library not selected for RBD binding was used as a negative control (red). We tested the bsNb$_4$-Igs with LM18 either as a HC or LC and observed no notable difference for RBD binding, indicating that bsNb$_4$-Ig building block can be linked indifferently to the $C_H1$ or $C_L$. Error bars indicate the standard deviation of the mean.

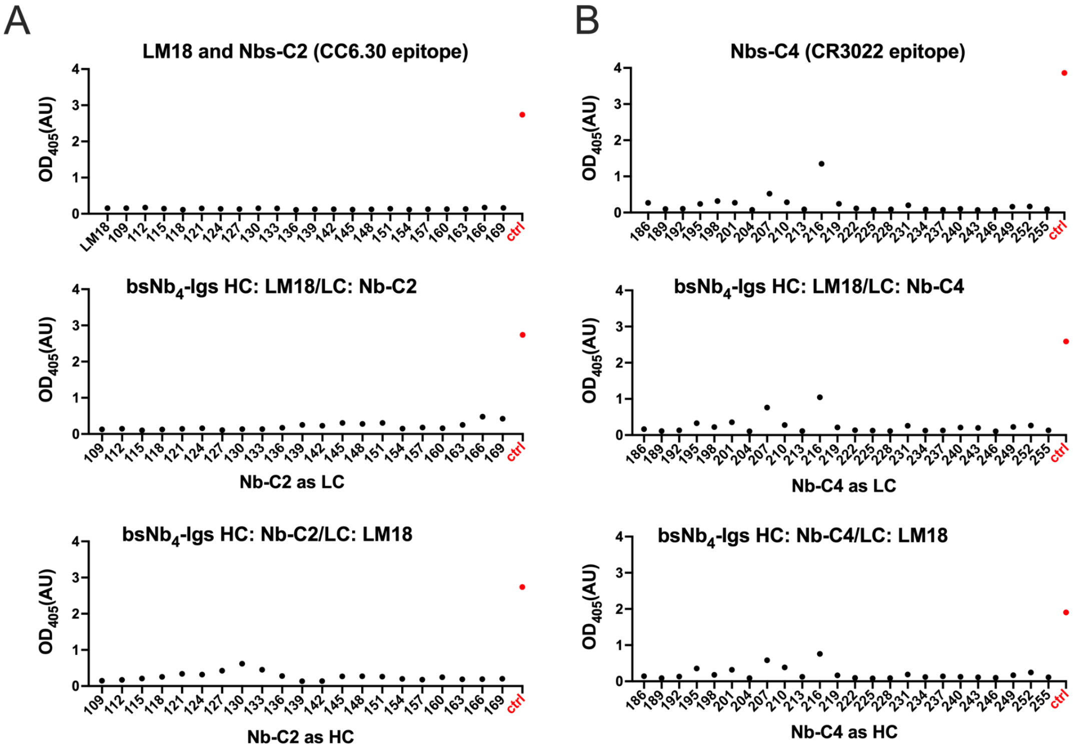


**Fig. S7.** Evaluation of SARS-CoV-2 nanobodies-Fc and IgG-like bsNb$_4$-Igs for polyreactivity. Nanobodies-C2 and nanobodies-C2-based bsNb$_4$-Igs **(A)** and nanobodies-C4 and nanobodies-C4-based bsNb$_4$-Igs **(B)** were tested by ELISA for binding to CHO-cell soluble membrane protein (SMP) extracts. *Bococizumab* (CAS: 1407495-02-6) was used as a control (ctrl) to determine nonspecific binding to SMP. We tested the bsNb$_4$-Igs with LM18 either as a HC or LC and observed no notable difference for nonspecific binding.

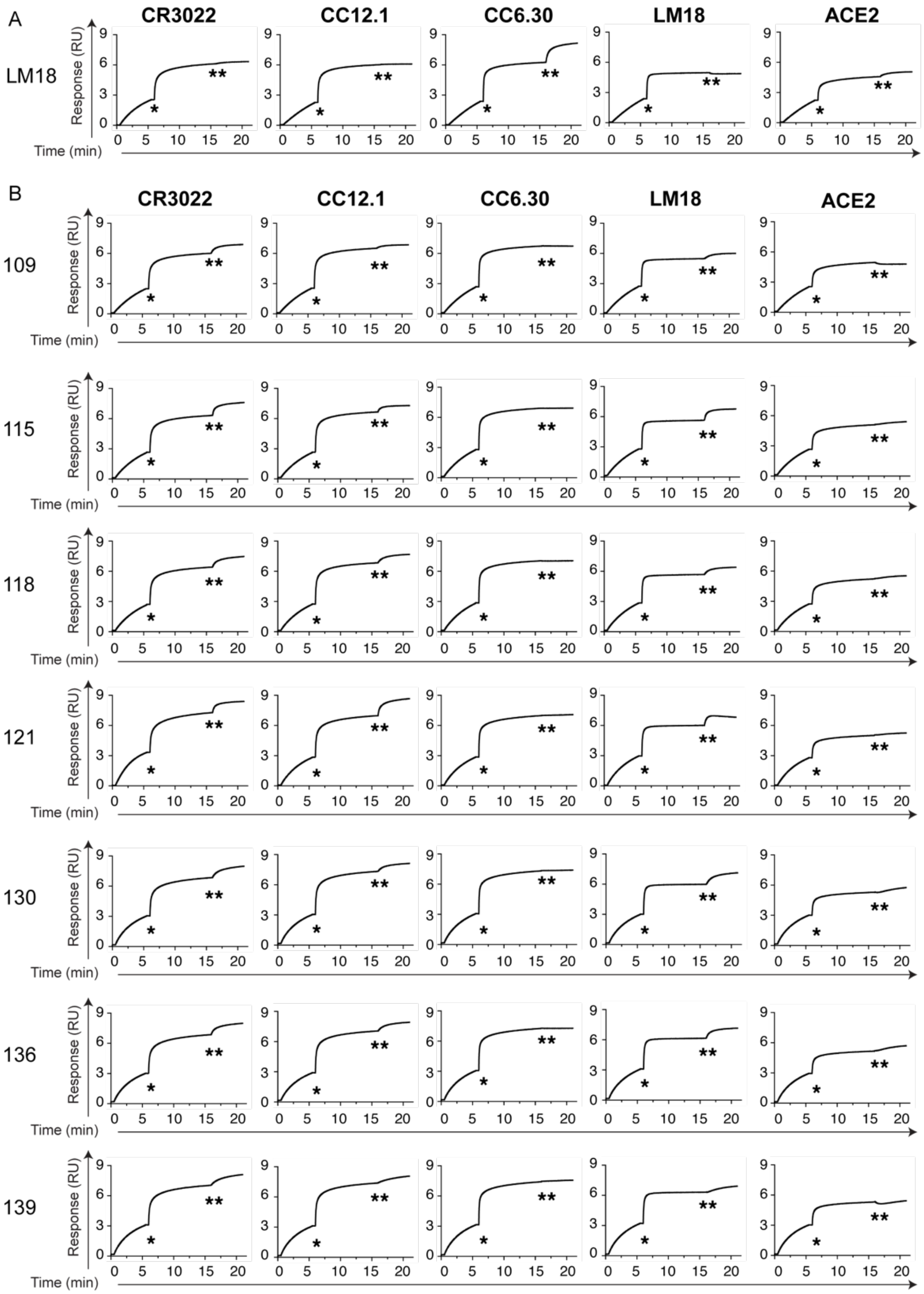
A
B
CR3022
CC12.1
CC6.30
LM18
ACE2
LM18
109
115
118
121
130
136
139
Response (RU)
Time (min)
*
**

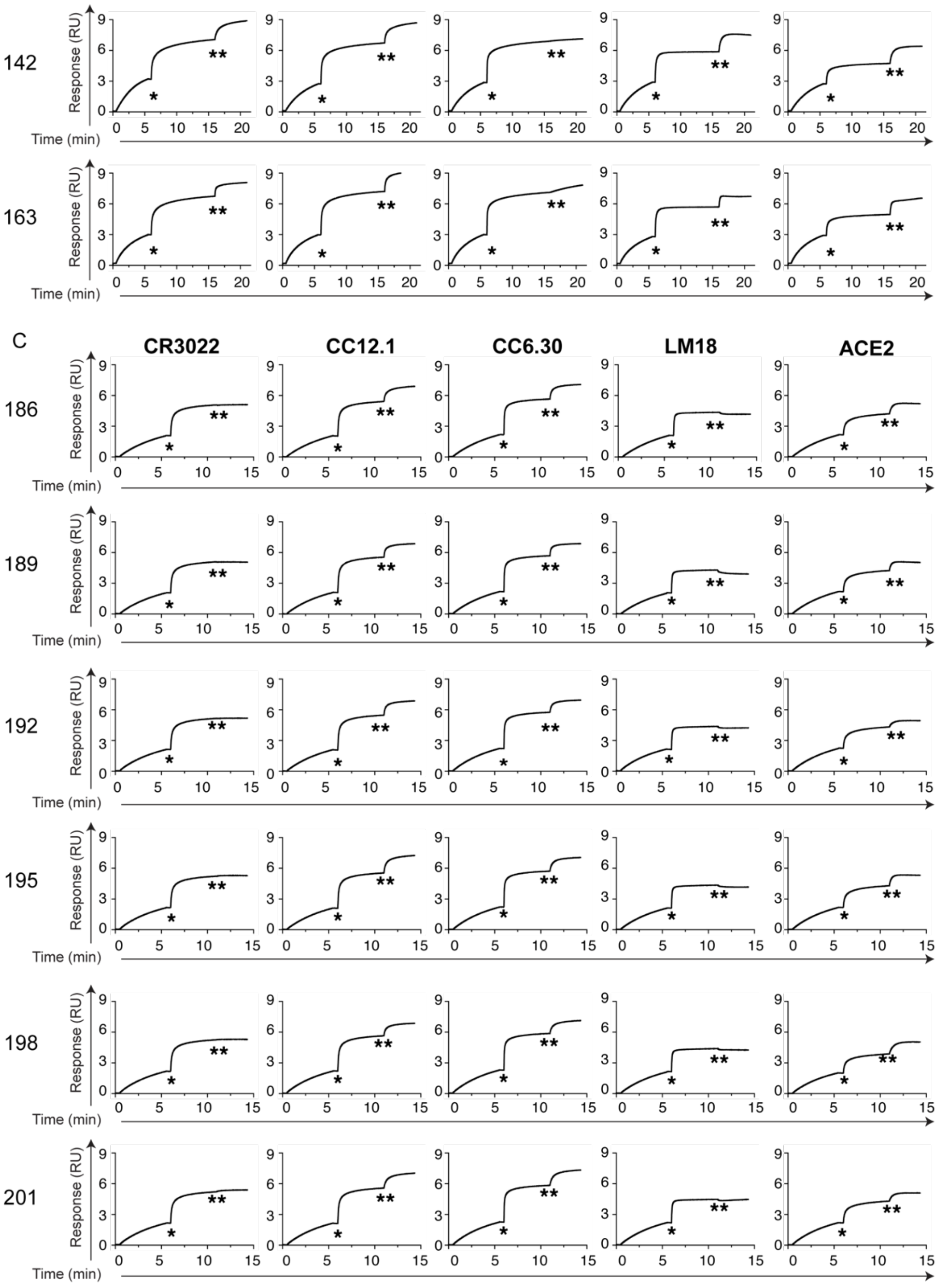
142
163
C
CR3022
CC12.1
CC6.30
LM18
ACE2
186
189
192
195
198
201
Response (RU)
Time (min)
*
**

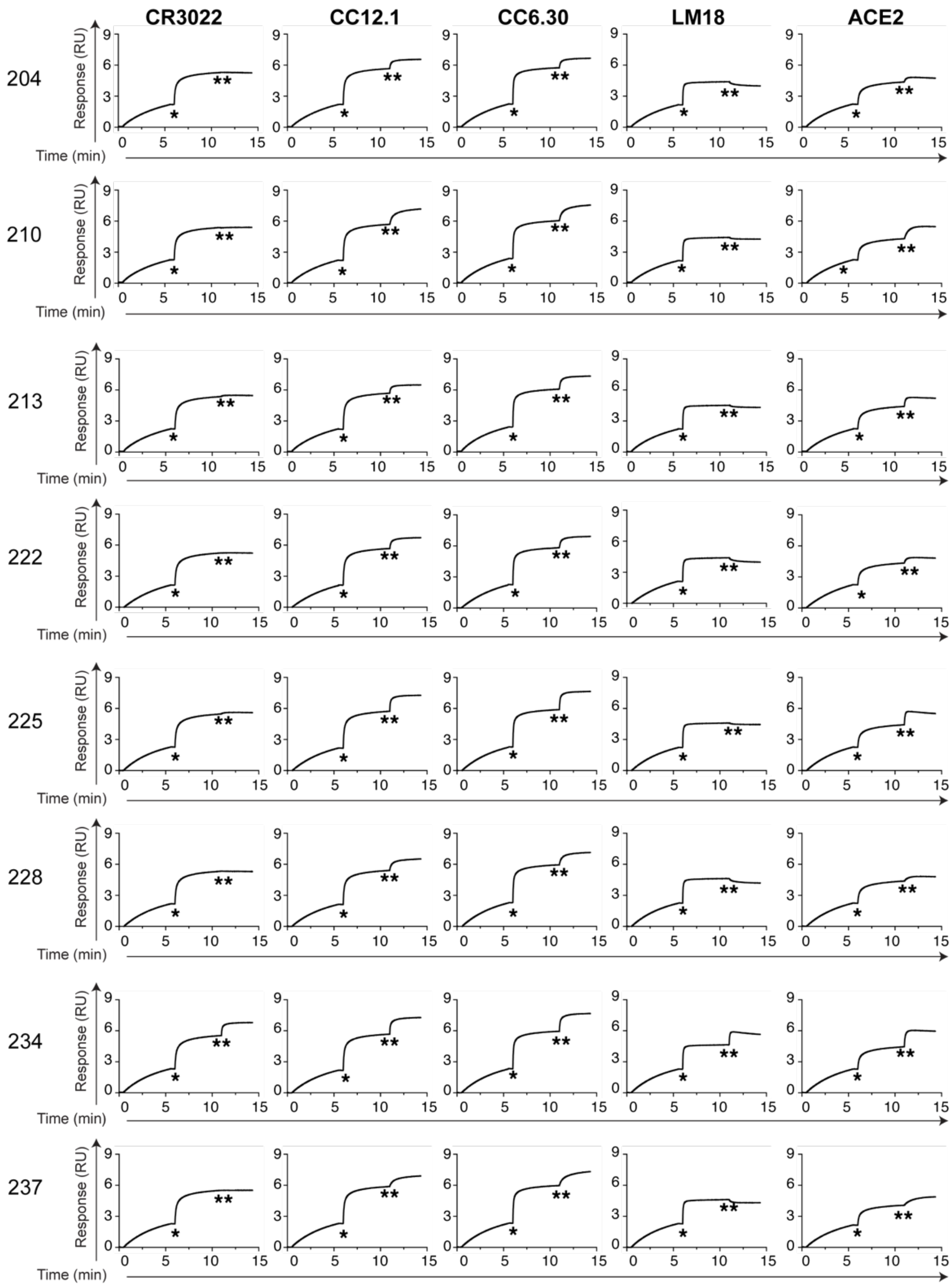
CR3022
CC12.1
CC6.30
LM18
ACE2
204
210
213
222
225
228
234
237
Response (RU)
Time (min)
*
**

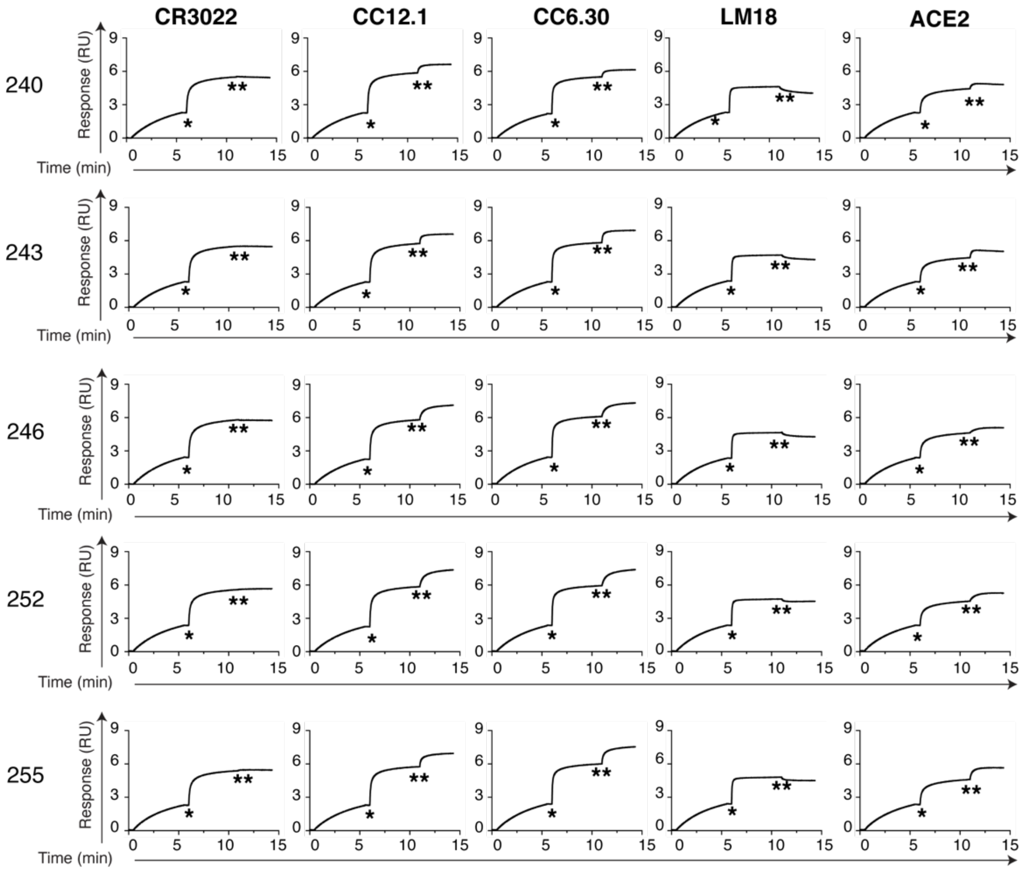


**Fig. S8.** Epitope binning of Fc-tagged nanobodies using an Octet RED384 platform. $His_{10}$-tagged RBD was captured using a Ni-NTA biosensor, and indicated monoclonal antibodies (CR3022, CC6.30, CC12.1) or nanobody (LM18), or ACE2 at a concentration of 100 μg/ml were first incubated (*), followed by an incubation with 25 μg/ml of competing nanobody (**). **A)** LM18; **B)** Class 2 nanobodies and **C)** Class 4 nanobodies. The tested nanobody # is indicated on the left of each graph.

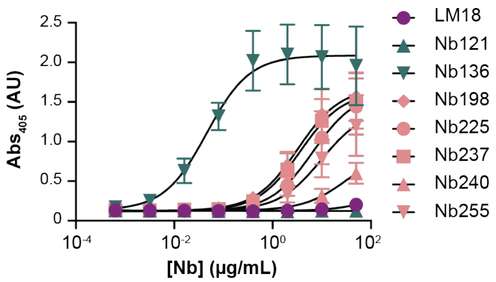


**Fig. S9.** Evaluation of nanobodies-Fc for specific binding to SARS-CoV-2 RBD *omicron* by ELISA. LM18 is shown in purple, nanobodies-C2 in teal and nanobodies-C4 in salmon. Assay was run in duplicate. Class 2 Nb-C2-136 and class 4 Nb-C4-198, Nb-C4-225, Nb-C4-237, Nb-C4-240 and Nb-C4-255 bind to RBD *omicron*, whereas LM18 and class 2 Nb-C2-121 binding is highly affected by the mutations present on this variant. Error bars indicate the standard deviation of the mean.

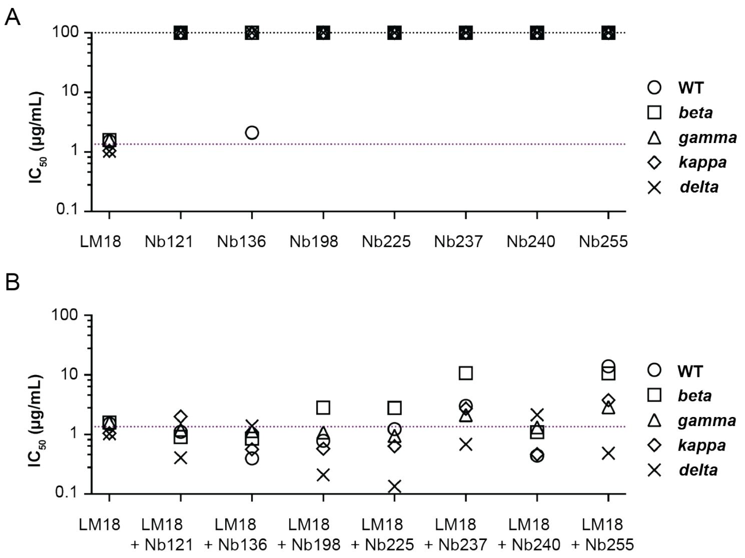


**Fig. S10.** Neutralization of PSVs variants by nanobodies and nanobody cocktails. **A**) Neutralization of PSVs carrying Wuhan-1 (wt) or mutated SARS-CoV-2 by Fc fused nanobodies. **B**) Neutralization of PSVs carrying Wuhan-1 (wt) or mutated SARS-CoV-2 by an equimolar ratio of by Fc fused nanobodies. The purple and black dotted lines illustrate the average of LM18-Fc $IC_{50}$ values and the absence of neutralization ($IC_{50}$ > 100 µg/mL), respectively.

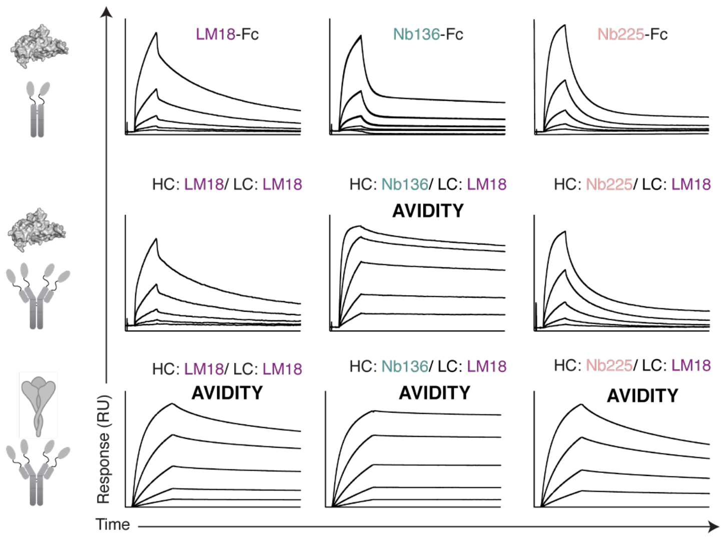


**Fig. S11.** SPR binding curves of LM18, one representative of the class 2 Nb (Nb-C2-136) and class 4 Nb (Nb-C4-225) for RBD and spike protein binding in different formats. In all cases, the Nb-Fc or Ab is immobilized by the Fc and the SARS-CoV-2 RBD or spike is flown as the analyte.

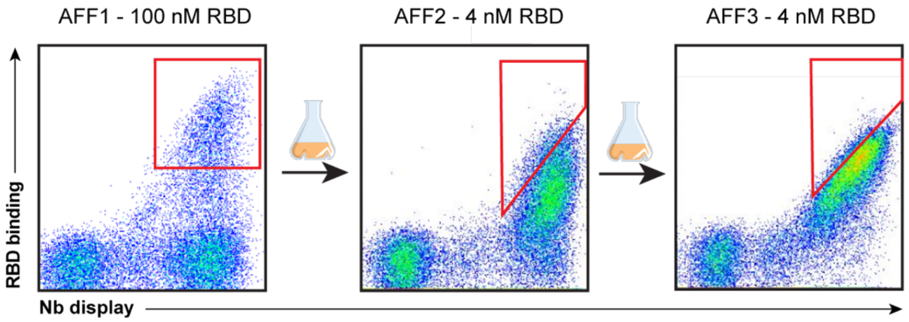


**Fig. S12.** Flow cytometry plots of the three affinity sorts for the affinity-maturation of LM18. The gates are indicated in red. Yeast display is shown on the x axis and RBD binding on the y axis. Of note, the gates in are representative, as the sorter does not record the actual sort gates used in the experiment.

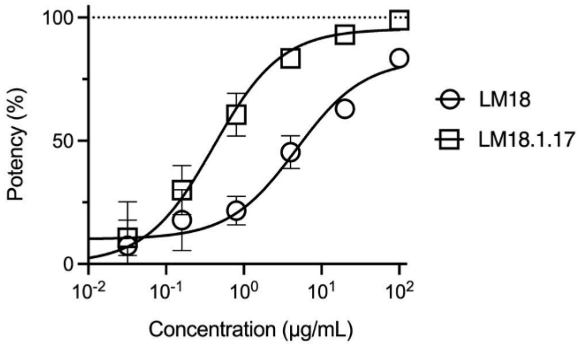


**Fig. S13.** Neutralization of Wuhan-1 SARS-CoV-2 PSV by LM18-Fc and matured LM18-Fc (LM18.1.17). Assays were run in triplicate with a nanobody starting concentration of 100 µg/mL. Error bars indicate the standard deviation of the mean.

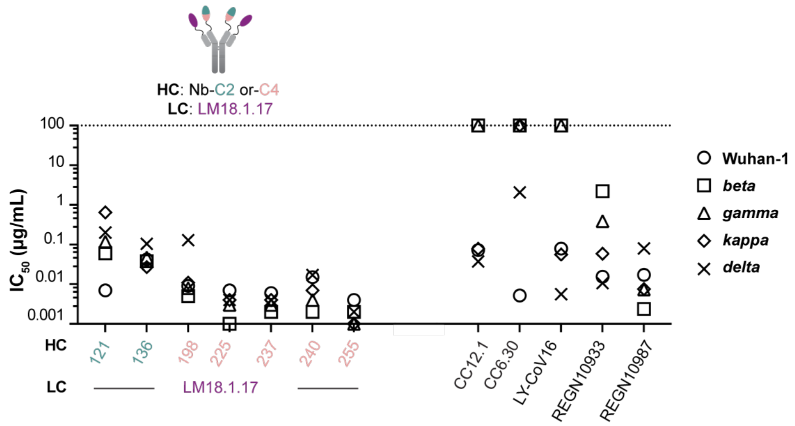


**Fig. S14** Neutralization of SARS-C0V-2 PSV variants by matured LM18 (LM18.1.17)- based bsNb4-Igs. $IC_{50}$ values of clinical antibody candidates (LY-CoV16, REGN10933 and REGN-10987), CC12.1 and CC6.30 from our previous study (27) were added for reference

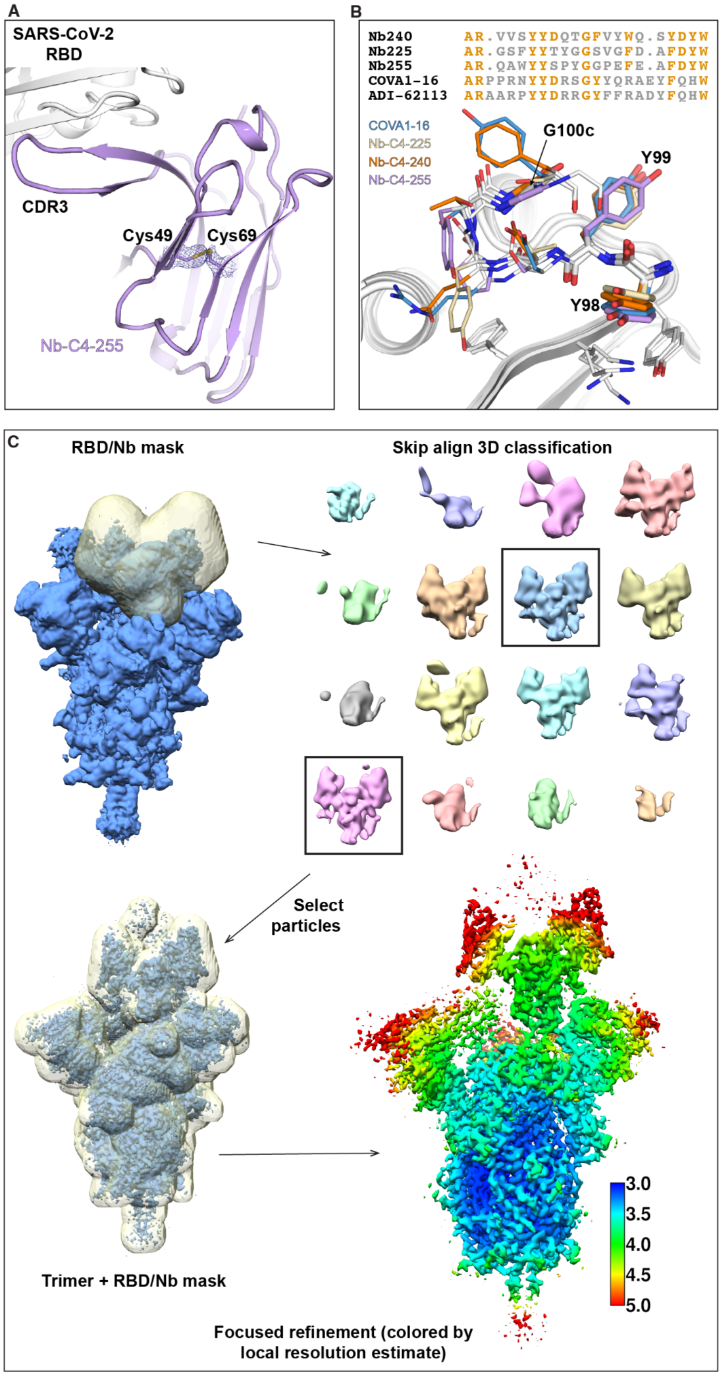


**Fig. S15.** Structural details elucidated by X-ray crystallography and cryo-EM. **A)** Disulfide bond in the reported engineered nanobodies. The blue mesh shows a 2mFc-DFo density map of the disulfide bond (shown as sticks) contoured at 1.0 σ. Nb-C4-255 is shown in light purple. SARS-CoV-2 RBD in gray. **B)** Structure-based sequence alignment of nanobody CDRH3s. CDRH3 of class 4 Nb-C4-225, Nb-C4-240, and Nb-C4-255 showed similar binding mode as YYDRxG antibodies, e.g. COVA1-16 (Fig. 5) and ADI-62113. The AA sequences constituting these CDRH3s are aligned based on structural superposition shown below. Yellow indicates conserved residues. CDRH3 tips of class 4 Nb-C4-225, Nb-C4-240, and Nb-C4-255 (sticks) are aligned to COVA1-16 based on SARS-CoV-2 RBD superimposition. Key residues in the CDRH3 and RBD are shown as sticks. **C)** Summary schematic of focused classification and refinement methods used to generate map for LM18/Nb-C2-136 bsNb$_4$-Ig + SARS-CoV-2 6Pmut7 S model building. See Methods for additional details.

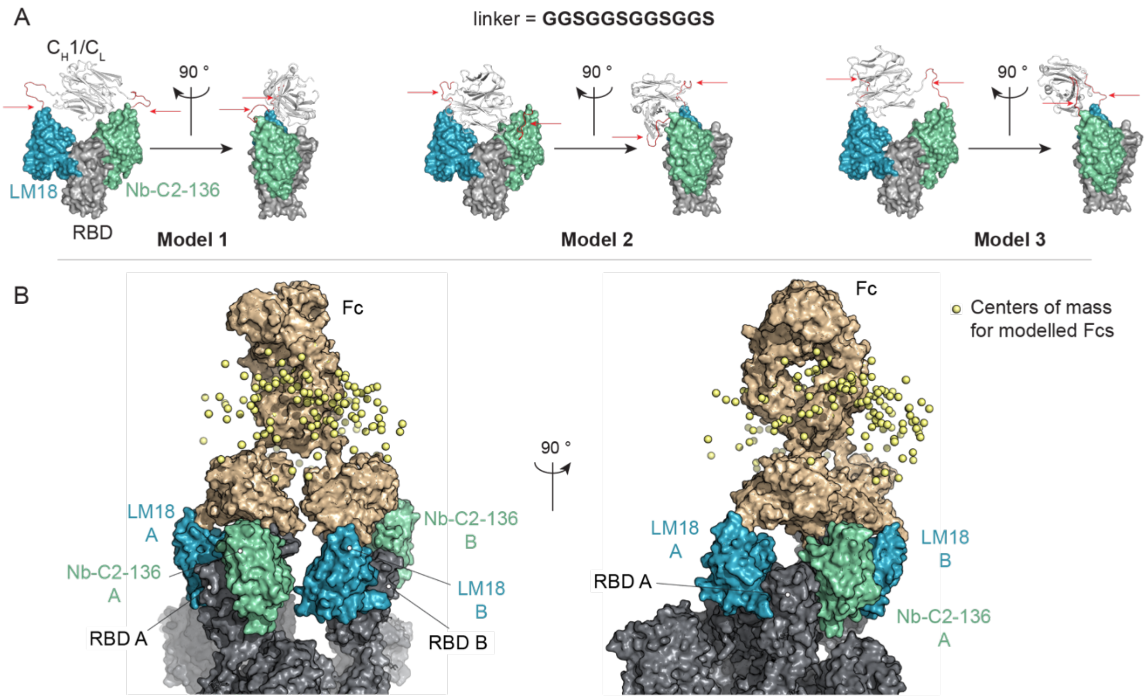


**Fig. S16.** Modeling of RBD-LM18/Nb-C2-136 Fab and bsNb$_4$-Ig format. **A)** Modeling of RBD-LM18/Nb-C2-136 Fab complex (based on the Cryo-EM reconstruction of LM18/Nb-C2-136 bsNb$_4$-Ig in complex with SARS-CoV-2 6Pmut7 S protein). The linkers are colored in red and indicated by the red arrows. The presence of a linker enables enough flexibility to the nanobody building blocks to bind RBD. **B)** Based on the $C_H1/C_L$ placement in Figure 5, a complete bsNb$_4$-Ig can be modeled to assess the plausibility of 1:1, bsNb$_4$-Ig to spike, binding. Fc is highly labile in the sample and has no discrete density in the EM reconstruction, the center-of-mass for ~200 alternative Fc locations are represented as yellow spheres here.

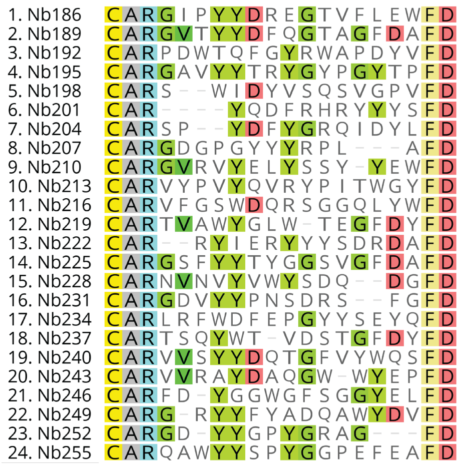


**Fig. S17.** Sequence alignment of CDRH3 of the selected 24 $hV_{HH}323$ nanobodies binned to compete with CR3022.

**Table S1.** $IC_{50}$ values and potency for neutralization of Wuhan-1 SARS-CoV-2 PSV by four nanobodies-Fc identified by Sanger sequencing. Assays were run in triplicate with a starting nanobody concentration of 100 µg/mL.

| | $IC_{50}$ ($\mu$g/mL) | $IC_{50}$ (nM) | % Neut | $R^2$ |
|---|---|---|---|---|
| **LM18** | **2.65** | 66.25 | **86** | **0.94** |
| LM44 | 70.93 | 1773.25 | 71 | 0.69 |
| LM45 | 4.16 | 104.03 | 46 | 0.71 |
| LM46 | 23.95 | 598.75 | 66 | 0.78 |

**Table S2.** NGS analysis and CDRH3 sequences from competitive sorts with CC12.1, CR3022, and CC6.30. In yellow are indicated the NGS counts for the 6 competition sorts, in grey the 4 sequences that correspond to nanobodies competing with the 3 epitopes that were not taken into account.

| # | All CDR3 | eCR30 22-C | eCR30 22-NC | ratio C/NC | CR302 2 compe te | CR302 2 nonco mpete | CC12. 1-C | CC12. 1-NC | ratio C/NC | CC12. 1 compe te | CC12. 1 nonco mpete | CC6.3 0-C | CC6.3 0-NC | ratio C/NC | CC6.3 0 compe te | CC6.3 0 nonco mpete | NC 3 Abs | | CR302 2 C/ CC12. 1 C | | CC6.3 0 C/ CC12. 1 C | | C 3 Abs | | CC6.3 0 C/ CR302 2 C | |
|---|---|---|---|---|---|---|---|---|---|---|---|---|---|---|---|---|---|---|---|---|---|---|---|---|---|---|
| | | | | | 43 | 34 | | | | 50 | 42 | | | | 69 | 35 | | 13 | | 8 | | 42 | | 4 | | 6 |
| 1 | CARAHGYSDYYSWHGVYTFDY | 9 | 384 | 0.023 | 0 | 1 | 269 | | 269 | 1 | 0 | 515 | 18 | 28.61 | 1 | 0 | 2 | 0 | 1 | 0 | 2 | 1 | 2 | 0 | 1 | 0 |
| 2 | CARALATSRHRHSTFDY | 34 | | 34 | 1 | 0 | 23 | | 23 | 1 | 0 | 9 | | 9 | 0 | 0 | 2 | 0 | 2 | 1 | 1 | 0 | 2 | 0 | 1 | 0 |
| 3 | CARARYSGSGDLPPPYFDY | 11 | 4778 | 0.002 | 0 | 1 | 14 | 18208 | 8E-04 | 0 | 1 | 7896 | 153 | 51.61 | 1 | 0 | 1 | 0 | 0 | 0 | 1 | 0 | 1 | 0 | 1 | 0 |
| 4 | CARAYDWWSVVPVYYGDFDY | 981 | 8104 | 0.121 | 0 | 0 | 8432 | 202 | 41.74 | 1 | 0 | 10979 | 262 | 41.9 | 1 | 0 | 2 | 0 | 1 | 0 | 2 | 1 | 2 | 0 | 1 | 0 |
| 5 | CARAYPPYVDPYYARVFDY | 560 | 4083 | 0.137 | 0 | 0 | 4727 | 19 | 248.8 | 1 | 0 | 6947 | 295 | 23.55 | 1 | 0 | 2 | 0 | 1 | 0 | 2 | 1 | 2 | 0 | 1 | 0 |
| 6 | CARAYYRLYGSDQRGFDY | | 93 | 0 | 0 | 1 | 85 | | 85 | 1 | 0 | 78 | | 78 | 1 | 0 | 2 | 0 | 1 | 0 | 2 | 1 | 2 | 0 | 1 | 0 |
| 7 | CARDRGLGDRPVGVYGPQFDY | 236 | 464 | 0.509 | 0 | 0 | 551 | 21 | 26.24 | 1 | 0 | 955 | 4 | 238.8 | 1 | 0 | 2 | 0 | 1 | 0 | 2 | 1 | 2 | 0 | 1 | 0 |
| 8 | CARDVPDAALYLEFDY | 1 | 1598 | 6E-04 | 0 | 1 | 166 | 42 | 3.952 | 0 | 0 | 526 | 90 | 5.844 | 0 | 0 | 0 | 1 | 0 | 0 | 0 | 0 | 0 | 0 | 0 | 0 |
| 9 | CAREQYGTVGYRRFDY | 37 | | 37 | 1 | 0 | 8 | | | 0 | 0 | 22 | | 22 | 1 | 0 | 2 | 0 | 1 | 0 | 1 | 0 | 2 | 0 | 2 | 1 |
| 10 | CARESSGYPQYASTPFDY | 20 | | 20 | 1 | 0 | 30 | | | 0 | 1 | 13 | | 13 | 1 | 0 | 2 | 0 | 1 | 0 | 1 | 0 | 2 | 0 | 2 | 1 |
| 11 | CARFDYGGWGFSGGYELFDY | 1254 | | 1254 | 1 | 0 | 10 | 342 | 0.029 | 0 | 1 | 28 | 868 | 0.032 | 0 | 1 | 1 | 0 | 1 | 0 | 0 | 0 | 1 | 0 | 1 | 0 |
| 12 | CARFGTFDNQYWYGRAALFDY | 505 | 984 | 0.513 | 0 | 0 | 1356 | 35 | 38.74 | 1 | 0 | 2374 | 79 | 30.05 | 1 | 0 | 2 | 0 | 1 | 0 | 2 | 1 | 2 | 0 | 1 | 0 |
| 13 | CARFPLDYYPYGRRYPGFDY | 4920 | 1113 | 4.42 | 0 | 0 | 5692 | 19 | 299.6 | 1 | 0 | 8727 | 153 | 57.04 | 1 | 0 | 2 | 0 | 1 | 0 | 2 | 1 | 2 | 0 | 1 | 0 |
| 14 | CARFRWYDGRDFKVSAFFDY | 5 | 720 | 0.007 | 0 | 1 | | 2218 | 0 | 0 | 1 | 890 | 12 | 74.17 | 1 | 0 | 1 | 0 | 0 | 0 | 1 | 0 | 1 | 0 | 1 | 0 |
| 15 | CARFYWGVYAGGPGYPFDY | 67 | 1509 | 0.044 | 0 | 1 | 1256 | 52 | 24.15 | 1 | 0 | 2193 | 47 | 46.66 | 1 | 0 | 2 | 0 | 1 | 0 | 2 | 1 | 2 | 0 | 1 | 0 |
| 16 | CARGARGLVYFWRTYGQPFDY | 91 | 117 | 0.778 | 0 | 0 | 145 | 2 | 72.5 | 1 | 0 | 313 | 3 | 104.3 | 1 | 0 | 2 | 0 | 1 | 0 | 2 | 1 | 2 | 0 | 1 | 0 |
| 17 | CARGAVYYTRYGYPGYTPFDY | 1304 | | 1304 | 1 | 0 | 22 | 855 | 0.026 | 0 | 1 | 31 | 1660 | 0.019 | 0 | 1 | 1 | 0 | 1 | 0 | 0 | 0 | 1 | 0 | 1 | 0 |
| 18 | CARGDGPGYYYRPLAFDY | 1433 | 3 | 477.7 | 1 | 0 | 341 | 20 | 17.05 | 1 | 0 | 479 | 99 | 4.838 | 0 | 0 | 2 | 0 | 2 | 1 | 1 | 0 | 2 | 0 | 1 | 0 |
| 19 | CARGDSQWPLWVKSYFDY | 25 | 252 | 0.099 | 0 | 1 | 18 | 428 | 0.042 | 0 | 1 | 58 | 660 | 0.088 | 0 | 1 | 0 | 1 | 0 | 0 | 0 | 0 | 0 | 0 | 0 | 0 |
| 20 | CARGDVYYPNSDRSFGFDY | 6305 | 31 | 203.4 | 1 | 0 | | 6159 | 0 | 0 | 1 | 8 | 8978 | 9E-04 | 0 | 1 | 1 | 0 | 1 | 0 | 0 | 0 | 1 | 0 | 1 | 0 |
| *21* | *CARGDYFLHWDGQYYFDY* | *61* | | *61* | | | *93* | | *93* | | | *71* | | *71* | | | | | | | | | *0* | *1* | | |
| 22 | CARGDYYGPYGRAGFDY | 25594 | 19 | 1347 | 1 | 0 | 793 | 7020 | 0.113 | 0 | 0 | 901 | 23870 | 0.038 | 0 | 1 | 1 | 0 | 1 | 0 | 0 | 0 | 1 | 0 | 1 | 0 |
| *23* | *CARGEYPLRPPGYPLYDVFDY* | *370* | | *370* | | | *51* | | *51* | | | *97* | *1* | *97* | | | | | | | | | *0* | *1* | | |
| 24 | CARGIPYYDREGTVFLEWFDY | 5447 | 3 | 1816 | 1 | 0 | 10 | 4446 | 0.002 | 0 | 1 | 13 | 7853 | 0.002 | 0 | 1 | 1 | 0 | 1 | 0 | 0 | 0 | 1 | 0 | 1 | 0 |
| 25 | CARGPDPPYYPVAVWLYAFDY | 3160 | 4649 | 0.68 | 0 | 0 | 7360 | 267 | 27.57 | 1 | 0 | 11121 | 309 | 35.99 | 1 | 0 | 2 | 0 | 1 | 0 | 2 | 1 | 2 | 0 | 1 | 0 |
| 26 | CARGPHYTERYIGDSYFDY | 1127 | 1457 | 0.774 | 0 | 0 | 3139 | 12 | 261.6 | 1 | 0 | 4949 | 101 | 49 | 1 | 0 | 2 | 0 | 1 | 0 | 2 | 1 | 2 | 0 | 1 | 0 |
| 27 | CARGPPWYAYVSWRDYDYFDY | 15 | 45 | 0.333 | 0 | 0 | 58 | | 58 | 1 | 0 | 46 | | 46 | 1 | 0 | 2 | 0 | 1 | 0 | 2 | 1 | 2 | 0 | 1 | 0 |
| 28 | CARGRYYFYADQAWYDVFDY | 226 | 13 | 17.38 | 1 | 0 | | 256 | 0 | 0 | 1 | 6 | 426 | 0.014 | 0 | 1 | 1 | 0 | 1 | 0 | 0 | 0 | 1 | 0 | 1 | 0 |
| 29 | CARGSFYYTYGGSVGFDAFDY | 18362 | 28 | 655.8 | 1 | 0 | 814 | 7384 | 0.11 | 0 | 0 | 40 | 23665 | 0.002 | 0 | 1 | 1 | 0 | 1 | 0 | 0 | 0 | 1 | 0 | 1 | 0 |
| 30 | CARGVPWPLYYIGDDTFDY | 109 | 1099 | 0.099 | 0 | 1 | 842 | 8 | 105.3 | 1 | 0 | 1446 | 61 | 23.7 | 1 | 0 | 2 | 0 | 1 | 0 | 2 | 1 | 2 | 0 | 1 | 0 |
| 31 | CARGVRVYELYSSYYEWFDY | 76 | | 76 | 1 | 0 | | 82 | 0 | 0 | 1 | | 79 | 0 | 0 | 1 | 1 | 0 | 1 | 0 | 0 | 0 | 1 | 0 | 1 | 0 |
| 32 | CARGVTYYDFQGTAGFDAFDY | 4536 | 6 | 756 | 1 | 0 | 14 | 4162 | 0.003 | 0 | 1 | 4 | 6017 | 7E-04 | 0 | 1 | 1 | 0 | 1 | 0 | 0 | 0 | 1 | 0 | 1 | 0 |
| 33 | CARGVYSVIYGERVGGPFDY | 824 | 1035 | 0.796 | 0 | 0 | 1294 | 57 | 22.7 | 1 | 0 | 2326 | 55 | 42.29 | 1 | 0 | 2 | 0 | 1 | 0 | 2 | 1 | 2 | 0 | 1 | 0 |
| 34 | CARGVYSVSSDERTPDDYFDY | | 8 | 0 | 0 | 0 | | 15 | 0 | 0 | 1 | | 27 | 0 | 0 | 1 | 0 | 1 | 0 | 0 | 0 | 0 | 0 | 0 | 0 | 0 |
| 35 | CARGWLWRYAQGLVSDYYFDY | 46 | 13 | 3.538 | 0 | 0 | | 30 | 0 | 0 | 1 | | 109 | 0 | 0 | 1 | 0 | 1 | 0 | 0 | 0 | 0 | 0 | 0 | 0 | 0 |
| 36 | CARGYIERYYYSDRDAFDY | 23 | | 23 | 1 | 0 | | 9 | 0 | 0 | | | 21 | 0 | 0 | 1 | 1 | 0 | 1 | 0 | 0 | 0 | 1 | 0 | 1 | 0 |
| 37 | CARHALPDVWYWGSRVFDY | | 219 | 0 | 0 | 1 | 63 | | 63 | 1 | 0 | 154 | 1 | 154 | 1 | 0 | 2 | 0 | 1 | 0 | 2 | 1 | 2 | 0 | 1 | 0 |
| 38 | CARHASFYGGDIERYFDY | | 1162 | 0 | 0 | 1 | 663 | | 663 | 1 | 0 | 1623 | 56 | 28.98 | 1 | 0 | 2 | 0 | 1 | 0 | 2 | 1 | 2 | 0 | 1 | 0 |
| 39 | CARHAYPLRVWPLTFDY | 11 | 408 | 0.027 | 0 | 1 | 129 | | 129 | 1 | 0 | 417 | 8 | 52.13 | 1 | 0 | 2 | 0 | 1 | 0 | 2 | 1 | 2 | 0 | 1 | 0 |
| 40 | CARHGYTLSDTYYYRYYQFDY | 7 | 130 | 0.054 | 0 | 1 | 134 | | 134 | 1 | 0 | 273 | 2 | 136.5 | 1 | 0 | 2 | 0 | 1 | 0 | 2 | 1 | 2 | 0 | 1 | 0 |
| 41 | CARHTYPLRVWPLNFDY | 401 | 3732 | 0.107 | 0 | 0 | 2572 | 120 | 21.43 | 1 | 0 | 4804 | 130 | 36.95 | 1 | 0 | 2 | 0 | 1 | 0 | 2 | 1 | 2 | 0 | 1 | 0 |

| | | | | | | | | | | | | | | | | | | | | | | | | | | |
|---|---|---|---|---|---|---|---|---|---|---|---|---|---|---|---|---|---|---|---|---|---|---|---|---|---|---|
| 42 | CARHYRVYGGSDYASFDY | 18 | 1532 | 0.012 | 0 | 1 | 1435 | | 1435 | 1 | 0 | 2710 | 26 | 104.2 | 1 | 0 | 2 | 0 | 1 | 0 | 2 | 1 | 2 | 0 | 1 | 0 |
| 43 | CARILDDERWAWRFYGFDY | 1389 | 2639 | 0.526 | 0 | 0 | 109 | 10010 | 0.011 | 0 | 1 | 5977 | 123 | 48.59 | 1 | 0 | 1 | 0 | 0 | 0 | 1 | 0 | 1 | 0 | 1 | 0 |
| 44 | CARIYWYPGRQPGGFDY | 573 | 2342 | 0.245 | 0 | 0 | 1860 | 88 | 21.14 | 1 | 0 | 3372 | 88 | 38.32 | 1 | 0 | 2 | 0 | 1 | 0 | 2 | 1 | 2 | 0 | 1 | 0 |
| 45 | CARLADWTWRFGGFDY | 1622 | 404 | 4.015 | 0 | 0 | 708 | 145 | 4.883 | 0 | 0 | 1827 | 43 | 42.49 | 1 | 0 | 1 | 0 | 0 | 0 | 1 | 0 | 1 | 0 | 1 | 0 |
| 46 | CARLLPIPYEYDYRDWFDY | 145 | 104 | 1.394 | 0 | 0 | 27 | 313 | 0.086 | 0 | 1 | 17 | 1131 | 0.015 | 0 | 1 | 0 | 1 | 0 | 0 | 0 | 0 | 0 | 0 | 0 | 0 |
| 47 | CARLPTRGYSENSGLWYFDY | 2862 | 12516 | 0.229 | 0 | 0 | 15416 | 276 | 55.86 | 1 | 0 | 23163 | 365 | 63.46 | 1 | 0 | 2 | 0 | 1 | 0 | 2 | 1 | 2 | 0 | 1 | 0 |
| 48 | CARLQRYFTDKGDPIIYFDY | 60 | 35 | 1.714 | 0 | 0 | 3 | 141 | 0.021 | 0 | 1 | 7 | 427 | 0.016 | 0 | 1 | 0 | 1 | 0 | 0 | 0 | 0 | 0 | 0 | 0 | 0 |
| 49 | CARLRFWDFEPGYYSEYQFDY | 2611 | 5 | 522.2 | 1 | 0 | 18 | 2410 | 0.007 | 0 | 1 | 21 | 2866 | 0.007 | 0 | 1 | 1 | 0 | 1 | 0 | 0 | 0 | 1 | 0 | 1 | 0 |
| 50 | CARLRQTEQGIVFDY | 6169 | 2756 | 2.238 | 0 | 0 | 1334 | 9384 | 0.142 | 0 | 0 | 2395 | 18424 | 0.13 | 0 | 0 | 0 | 1 | 0 | 0 | 0 | 0 | 0 | 0 | 0 | 0 |
| 51 | CARLWYYVPTGADVTVSFDY | 382 | 1 | 382 | 1 | 0 | 61 | | 61 | 1 | 0 | 145 | 22 | 6.591 | 0 | 0 | 2 | 0 | 2 | 1 | 1 | 0 | 2 | 0 | 1 | 0 |
| 52 | CARLYPGRHFISFDY | 34 | | 34 | 1 | 0 | 9 | | 9 | 0 | 0 | | 47 | 0 | 0 | 1 | 1 | 0 | 1 | 0 | 0 | 0 | 1 | 0 | 1 | 0 |
| 53 | CARLYWGPLGYRSAFDY | 23 | 19 | 1.211 | 0 | 0 | 12 | 62 | 0.194 | 0 | 0 | 82 | | 82 | 1 | 0 | 1 | 0 | 0 | 0 | 1 | 0 | 1 | 0 | 1 | 0 |
| 54 | CARNIRGLYGSYPESVFDY | 562 | 3944 | 0.142 | 0 | 0 | 4305 | 79 | 54.49 | 1 | 0 | 6901 | 107 | 64.5 | 1 | 0 | 2 | 0 | 1 | 0 | 2 | 1 | 2 | 0 | 1 | 0 |
| 55 | CARNVNVYVWYSDQDGFDY | 7376 | 8 | 922 | 1 | 0 | 21 | 4625 | 0.005 | 0 | 1 | 194 | 8239 | 0.024 | 0 | 1 | 1 | 0 | 1 | 0 | 0 | 0 | 1 | 0 | 1 | 0 |
| 56 | CARNYPGVFGLTPDGFDY | 81 | 81 | 1 | 0 | 0 | 205 | | 205 | 1 | 0 | 268 | 20 | 13.4 | 1 | 0 | 2 | 0 | 1 | 0 | 2 | 1 | 2 | 0 | 1 | 0 |
| 57 | CARPDWTQFGYRWAPDYVFDY | 4321 | 6 | 720.2 | 1 | 0 | 30 | 1310 | 0.023 | 0 | 1 | 219 | 2290 | 0.096 | 0 | 1 | 1 | 0 | 1 | 0 | 0 | 0 | 1 | 0 | 1 | 0 |
| 58 | CARPDYHRYRSYPGVFDY | 379 | 37 | 10.24 | 1 | 0 | 95 | 149 | 0.638 | 0 | 0 | 298 | 3 | 99.33 | 1 | 0 | 2 | 0 | 1 | 0 | 1 | 0 | 2 | 0 | 2 | 1 |
| 59 | CARPEGVVLGYPASIIFDY | 28 | | 28 | 1 | 0 | | | | 0 | | 3 | | 3 | 0 | 0 | 1 | 0 | 1 | 0 | 0 | 0 | 1 | 0 | 1 | 0 |
| 60 | CARPVYQHPPVYSLHFDY | | 506 | 0 | 0 | 1 | 214 | | 214 | 1 | 0 | 567 | 29 | 19.55 | 1 | 0 | 2 | 0 | 1 | 0 | 2 | 1 | 2 | 0 | 1 | 0 |
| 61 | CARQAWYYSPYGGPEFEAFDY | 16341 | 35 | 466.9 | 1 | 0 | 961 | 6502 | 0.148 | 0 | 0 | 63 | 23520 | 0.003 | 0 | 1 | 1 | 0 | 1 | 0 | 0 | 0 | 1 | 0 | 1 | 0 |
| 62 | CARQDRYGVWPGPRQYFDY | | 681 | 0 | 0 | 1 | | 1669 | 0 | 0 | 1 | 339 | 90 | 3.767 | 0 | 0 | 0 | 1 | 0 | 0 | 0 | 0 | 0 | 0 | 0 | 0 |
| 63 | CARQIALYDRYLHEDTLFDY | 52 | | 52 | 1 | 0 | 3 | | 3 | 0 | 0 | 11 | | 11 | 1 | 0 | 2 | 0 | 1 | 0 | 1 | 0 | 2 | 0 | 2 | 1 |
| 64 | CARRASISGYPAWSYFDY | 596 | | 596 | 1 | 0 | 160 | | 160 | 1 | 0 | 213 | 39 | 5.462 | 0 | 0 | 2 | 0 | 2 | 1 | 1 | 0 | 2 | 0 | 1 | 0 |
| 65 | CARRAYAIARATDIYYFDY | 41 | 173 | 0.237 | 0 | 0 | | 549 | 0 | 0 | 1 | 222 | 3 | 74 | 1 | 0 | 1 | 0 | 0 | 0 | 1 | 0 | 1 | 0 | 1 | 0 |
| 66 | CARRPLSLRRYTDIYPFDY | 749 | 144 | 5.201 | 0 | 0 | 414 | 9 | 46 | 1 | 0 | 802 | 12 | 66.83 | 1 | 0 | 2 | 0 | 1 | 0 | 2 | 1 | 2 | 0 | 1 | 0 |
| 67 | CARRRLASLSDSYDLVFDY | 46 | 46 | 1 | 0 | 0 | 9 | 187 | 0.048 | 0 | 1 | | 250 | 0 | 0 | 1 | 0 | 1 | 0 | 0 | 0 | 0 | 0 | 0 | 0 | 0 |
| 68 | CARRRYVSQWSPGQGPSFDY | 633 | 13269 | 0.048 | 0 | 1 | 54 | 53804 | 0.001 | 0 | 1 | 84 | 69784 | 0.001 | 0 | 1 | 0 | 1 | 0 | 0 | 0 | 0 | 0 | 0 | 0 | 0 |
| 69 | CARRWGVDASYVPPFDY | 16 | | 16 | 1 | 0 | 5 | | 5 | 0 | 0 | 23 | | 23 | 1 | 0 | 2 | 0 | 1 | 0 | 1 | 0 | 2 | 0 | 2 | 1 |
| 70 | CARRYIERYYYSDRDAFDY | 18772 | 6 | 3129 | 1 | 0 | 108 | 12553 | 0.009 | 0 | 1 | 476 | 17698 | 0.027 | 0 | 1 | 1 | 0 | 1 | 0 | 0 | 0 | 1 | 0 | 1 | 0 |
| 71 | CARSAWVIDYYPSPYFDY | 21 | 681 | 0.031 | 0 | 1 | | 1579 | 0 | 0 | 1 | 1044 | 20 | 52.2 | 1 | 0 | 1 | 0 | 0 | 0 | 1 | 0 | 1 | 0 | 1 | 0 |
| 72 | CARSEPVFYTTQLYFWAPFDY | | 263 | 0 | 0 | 1 | 68 | 39 | 1.744 | 0 | 0 | 275 | 28 | 9.821 | 0 | 0 | 0 | 1 | 0 | 0 | 0 | 0 | 0 | 0 | 0 | 0 |
| 73 | CARSGYGSTVWGDQYFDY | 2974 | 5764 | 0.516 | 0 | 0 | 8048 | 93 | 86.54 | 1 | 0 | 12362 | 226 | 54.7 | 1 | 0 | 2 | 0 | 1 | 0 | 2 | 1 | 2 | 0 | 1 | 0 |
| 74 | CARSPPPAYDEEEYVGFDY | 1 | 5 | 0.2 | 0 | 0 | | 67 | 0 | 0 | 1 | 51 | | 51 | 1 | 0 | 1 | 0 | 0 | 0 | 1 | 0 | 1 | 0 | 1 | 0 |
| 75 | CARSPPSDYGGPEYEGFDY | 2300 | 6961 | 0.33 | 0 | 0 | 1609 | 10280 | 0.157 | 0 | 0 | 14991 | 232 | 64.62 | 1 | 0 | 1 | 0 | 0 | 0 | 1 | 0 | 1 | 0 | 1 | 0 |
| 76 | CARSPYDFYGRQIDYLFDY | 2325 | 1 | 2325 | 1 | 0 | 228 | 152 | 1.5 | 0 | 0 | 166 | 346 | 0.48 | 0 | 0 | 1 | 0 | 1 | 0 | 0 | 0 | 1 | 0 | 1 | 0 |
| 77 | CARSRWEADRLYEYYYLFDY | | 31 | 0 | 0 | 1 | | 70 | 0 | 0 | 1 | 45 | | 45 | 1 | 0 | 1 | 0 | 0 | 0 | 1 | 0 | 1 | 0 | 1 | 0 |
| 78 | CARSRYTYPTERYGYGYFDY | 287 | 148 | 1.939 | 0 | 0 | 371 | | 371 | 1 | 0 | 402 | 25 | 16.08 | 1 | 0 | 2 | 0 | 1 | 0 | 2 | 1 | 2 | 0 | 1 | 0 |
| 79 | CARSSVIYSWYWGDGVFDY | 36 | 195 | 0.185 | 0 | 0 | 180 | | 180 | 1 | 0 | 336 | 4 | 84 | 1 | 0 | 2 | 0 | 1 | 0 | 2 | 1 | 2 | 0 | 1 | 0 |
| 80 | CARSSYDPRVFDWPDVFDY | 579 | 646 | 0.896 | 0 | 0 | 407 | 150 | 2.713 | 0 | 0 | 1538 | 46 | 33.43 | 1 | 0 | 1 | 0 | 0 | 0 | 1 | 0 | 1 | 0 | 1 | 0 |
| 81 | CARSWIDYVSQSVGPVFDY | 912 | 2 | 456 | 1 | 0 | 19 | 367 | 0.052 | 0 | 1 | 3 | 823 | 0.004 | 0 | 1 | 1 | 0 | 1 | 0 | 0 | 0 | 1 | 0 | 1 | 0 |
| 82 | CARSWQGNPTGPRYFDY | 16 | | 16 | 1 | 0 | 3 | | 3 | 0 | 0 | 6 | | 6 | 0 | 0 | 1 | 0 | 1 | 0 | 0 | 0 | 1 | 0 | 1 | 0 |

| | | | | | | | | | | | | | | | | | | | | | | | | | | |
|---|---|---|---|---|---|---|---|---|---|---|---|---|---|---|---|---|---|---|---|---|---|---|---|---|---|---|
| 83 | CARSYIWVRYGTDYDFDY | 166 | 560 | 0.296 | 0 | 0 | 730 | 44 | 16.59 | 1 | 0 | 1016 | 17 | 59.76 | 1 | 0 | 2 | 0 | 1 | 0 | 2 | 1 | 2 | 0 | 1 | 0 |
| *84* | *CARSYPHRSRRHEFAFDY* | *206* | | *206* | | | *16* | | *16* | | | *62* | *1* | *62* | | | | | | | | | *0* | *1* | | |
| 85 | CARSYVPSGSYPYGNYFFFDY | 19 | 1 | 19 | 1 | 0 | 50 | | 50 | 1 | 0 | 18 | 12 | 1.5 | 0 | 0 | 2 | 0 | 2 | 1 | 1 | 0 | 2 | 0 | 1 | 0 |
| 86 | CARSYWGLFGSWDEGFDY | 26 | 569 | 0.046 | 0 | 1 | 443 | | 443 | 1 | 0 | 682 | 4 | 170.5 | 1 | 0 | 2 | 0 | 1 | 0 | 2 | 1 | 2 | 0 | 1 | 0 |
| 87 | CARSYYGTIGSYSDAFDY | 126 | 665 | 0.189 | 0 | 0 | 623 | 7 | 89 | 1 | 0 | 1007 | 41 | 24.56 | 1 | 0 | 2 | 0 | 1 | 0 | 2 | 1 | 2 | 0 | 1 | 0 |
| 88 | CARTDYWYPERDSAWFDY | 3 | 1513 | 0.002 | 0 | 1 | | 4110 | 0 | 0 | 1 | 2816 | 37 | 76.11 | 1 | 0 | 1 | 0 | 0 | 0 | 1 | 0 | 1 | 0 | 1 | 0 |
| *89* | *CARTLRKPGDPGYPGFDY* | *902* | | *902* | | | *181* | | *181* | | | *244* | *5* | *48.8* | | | | | | | | | *0* | *1* | | |
| 90 | CARTSQYWTVDSTGFDYFDY | 2258 | 84 | 26.88 | 1 | 0 | 23 | 2124 | 0.011 | 0 | 1 | 3 | 4519 | 7E-04 | 0 | 1 | 1 | 0 | 1 | 0 | 0 | 0 | 1 | 0 | 1 | 0 |
| 91 | CARTSYVFYRSGRRGYEPFDY | 1211 | | 1211 | 1 | 0 | 236 | 1 | 236 | 1 | 0 | 91 | 318 | 0.286 | 0 | 0 | 2 | 0 | 2 | 1 | 1 | 0 | 2 | 0 | 1 | 0 |
| 92 | CARTVAWYGLWTEGFDYFDY | 47 | | 47 | 1 | 0 | 1 | 53 | 0.019 | 0 | 1 | 3 | 20 | 0.15 | 0 | 0 | 1 | 0 | 1 | 0 | 0 | 0 | 1 | 0 | 1 | 0 |
| 93 | CARTWLGLFSSDFEYFDY | | 39 | 0 | 0 | 1 | 47 | | 47 | 1 | 0 | 53 | | 53 | 1 | 0 | 2 | 0 | 1 | 0 | 2 | 1 | 2 | 0 | 1 | 0 |
| 94 | CARVFGSWDQRSGGQLYWFDY | 25 | 1 | 25 | 1 | 0 | | 45 | 0 | 0 | 1 | | 23 | 0 | 0 | 1 | 1 | 0 | 1 | 0 | 0 | 0 | 1 | 0 | 1 | 0 |
| 95 | CARVFQFYGARDYGYPYDFDY | | | | 0 | | 1 | | 1 | 0 | 0 | | 13 | 0 | 0 | 1 | 0 | 1 | 0 | 0 | 0 | 0 | 0 | 0 | 0 | 0 |
| 96 | CARVRYGDDAGRRVYTDDFDY | 20 | | 20 | 1 | 0 | 6 | | 6 | 0 | 0 | 15 | | 15 | 1 | 0 | 2 | 0 | 1 | 0 | 1 | 0 | 2 | 0 | 2 | 1 |
| 97 | CARVSGWIVVTYLYPLPQFDY | | 21 | 0 | 0 | 1 | 12 | | 12 | 1 | 0 | 35 | | 35 | 1 | 0 | 2 | 0 | 1 | 0 | 2 | 1 | 2 | 0 | 1 | 0 |
| 98 | CARVVLYWTLSSQGYDYFDY | 58 | | 58 | 1 | 0 | | 9 | 0 | 0 | | | 54 | 0 | 0 | 1 | 1 | 0 | 1 | 0 | 0 | 0 | 1 | 0 | 1 | 0 |
| 99 | CARVVRAYDAQGWWYEPFDY | 3368 | 5 | 673.6 | 1 | 0 | 476 | 466 | 1.021 | 0 | 0 | 24 | 5175 | 0.005 | 0 | 1 | 1 | 0 | 1 | 0 | 0 | 0 | 1 | 0 | 1 | 0 |
| 100 | CARVVSYYDQTGFVYWQSFDY | 1533 | | 1533 | 1 | 0 | 62 | 781 | 0.079 | 0 | 1 | 7 | 2073 | 0.003 | 0 | 1 | 1 | 0 | 1 | 0 | 0 | 0 | 1 | 0 | 1 | 0 |
| 101 | CARVVYSVSSDERTPDDYFDY | 633 | 13269 | 0.048 | 0 | 1 | 54 | 53804 | 0.001 | 0 | 1 | 84 | 69784 | 0.001 | 0 | 1 | 0 | 1 | 0 | 0 | 0 | 0 | 0 | 0 | 0 | 0 |
| 102 | CARVYPVIGRYIRDYPFDY | 269 | 236 | 1.14 | 0 | 0 | 383 | 8 | 47.88 | 1 | 0 | 537 | 16 | 33.56 | 1 | 0 | 2 | 0 | 1 | 0 | 2 | 1 | 2 | 0 | 1 | 0 |
| 103 | CARVYPVYQVRYPITWGYFDY | 89 | | 89 | 1 | 0 | 7 | 14 | 0.5 | 0 | 0 | | 31 | 0 | 0 | 1 | 1 | 0 | 1 | 0 | 0 | 0 | 1 | 0 | 1 | 0 |
| 104 | CARVYQFYGARDYGYPYDFDY | 36097 | 108 | 334.2 | 1 | 0 | 6417 | 281 | 22.84 | 1 | 0 | 54 | 51488 | 0.001 | 0 | 1 | 2 | 0 | 2 | 1 | 1 | 0 | 2 | 0 | 1 | 0 |
| 105 | CARVYVVYDRFSTYYDYFDY | 109 | | 109 | 1 | 0 | 23 | | 23 | 1 | 0 | 5 | 163 | 0.031 | 0 | 1 | 2 | 0 | 2 | 1 | 1 | 0 | 2 | 0 | 1 | 0 |
| 106 | CARWAGPFSPYDYTYPFDY | 1 | 7175 | 1E-04 | 0 | 1 | 746 | 7317 | 0.102 | 0 | 0 | 6825 | 218 | 31.31 | 1 | 0 | 1 | 0 | 0 | 0 | 1 | 0 | 1 | 0 | 1 | 0 |
| 107 | CARWGGDWDDPFDY | | 7901 | 0 | 0 | 1 | 4243 | 315 | 13.47 | 1 | 0 | 11373 | 346 | 32.87 | 1 | 0 | 2 | 0 | 1 | 0 | 2 | 1 | 2 | 0 | 1 | 0 |
| 108 | CARWPGYPVHVYTQYAVFDY | 35 | 1058 | 0.033 | 0 | 1 | | 2905 | 0 | 0 | 1 | 1533 | 21 | 73 | 1 | 0 | 1 | 0 | 0 | 0 | 1 | 0 | 1 | 0 | 1 | 0 |
| 109 | CARWYGRLDRDYREGPGYFDY | 369 | 2864 | 0.129 | 0 | 0 | 3532 | 48 | 73.58 | 1 | 0 | 6269 | 124 | 50.56 | 1 | 0 | 2 | 0 | 1 | 0 | 2 | 1 | 2 | 0 | 1 | 0 |
| 110 | CARYAGRDIGYVTRYLRFDY | 12 | 72 | 0.167 | 0 | 0 | 4 | 233 | 0.017 | 0 | 1 | 135 | 2 | 67.5 | 1 | 0 | 1 | 0 | 0 | 0 | 1 | 0 | 1 | 0 | 1 | 0 |
| 111 | CARYAGYPDGGYDPFDY | 8 | 15049 | 5E-04 | 0 | 1 | 15913 | 407 | 39.1 | 1 | 0 | 28457 | 789 | 36.07 | 1 | 0 | 2 | 0 | 1 | 0 | 2 | 1 | 2 | 0 | 1 | 0 |
| 112 | CARYAGYPSDDDDTAPFDY | | 5732 | 0 | 0 | 1 | 681 | 22839 | 0.03 | 0 | 1 | 9706 | 162 | 59.91 | 1 | 0 | 1 | 0 | 0 | 0 | 1 | 0 | 1 | 0 | 1 | 0 |
| 113 | CARYFPDYTYPDWNGYRFDY | 2984 | 7892 | 0.378 | 0 | 0 | 6 | 42391 | 1E-04 | 0 | 1 | 18272 | 400 | 45.68 | 1 | 0 | 1 | 0 | 0 | 0 | 1 | 0 | 1 | 0 | 1 | 0 |
| 114 | CARYGGPYDPTDSTYAFDY | | 1021 | 0 | 0 | 1 | 5 | 4071 | 0.001 | 0 | 1 | 1114 | 152 | 7.329 | 1 | 0 | 1 | 0 | 0 | 0 | 1 | 0 | 1 | 0 | 1 | 0 |
| 115 | CARYGYPHGWLPHGRDVYFDY | | 1807 | 0 | 0 | 1 | 22 | 3907 | 0.006 | 0 | 1 | 1855 | 40 | 46.38 | 1 | 0 | 1 | 0 | 0 | 0 | 1 | 0 | 1 | 0 | 1 | 0 |
| 116 | CARYPGVYDEDDPYYSFDY | 7 | 5582 | 0.001 | 0 | 1 | 92 | 21654 | 0.004 | 0 | 1 | 8796 | 299 | 29.42 | 1 | 0 | 1 | 0 | 0 | 0 | 1 | 0 | 1 | 0 | 1 | 0 |
| 117 | CARYQDDGPLPDVIDYFDY | 7 | 377 | 0.019 | 0 | 1 | 332 | 4 | 83 | 1 | 0 | 698 | 36 | 19.39 | 1 | 0 | 2 | 0 | 1 | 0 | 2 | 1 | 2 | 0 | 1 | 0 |
| 118 | CARYQDFRHRYYYSFDY | 176 | | 176 | 1 | 0 | 19 | 193 | 0.098 | 0 | 1 | | 431 | 0 | 0 | 1 | 1 | 0 | 1 | 0 | 0 | 0 | 1 | 0 | 1 | 0 |
| 119 | CARYRDYSHFGSSYPYAFDY | 168 | 4032 | 0.042 | 0 | 1 | 2631 | 47 | 55.98 | 1 | 0 | 5387 | 157 | 34.31 | 1 | 0 | 2 | 0 | 1 | 0 | 2 | 1 | 2 | 0 | 1 | 0 |
| 120 | CARYRPGPSYQWGRYQTFFDY | 81 | 137 | 0.591 | 0 | 0 | 160 | | 160 | 1 | 0 | 301 | 11 | 27.36 | 1 | 0 | 2 | 0 | 1 | 0 | 2 | 1 | 2 | 0 | 1 | 0 |
| 121 | CARYRVTQPAEYYSRGFDY | 787 | 838 | 0.939 | 0 | 0 | 593 | 436 | 1.36 | 0 | 0 | 1806 | 39 | 46.31 | 1 | 0 | 1 | 0 | 0 | 0 | 1 | 0 | 1 | 0 | 1 | 0 |
| 122 | CARYTYIGRQVPDARYYFDY | 687 | 1220 | 0.563 | 0 | 0 | 1445 | 19 | 76.05 | 1 | 0 | 2647 | 70 | 37.81 | 1 | 0 | 2 | 0 | 1 | 0 | 2 | 1 | 2 | 0 | 1 | 0 |
| 123 | CARYYTPPFGSPDWIVFDY | 511 | | 511 | 1 | 0 | 37 | 11 | 3.364 | 0 | 0 | 40 | 322 | 0.124 | 0 | 0 | 1 | 0 | 1 | 0 | 0 | 0 | 1 | 0 | 1 | 0 |

**Table S3.** $IC_{50}$ values and potency for neutralization of Wuhan-1 SARS-CoV-2 PSV by $bsNb_4$-Igs (Class 2 nanobody as HC/LM18 as LC, top and Class 4 nanobody as HC/LM18 as LC, bottom). Assays were run in duplicate to select for the best constructs to be tested with other PSVs.

| **Nb HC/Nb LC** | $IC_{50}$ (μg/mL) | $IC_{50}$ (nM) | % Neut | $R^2$ |
|---|---|---|---|---|
| 109/LM18 | 0.274 | 1.825 | 99 | 0.97 |
| 112/LM18 | 0.588 | 3.918 | 88 | 0.97 |
| 115/LM18 | 0.194 | 1.291 | 101 | 0.96 |
| 118/LM18 | 0.230 | 1.532 | 99 | 0.98 |
| 121/LM18 | 0.056 | 0.374 | 96 | 1.00 |
| 124/LM18 | 0.285 | 1.903 | 101 | 0.99 |
| 127/LM18 | 1.709 | 11.393 | 87 | 0.97 |
| 130/LM18 | 0.138 | 0.922 | 99 | 0.99 |
| 133/LM18 | 0.306 | 2.041 | 89 | 0.96 |
| 136/LM18 | 0.126 | 0.837 | 99 | 0.97 |
| 139/LM18 | 0.234 | 1.561 | 98 | 0.99 |
| 142/LM18 | 0.112 | 0.744 | 96 | 0.98 |
| 145/LM18 | 0.023 | 0.154 | 95 | 0.98 |
| 148/LM18 | 0.170 | 1.135 | 97 | 0.98 |
| 154/LM18 | 1.914 | 12.760 | 91 | 0.98 |
| 157/LM18 | 0.200 | 1.334 | 92 | 0.98 |
| 160/LM18 | 3.250 | 21.667 | 92 | 0.98 |
| 163/LM18 | 0.044 | 0.295 | 94 | 0.99 |
| 166/LM18 | 0.165 | 1.099 | 99 | 0.99 |
| 169/LM18 | 1.703 | 11.353 | 88 | 0.96 |

| **Nb HC/Nb LC** | $IC_{50}$ (μg/mL) | $IC_{50}$ (nM) | % Neut | $R^2$ |
|---|---|---|---|---|
| 186/LM18 | 0.189 | 1.260 | 97 | 0.98 |
| 189/LM18 | 0.106 | 0.707 | 97 | 0.99 |
| 192/LM18 | 1.084 | 7.227 | 65 | 0.89 |
| 195/LM18 | 0.273 | 1.820 | 99 | 0.97 |
| 198/LM18 | 0.029 | 0.193 | 99 | 1.00 |
| 201/LM18 | 4.373 | 29.153 | 45 | 0.90 |
| 204/LM18 | 1.506 | 10.040 | 92 | 0.98 |
| 207/LM18 | 4.122 | 27.480 | 61 | 0.87 |
| 210/LM18 | 0.157 | 1.047 | 99 | 0.98 |
| 213/LM18 | 0.240 | 1.600 | 96 | 0.98 |
| 216/LM18 | 1.932 | 12.880 | 66 | 0.91 |
| 219/LM18 | 0.130 | 0.867 | 97 | 0.99 |
| 222/LM18 | 4.280 | 28.533 | 55 | 0.93 |
| 225/LM18 | 0.070 | 0.467 | 97 | 0.97 |
| 228/LM18 | 7.972 | 53.147 | 77 | 0.91 |
| 231/LM18 | 3.377 | 22.513 | 83 | 0.94 |
| 234/LM18 | 0.427 | 2.847 | 101 | 0.97 |
| 237/LM18 | 0.021 | 0.140 | 97 | 0.89 |
| 240/LM18 | 0.194 | 1.293 | 95 | 0.93 |
| 243/LM18 | 0.466 | 3.107 | 98 | 0.97 |
| 246/LM18 | 3.874 | 25.827 | 79 | 0.88 |
| 249/LM18 | 38.720 | 258.133 | 88 | 0.81 |
| 252/LM18 | 1.096 | 7.307 | 64 | 0.74 |
| 255/LM18 | 0.037 | 0.247 | 97 | 0.96 |

**Table S4.** $IC_{50}$ values and potency for neutralization of SARS-CoV-2 PSVs by a selection of Nb-C2/LM18 and Nb-C4/LM18 $bsNb_4$-Igs . No notable difference for neutralization between $bsNb_4$-Igs with LM18 either as a HC or LC could be observed. Assays were run in duplicate. wt =Wuhan-1, NN = non-neutralizing, nd = not determined, n.a = not applicable.

| **Nb121/LM18** | $IC_{50}$ (µg/mL) | $IC_{50}$ (nM) | % Neut | $R^2$ |
|---|---|---|---|---|
| wt | 0.082 | 0.545 | 95 | 0.95 |
| *beta* | 2.102 | 14.013 | 102 | 0.97 |
| *gamma* | 0.779 | 5.192 | 104 | 0.93 |
| *kappa* | 1.114 | 7.427 | 45 | 0.66 |
| *delta* | 3.556 | 23.707 | 99 | 0.93 |
| L452R | 2.777 | 18.513 | 87 | 0.90 |
| E484Q | 0.791 | 5.273 | 96 | 0.92 |

| **LM18/Nb121** | $IC_{50}$ (µg/mL) | $IC_{50}$ (nM) | % Neut | $R^2$ |
|---|---|---|---|---|
| wt | 0.043 | 0.286 | 95 | 0.97 |
| *beta* | 1.280 | 8.533 | 104 | 0.95 |
| *gamma* | 0.396 | 2.639 | 102 | 0.92 |
| *kappa* | 0.560 | 3.735 | 70 | 0.83 |
| *delta* | nd | nd | nd | nd |
| L452R | 1.822 | 12.147 | 90 | 0.95 |
| E484Q | 0.417 | 2.777 | 97 | 0.95 |

| **Nb136/LM18** | $IC_{50}$ (µg/mL) | $IC_{50}$ (nM) | % Neut | $R^2$ |
|---|---|---|---|---|
| wt | 0.177 | 1.181 | 100 | 0.98 |
| *beta* | 0.162 | 1.079 | 96 | 0.93 |
| *gamma* | 0.120 | 0.800 | 99 | 0.94 |
| *kappa* | 0.099 | 0.658 | 93 | 0.92 |
| *delta* | 0.074 | 0.493 | 100 | 0.98 |
| L452R | 0.093 | 0.622 | 101 | 0.97 |
| E484Q | 0.101 | 0.673 | 100 | 0.92 |

| **LM18/Nb136** | $IC_{50}$ (µg/mL) | $IC_{50}$ (nM) | % Neut | $R^2$ |
|---|---|---|---|---|
| wt | 0.167 | 1.112 | 100 | 0.96 |
| *beta* | 0.087 | 0.577 | 96 | 0.93 |
| *gamma* | 0.051 | 0.340 | 97 | 0.89 |
| *kappa* | 0.116 | 0.776 | 96 | 0.97 |
| *delta* | nd | nd | nd | nd |
| L452R | 0.153 | 1.017 | 102 | 0.90 |
| E484Q | 0.455 | 3.030 | 96 | 0.86 |

| **Nb139/LM18** | $IC_{50}$ (µg/mL) | $IC_{50}$ (nM) | % Neut | $R^2$ |
|---|---|---|---|---|
| wt | 0.150 | 0.998 | 102 | 0.94 |
| *alpha* | 0.150 | 1.001 | 100 | 0.94 |
| *beta* | 0.193 | 1.284 | 100 | 0.93 |
| *gamma* | 0.206 | 1.373 | 104 | 0.89 |
| L452R | NN | NN | n.a | n.a |

| **LM18/Nb139** | $IC_{50}$ (µg/mL) | $IC_{50}$ (nM) | % Neut | $R^2$ |
|---|---|---|---|---|
| wt | 0.013 | 0.085 | 101 | 0.92 |
| *alpha* | 0.021 | 0.142 | 104 | 0.95 |
| *beta* | 0.106 | 0.706 | 98 | 0.94 |
| *gamma* | 0.173 | 1.154 | 106 | 0.92 |
| L452R | NN | NN | n.a | n.a |

| **Nb148/LM18** | $IC_{50}$ (µg/mL) | $IC_{50}$ (nM) | % Neut | $R^2$ |
|---|---|---|---|---|
| wt | 0.209 | 1.395 | 101 | 0.96 |
| *alpha* | 0.188 | 1.250 | 97 | 0.94 |
| *beta* | 0.484 | 3.223 | 94 | 0.95 |
| *gamma* | 0.963 | 6.421 | 108 | 0.91 |
| L452R | NN | NN | n.a | n.a |

| **LM18/Nb148** | $IC_{50}$ (µg/mL) | $IC_{50}$ (nM) | % Neut | $R^2$ |
|---|---|---|---|---|
| wt | 0.057 | 0.378 | 105 | 0.97 |
| *alpha* | 0.056 | 0.374 | 101 | 0.98 |
| *beta* | 0.272 | 1.813 | 98 | 0.98 |
| *gamma* | 0.682 | 4.545 | 111 | 0.93 |
| L452R | NN | NN | n.a | n.a |

| **Nb198/LM18** | $IC_{50}$ (µg/mL) | $IC_{50}$ (nM) | % Neut | $R^2$ |
|---|---|---|---|---|
| wt | 0.035 | 0.231 | 101 | 1.00 |
| *beta* | 0.008 | 0.053 | 98 | 0.94 |
| *gamma* | 0.012 | 0.082 | 97 | 0.98 |
| *kappa* | 0.030 | 0.200 | 101 | 0.99 |
| *delta* | 0.018 | 0.12 | 100 | 0.98 |
| L452R | 0.048 | 0.320 | 102 | 0.98 |
| E484Q | 0.044 | 0.290 | 101 | 0.98 |

| **LM18/Nb198** | $IC_{50}$ (µg/mL) | $IC_{50}$ (nM) | % Neut | $R^2$ |
|---|---|---|---|---|
| wt | 0.046 | 0.309 | 100 | 0.99 |
| *beta* | 0.026 | 0.171 | 101 | 0.98 |
| *gamma* | 0.017 | 0.110 | 98 | 0.94 |
| *kappa* | 0.032 | 0.213 | 100 | 0.99 |
| *delta* | nd | nd | nd | nd |
| L452R | 0.082 | 0.545 | 104 | 0.95 |
| E484Q | 0.081 | 0.539 | 102 | 0.99 |

| **Nb225/LM18** | $IC_{50}$ (µg/mL) | $IC_{50}$ (nM) | % Neut | $R^2$ |
|---|---|---|---|---|
| wt | 0.043 | 0.284 | 101 | 0.94 |
| *beta* | 0.007 | 0.046 | 99 | 0.98 |
| *gamma* | 0.010 | 0.066 | 98 | 0.98 |
| *kappa* | 0.011 | 0.071 | 100 | 0.97 |
| *delta* | 0.013 | 0.087 | 101 | 0.98 |
| L452R | 0.061 | 0.407 | 103 | 0.97 |
| E484Q | 0.027 | 0.179 | 99 | 0.98 |

| **LM18/Nb225** | $IC_{50}$ (µg/mL) | $IC_{50}$ (nM) | % Neut | $R^2$ |
|---|---|---|---|---|
| wt | 0.042 | 0.281 | 98 | 0.93 |
| *beta* | 0.005 | 0.033 | 97 | 0.89 |
| *gamma* | 0.010 | 0.065 | 97 | 0.97 |
| *kappa* | 0.019 | 0.124 | 100 | 0.95 |
| *delta* | nd | nd | nd | nd |
| L452R | 0.117 | 0.781 | 101 | 0.87 |
| E484Q | 0.050 | 0.332 | 99 | 0.96 |

| **Nb237/LM18** | $IC_{50}$ (µg/mL) | $IC_{50}$ (nM) | % Neut | $R^2$ |
|---|---|---|---|---|
| wt | 0.011 | 0.074 | 100 | 0.97 |
| *beta* | 0.008 | 0.053 | 101 | 0.99 |
| *gamma* | 0.006 | 0.038 | 97 | 0.97 |
| *kappa* | 0.006 | 0.042 | 100 | 0.98 |
| *delta* | 0.003 | 0.020 | 100 | 1.00 |
| L452R | 0.018 | 0.117 | 100 | 1.00 |
| E484Q | 0.018 | 0.121 | 102 | 0.99 |

| **LM18/Nb237** | $IC_{50}$ (µg/mL) | $IC_{50}$ (nM) | % Neut | $R^2$ |
|---|---|---|---|---|
| wt | 0.021 | 0.140 | 101 | 0.89 |
| *beta* | 0.018 | 0.120 | 100 | 0.88 |
| *gamma* | 0.013 | 0.087 | 96 | 0.79 |
| *kappa* | 0.014 | 0.093 | 100 | 0.90 |
| *delta* | nd | nd | nd | nd |
| L452R | 0.029 | 0.193 | 99 | 0.92 |
| E484Q | 0.027 | 0.180 | 99 | 0.87 |

| **Nb240/LM18** | $IC_{50}$ (µg/mL) | $IC_{50}$ (nM) | % Neut | $R^2$ |
|---|---|---|---|---|
| wt | 0.367 | 2.447 | 104 | 0.97 |
| *beta* | 0.046 | 0.307 | 99 | 0.93 |
| *gamma* | 0.024 | 0.160 | 98 | 0.97 |
| *kappa* | 0.074 | 0.493 | 102 | 0.98 |
| *delta* | 0.027 | 0.180 | 100 | 0.99 |
| L452R | 0.213 | 1.420 | 98 | 0.97 |
| E484Q | 0.406 | 2.707 | 100 | 0.91 |

| **LM18/Nb240** | $IC_{50}$ (µg/mL) | $IC_{50}$ (nM) | % Neut | $R^2$ |
|---|---|---|---|---|
| wt | 0.121 | 0.803 | 102 | 0.99 |
| *beta* | 0.063 | 0.418 | 102 | 0.95 |
| *gamma* | 0.028 | 0.186 | 98 | 0.98 |
| *kappa* | 0.056 | 0.372 | 101 | 0.97 |
| *delta* | nd | nd | nd | nd |
| L452R | 0.282 | 1.882 | 101 | 0.98 |
| E484Q | 0.313 | 2.084 | 101 | 0.96 |

| **Nb255/LM18** | $IC_{50}$ (µg/mL) | $IC_{50}$ (nM) | % Neut | $R^2$ |
|---|---|---|---|---|
| wt | 0.028 | 0.187 | 101 | 0.99 |
| *beta* | 0.006 | 0.040 | 99 | 0.98 |
| *gamma* | 0.004 | 0.027 | 97 | 0.97 |
| *kappa* | 0.006 | 0.040 | 99 | 0.99 |
| *delta* | 0.013 | 0.087 | 100 | 0.97 |
| L452R | 0.024 | 0.160 | 99 | 0.99 |
| E484Q | 0.012 | 0.080 | 99 | 0.95 |

| **LM18/Nb255** | $IC_{50}$ (µg/mL) | $IC_{50}$ (nM) | % Neut | $R^2$ |
|---|---|---|---|---|
| wt | 0.024 | 0.158 | 100 | 0.96 |
| *beta* | 0.003 | 0.021 | 98 | 0.96 |
| *gamma* | 0.003 | 0.023 | 96 | 0.96 |
| *kappa* | 0.005 | 0.036 | 99 | 0.97 |
| *delta* | nd | nd | nd | nd |
| L452R | 0.024 | 0.157 | 98 | 0.97 |
| E484Q | 0.014 | 0.095 | 98 | 0.96 |

**Table S5.** $IC_{50}$ values and potency for neutralization of SARS-CoV-2 PSVs by class 2 and 4 nanobody-based bsNb$_4$-Igs. Assays were run in duplicate. wt = Wuhan-1, NN = non neutralizing, n.a = not applicable

| **121/255** | $IC_{50}$ (μg/mL) | $IC_{50}$ (nM) | % Neut | $R^2$ |
|---|---|---|---|---|
| wt | 0.353 | 2.353 | 103 | 0.91 |
| *beta* | NN | NN | n.a | n.a |
| *gamma* | NN | NN | n.a | n.a |
| *kappa* | NN | NN | n.a | n.a |
| L452R | NN | NN | n.a | n.a |
| E484Q | 3.753 | 25.020 | 63 | 0.86 |

| **198/121** | $IC_{50}$ (μg/mL) | $IC_{50}$ (nM) | % Neut | $R^2$ |
|---|---|---|---|---|
| wt | 0.326 | 2.173 | 103 | 0.98 |
| *beta* | NN | NN | n.a | n.a |
| *gamma* | NN | NN | n.a | n.a |
| *kappa* | NN | NN | n.a | n.a |
| L452R | NN | NN | n.a | n.a |
| E484Q | NN | NN | n.a | n.a |

| **136/198** | $IC_{50}$ (μg/mL) | $IC_{50}$ (nM) | % Neut | $R^2$ |
|---|---|---|---|---|
| wt | 1.035 | 6.900 | 104 | 0.99 |
| *beta* | 8.134 | 54.227 | 87 | 0.93 |
| *gamma* | 3.852 | 25.680 | 96 | 0.95 |
| *kappa* | 3.553 | 23.687 | 88 | 0.97 |
| *delta* | 5.129 | 34.193 | 101 | 0.93 |

| **198/136** | $IC_{50}$ (μg/mL) | $IC_{50}$ (nM) | % Neut | $R^2$ |
|---|---|---|---|---|
| wt | 0.878 | 5.856 | 105 | 0.98 |
| *beta* | 2.579 | 17.193 | 79 | 0.95 |
| *gamma* | 1.302 | 8.680 | 93 | 0.96 |
| *kappa* | 2.840 | 18.933 | 90 | 0.96 |
| *delta* | 5.194 | 34.627 | 108 | 0.95 |

| **136/225** | $IC_{50}$ (μg/mL) | $IC_{50}$ (nM) | % Neut | $R^2$ |
|---|---|---|---|---|
| wt | 0.083 | 0.556 | 101 | 0.99 |
| *beta* | 0.251 | 1.673 | 89 | 0.98 |
| *gamma* | 0.055 | 0.365 | 93 | 0.99 |
| *kappa* | 0.174 | 1.159 | 91 | 0.99 |
| *delta* | 0.161 | 1.074 | 103 | 0.96 |

| **225/136** | $IC_{50}$ (μg/mL) | $IC_{50}$ (nM) | % Neut | $R^2$ |
|---|---|---|---|---|
| wt | 0.170 | 1.135 | 102 | 0.99 |
| *beta* | 2.281 | 15.207 | 84 | 0.98 |
| *gamma* | 0.385 | 2.564 | 92 | 0.99 |
| *kappa* | 0.371 | 2.471 | 86 | 0.99 |
| *delta* | 0.724 | 4.827 | 98 | 0.96 |

| **136/237** | $IC_{50}$ (μg/mL) | $IC_{50}$ (nM) | % Neut | $R^2$ |
|---|---|---|---|---|
| wt | 0.684 | 4.560 | 103 | 0.98 |
| *beta* | 0.443 | 2.956 | 89 | 0.97 |
| *gamma* | 0.341 | 2.273 | 97 | 0.99 |
| *kappa* | 0.691 | 4.606 | 94 | 0.99 |
| *delta* | 0.712 | 4.745 | 103 | 0.98 |

| **237/136** | $IC_{50}$ (μg/mL) | $IC_{50}$ (nM) | % Neut | $R^2$ |
|---|---|---|---|---|
| wt | 0.398 | 2.653 | 102 | 0.99 |
| *beta* | 0.587 | 3.911 | 89 | 0.98 |
| *gamma* | 0.227 | 1.512 | 95 | 0.98 |
| *kappa* | 0.409 | 2.723 | 90 | 0.99 |
| *delta* | 0.811 | 5.403 | 104 | 0.96 |

| **136/240** | $IC_{50}$ (μg/mL) | $IC_{50}$ (nM) | % Neut | $R^2$ |
|---|---|---|---|---|
| wt | 0.413 | 2.752 | 101 | 0.99 |
| *beta* | 0.808 | 5.385 | 86 | 0.99 |
| *gamma* | 0.228 | 1.517 | 92 | 0.98 |
| *kappa* | 1.116 | 7.440 | 92 | 0.99 |
| *delta* | 1.301 | 8.673 | 103 | 0.96 |

| **240/136** | $IC_{50}$ (μg/mL) | $IC_{50}$ (nM) | % Neut | $R^2$ |
|---|---|---|---|---|
| wt | 0.283 | 1.887 | 101 | 0.99 |
| *beta* | 1.578 | 10.520 | 84 | 0.95 |
| *gamma* | 0.379 | 2.529 | 94 | 0.99 |
| *kappa* | 0.907 | 6.045 | 90 | 0.91 |
| *delta* | 1.606 | 10.707 | 102 | 0.99 |

| **136/255** | $IC_{50}$ (μg/mL) | $IC_{50}$ (nM) | % Neut | $R^2$ |
|---|---|---|---|---|
| wt | 0.122 | 0.813 | 99 | 0.98 |
| *beta* | 0.660 | 4.402 | 89 | 0.98 |
| *gamma* | 0.151 | 1.009 | 92 | 0.98 |
| *kappa* | 0.523 | 3.489 | 91 | 0.99 |
| *delta* | 0.669 | 4.461 | 103 | 0.96 |

| **255/136** | $IC_{50}$ (μg/mL) | $IC_{50}$ (nM) | % Neut | $R^2$ |
|---|---|---|---|---|
| wt | 0.234 | 1.558 | 103 | 0.98 |
| *beta* | 0.961 | 6.409 | 88 | 0.99 |
| *gamma* | 0.246 | 1.637 | 95 | 0.99 |
| *kappa* | 0.404 | 2.695 | 89 | 0.97 |
| *delta* | 0.692 | 4.615 | 102 | 0.98 |

**Table S6.** $IC_{50}$ values and potency for neutralization of SARS-CoV-2 *omicron* PSV by LM18- and Nb-C2-136-based $bsNb_4$-Igs. Monoclonal antibodies CC12.1 and CC6.30, as well as the clinical antibody candidates LY-CoV555, REGN-10933 and REGN-10987 were tested for reference. Assays were run in duplicate. NN: not neutralizing, n.a: not applicable

| **Construct** | | $IC_{50}$ ($\mu$g/mL) | $IC_{50}$ (nM) | % Neut | $R^2$ |
|---|---|---|---|---|---|
| **HC** | **LC** | | | | |
| Nb-C4-198 | LM18 | NN | NN | n.a | n.a |
| Nb-C4-225 | | NN | NN | n.a | n.a |
| Nb-C4-237 | | NN | NN | n.a | n.a |
| Nb-C4-240 | | NN | NN | n.a | n.a |
| Nb-C4-255 | | NN | NN | n.a | n.a |
| Nb-C4-198 | Nb-C2-136 | 0.713 | 4.75 | 104 | 0.94 |
| Nb-C4-225 | | 0.418 | 2.79 | 103 | 0.97 |
| Nb-C4-237 | | 0.343 | 2.29 | 102 | 0.96 |
| Nb-C4-240 | | 0.670 | 4.47 | 103 | 0.96 |
| Nb-C4-255 | | 0.379 | 2.53 | 102 | 0.96 |
| CC12.1 | | NN | NN | n.a | n.a |
| CC6.30 | | NN | NN | n.a | n.a |
| LY-CoV555 | | NN | NN | n.a | n.a |
| REGN10933 | | NN | NN | n.a | n.a |
| REGN10987 | | NN | NN | n.a | n.a |

**Table S7.** $IC_{50}$ values and potency for neutralization of SARS-CoV-2 PSVs by $Nb_4$-Igs (tetravalent monospecific constructs). Assays were run in duplicate. NN: not neutralizing, n.a: not applicable, nt: not tested. wt = Wuhan-1

| **tetraLM18** | $IC_{50}$ (μg/mL) | $IC_{50}$ (nM) | % Neut | $R^2$ |
|---|---|---|---|---|
| wt | 0.029 | 0.193 | 96 | 0.99 |
| *beta* | 0.025 | 0.167 | 99 | 0.97 |
| *gamma* | 0.014 | 0.093 | 89 | 0.98 |
| *kappa* | 0.071 | 0.473 | 87 | 0.96 |
| *delta* | 0.050 | 0.333 | 100 | 0.99 |

| **tetra121** | $IC_{50}$ (μg/mL) | $IC_{50}$ (nM) | % Neut | $R^2$ |
|---|---|---|---|---|
| wt | NN | NN | n.a | n.a |
| *beta* | NN | NN | n.a | n.a |
| *gamma* | NN | NN | n.a | n.a |
| *kappa* | NN | NN | n.a | n.a |
| *delta* | NN | NN | n.a | n.a |

| **tetra136** | $IC_{50}$ (μg/mL) | $IC_{50}$ (nM) | % Neut | $R^2$ |
|---|---|---|---|---|
| wt | 0.362 | 2.413 | 98 | 0.96 |
| *beta* | NN | NN | n.a | n.a |
| *gamma* | NN | NN | n.a | n.a |
| *kappa* | NN | NN | n.a | n.a |
| *delta* | NN | NN | n.a | n.a |

| **tetra240** | $IC_{50}$ (μg/mL) | $IC_{50}$ (nM) | % Neut | $R^2$ |
|---|---|---|---|---|
| wt | NN | NN | n.a | n.a |
| *beta* | NN | NN | n.a | n.a |
| *gamma* | NN | NN | n.a | n.a |
| *kappa* | NN | NN | n.a | n.a |
| *delta* | NN | NN | n.a | n.a |

| **tetra225** | $IC_{50}$ (μg/mL) | $IC_{50}$ (nM) | % Neut | $R^2$ |
|---|---|---|---|---|
| wt | NN | NN | n.a | n.a |
| *beta* | nt | nt | nt | nt |
| *gamma* | nt | nt | nt | nt |
| *kappa* | nt | nt | nt | nt |
| *delta* | NN | NN | n.a | n.a |

| **tetra246** | $IC_{50}$ (μg/mL) | $IC_{50}$ (nM) | % Neut | $R^2$ |
|---|---|---|---|---|
| wt | NN | NN | n.a | n.a |
| *beta* | NN | NN | n.a | n.a |
| *gamma* | NN | NN | n.a | n.a |
| *kappa* | NN | NN | n.a | n.a |
| *delta* | NN | NN | n.a | n.a |

**Table S8.** Summarized results of Wuhan-1 SARS-CoV-2-RBD binding to nanobodies, Fabs and antibodies. Association and dissociation rate constants calculated through a 1:1 Langmuir binding model when possible or heterologous ligand binding model using the BIAevaluation software.

| Constructs | HC/LC | Type | Method | Fit Model | $k_a1$ | $k_d1$ | $K_D1$ | $k_a2$ | $k_d2$ | $K_D2$ |
|---|---|---|---|---|---|---|---|---|---|---|
| **LM18** | - | Nb | Direct, RBD conj. | 1:1 Binding | 2.41E+05 | 9.44E-02 | 3.91E-07 | - | - | - |
| **LM18.1.17** | - | Nb | Direct, RBD conj. | 1:1 Binding | 5.51E+05 | 5.19E-02 | 9.42E-08 | - | - | - |
| **Nb121** | - | Nb | Direct, RBD conj. | 1:1 Binding | 2.07E+05 | 2.09E-01 | 1.01E-06 | - | - | - |
| **Nb136** | - | Nb | Direct, RBD conj. | 1:1 Binding | 8.37E+04 | 3.20E-02 | 3.82E-07 | - | - | - |
| **Nb198** | - | Nb | Direct, RBD conj. | 1:1 Binding | 6.93E+04 | 1.87E-01 | 2.96E-06 | - | - | - |
| **Nb225** | - | Nb | Direct, RBD conj. | 1:1 Binding | 3.99E+04 | 5.70E-03 | 1.43E-07 | - | - | - |
| **Nb237** | - | Nb | Direct, RBD conj. | 1:1 Binding | 1.09E+05 | 5.69E-02 | 5.21E-07 | - | - | - |
| **Nb240** | - | Nb | Direct, RBD conj. | 1:1 Binding | 9.11E+05 | 9.69E-01 | 1.06E-06 | - | - | - |
| **Nb255** | - | Nb | Direct, RBD conj. | 1:1 Binding | 9.99E+04 | 2.95E-01 | 2.95E-06 | - | - | - |
| **Ab122** | 121/LM18 | Ab | Fc-cap, multi-cycle | 1:1 Binding | 1.57E+06 | 4.13E-03 | 2.63E-09 | - | - | - |
| **Ab122.1** | 121/LM18.1.17 | Ab | Fc-cap, multi-cycle | 1:1 Binding | 1.82E+06 | 1.60E-03 | 8.76E-10 | - | - | - |
| **Ab123** | LM18/121 | Ab | Fc-cap, multi-cycle | 1:1 Binding | 1.79E+06 | 5.24E-03 | 2.94E-09 | - | - | - |
| **Ab137** | 136/LM18 | Ab | Fc-cap, multi-cycle | 1:1 Binding | 7.45E+05 | 4.25E-04 | 5.70E-10 | - | - | - |
| **Ab137.1** | 136/LM18.1.17 | Ab | Fc-cap, multi-cycle | 1:1 Binding | 1.11E+06 | 3.29E-04 | 2.96E-10 | - | - | - |
| **Ab138** | LM18/136 | Ab | Fc-cap, multi-cycle | 1:1 Binding | 7.08E+05 | 3.54E-04 | 4.99E-10 | - | - | - |
| **Ab199** | 198/LM18 | Ab | Fc-cap, multi-cycle | 1:1 Binding | 6.37E+05 | 1.76E-02 | 2.76E-08 | - | - | - |
| **Ab199.1** | 198/LM18.1.17 | Ab | Fc-cap, multi-cycle | 1:1 Binding | 3.15E+06 | 3.34E-02 | 1.06E-08 | - | - | - |
| **Ab200** | LM18/198 | Ab | Fc-cap, multi-cycle | 1:1 Binding | 6.12E+05 | 1.59E-02 | 2.60E-08 | - | - | - |
| **Ab226** | 225/LM18 | Ab | Fc-cap, multi-cycle | *Het. Ligand* | *1.22E+06* | *2.40E-01* | *1.97E-07* | *7.67E+04* | *9.48E-04* | *1.24E-08* |
| **Ab226.1** | 225/LM18.1.17 | Ab | Fc-cap, multi-cycle | *Het. Ligand* | *3.03E+06* | *1.20E-01* | *3.96E-08* | *8.31E+04* | *2.71E-04* | *3.26E-09* |
| **Ab227** | LM18/225 | Ab | Fc-cap, multi-cycle | *Het. Ligand* | *2.90E+06* | *2.15E-01* | *7.42E-08* | *6.91E+04* | *1.32E-03* | *1.91E-08* |
| **Ab238** | 237/LM18 | Ab | Fc-cap, multi-cycle | *Het. Ligand* | *3.77E+05* | *1.04E-01* | *2.75E-07* | *5.61E+04* | *1.74E-04* | *3.10E-09* |
| **Ab238.1** | 237/LM18.1.17 | Ab | Fc-cap, multi-cycle | 1:1 Binding | 4.85E+05 | 3.54E-02 | 7.31E-08 | - | - | - |
| **Ab239** | LM18/237 | Ab | Fc-cap, multi-cycle | *Het. Ligand* | *4.12E+05* | *1.05E-01* | *2.56E-07* | *7.22E+04* | *1.19E-04* | *1.64E-09* |
| **Ab241** | 240/LM18 | Ab | Fc-cap, multi-cycle | *Het. Ligand* | *9.32E+05* | *1.35E-01* | *1.45E-07* | *8.54E+04* | *4.41E-04* | *5.17E-09* |
| **Ab241.1** | 240/LM18.1.17 | Ab | Fc-cap, multi-cycle | *Het. Ligand* | *1.47E+06* | *7.89E-02* | *5.36E-08* | *1.17E+05* | *9.00E-05* | *7.66E-10* |
| **Ab242** | LM18/240 | Ab | Fc-cap, multi-cycle | *Het. Ligand* | *1.24E+06* | *1.95E-01* | *1.58E-07* | *5.99E+04* | *4.97E-04* | *8.29E-09* |
| **Ab256** | 255/LM18 | Ab | Fc-cap, multi-cycle | *Het. Ligand* | *1.09E+06* | *2.06E-01* | *1.89E-07* | *5.82E+04* | *3.95E-04* | *6.80E-09* |
| **Ab256.1** | 255/LM18.1.17 | Ab | Fc-cap, multi-cycle | *Het. Ligand* | *2.17E+06* | *1.36E-01* | *6.27E-08* | *9.94E+04* | *3.53E-05* | *3.55E-10* |
| **Ab257** | LM18/255 | Ab | Fc-cap, multi-cycle | *Het. Ligand* | *1.51E+06* | *2.32E-01* | *1.54E-07* | *5.41E+04* | *7.38E-04* | *1.36E-08* |
| **TetraLM18** | LM18/LM18 | Ab | Fc-cap, multi-cycle | *Het. Ligand* | *5.59E+05* | *1.38E-01* | *2.47E-07* | *6.33E+04* | *2.99E-04* | *4.72E-09* |
| **Fab123** | LM18/121 | Fab | RBD-biotin-CAP, single-cycle | 1:1 Binding | 1.14E+06 | 2.11E-02 | 1.85E-08 | - | - | - |
| **Fab138** | LM18/136 | Fab | RBD-biotin-CAP, single-cycle | 1:1 Binding | 4.17E+05 | 6.78E-05 | 1.63E-10 | - | - | - |
| **Fab200** | LM18/198 | Fab | RBD-biotin-CAP, single-cycle | 1:1 Binding | 2.10E+06 | 5.39E-02 | 2.57E-08 | - | - | - |
| **Fab227** | LM18/225 | Fab | RBD-biotin-CAP, single-cycle | 1:1 Binding | 6.05E+06 | 5.36E-02 | 8.69E-09 | - | - | - |
| **Fab239** | LM18/237 | Fab | RBD-biotin-CAP, single-cycle | *Het. Ligand* | *6.11E+06* | *4.12E-02* | *6.74E-09* | *3.33E+05* | *1.93E-03* | *5.80E-09* |
| **Fab242** | LM18/240 | Fab | RBD-biotin-CAP, single-cycle | 1:1 Binding | 2.49E+06 | 7.01E-02 | 2.81E-08 | - | - | - |
| **Fab257** | LM18/255 | Fab | RBD-biotin-CAP, single-cycle | *Het. Ligand* | *6.43E+06* | *5.01E-02* | *7.80E-09* | *2.26E+05* | *2.88E-03* | *1.27E-08* |

**Table S9.** Summarized results of Wuhan-1 SARS-CoV-2-Spike binding to Fabs or antibodies. Association and dissociation rate constants calculated through a 1:1 Langmuir binding model using the BIAevaluation software.

| Constructs | HC/LC | Type | Method | Fit Model | $k_a$1 | $k_d$1 | $K_D$1 |
|---|---|---|---|---|---|---|---|
| **Ab123** | LM18/121 | Ab | Fc-cap, multi-cycle | 1:1 Binding | 5.83E+05 | 6.29E-04 | 1.08E-09 |
| **Ab138** | LM18/136 | Ab | Fc-cap, multi-cycle | 1:1 Binding | 5.09E+05 | 3.23E-04 | 6.35E-10 |
| **Ab200** | LM18/198 | Ab | Fc-cap, multi-cycle | 1:1 Binding | 5.25E+05 | 6.77E-04 | 1.29E-09 |
| **Ab227** | LM18/225 | Ab | Fc-cap, multi-cycle | 1:1 Binding | 1.50E+06 | 1.74E-03 | 1.16E-09 |
| **Ab239** | LM18/237 | Ab | Fc-cap, multi-cycle | 1:1 Binding | 3.45E+05 | 6.45E-04 | 1.87E-09 |
| **Ab242** | LM18/240 | Ab | Fc-cap, multi-cycle | 1:1 Binding | 4.85E+05 | 1.77E-03 | 3.65E-09 |
| **Ab257** | LM18/255 | Ab | Fc-cap, multi-cycle | 1:1 Binding | 1.00E+06 | 9.80E-04 | 9.77E-10 |
| **TetraLM18** | LM18/LM18 | Ab | Fc-cap, multi-cycle | 1: 1 Binding | 5.77E+05 | 1.09E-03 | 1.88E-09 |
| **Fab123** | LM18/121 | Fab | S-Biotin-CAP, single-cycle | 1:1 Binding | 1.18E+06 | 1.44E-02 | 1.22E-08 |
| **Fab138** | LM18/136 | Fab | S-Biotin-CAP, single-cycle | 1:1 Binding | 7.28E+05 | 3.06E-05 | 4.20E-11 |
| **Fab200** | LM18/198 | Fab | S-Biotin-CAP, single-cycle | 1:1 Binding | 3.09E+06 | 2.09E-02 | 6.75E-09 |
| **Fab227** | LM18/225 | Fab | S-Biotin-CAP, single-cycle | 1:1 Binding | 4.58E+06 | 2.96E-02 | 6.47E-09 |
| **Fab239** | LM18/237 | Fab | S-Biotin-CAP, single-cycle | 1:1 Binding | 2.63E+06 | 1.63E-03 | 6.19E-10 |
| **Fab242** | LM18/240 | Fab | S-Biotin-CAP, single-cycle | 1:1 Binding | 1.18E+06 | 2.13E-02 | 1.80E-08 |
| **Fab257** | LM18/255 | Fab | S-Biotin-CAP, single-cycle | 1:1 Binding | 3.56E+06 | 9.60E-03 | 2.69E-09 |

**Table S10.** Neutralization of SARS-CoV-2 PSVs by matured LM18-based bsNb$_4$-Igs. Assays were run in duplicate. wt = Wuhan-1

| Abs122.1 121/LM18.1.17 | $IC_{50}$ (μg/mL) | $IC_{50}$ (nM) | % Neut | $R^2$ |
|---|---|---|---|---|
| WT | 0.007 | 0.047 | 95 | 0.95 |
| P.1 | 0.060 | 0.398 | 96 | 0.97 |
| B.1.351 | 0.117 | 0.781 | 94 | 0.94 |
| B.1.617 | 0.649 | 4.329 | 90 | 0.85 |
| B.1.617.2 | 0.201 | 1.340 | 98 | 0.97 |
| E484Q | 0.021 | 0.138 | 96 | 0.84 |
| L452R | 0.187 | 1.247 | 96 | 0.94 |

| Abs137.1 136/LM18.1.17 | $IC_{50}$ (μg/mL) | $IC_{50}$ (nM) | % Neut | $R^2$ |
|---|---|---|---|---|
| WT | 0.042 | 0.280 | 97 | 0.95 |
| P.1 | 0.038 | 0.250 | 98 | 0.95 |
| B.1.351 | 0.047 | 0.314 | 98 | 0.97 |
| B.1.617 | 0.027 | 0.183 | 94 | 0.97 |
| B.1.617.2 | 0.104 | 0.693 | 103 | 0.96 |
| E484Q | 0.107 | 0.710 | 100 | 0.95 |
| L452R | 0.029 | 0.194 | 101 | 0.98 |

| Abs199.1 198/LM18.1.17 | $IC_{50}$ (μg/mL) | $IC_{50}$ (nM) | % Neut | $R^2$ |
|---|---|---|---|---|
| WT | 0.009 | 0.060 | 101 | 0.99 |
| P.1 | 0.005 | 0.033 | 99 | 1.00 |
| B.1.351 | 0.008 | 0.053 | 101 | 0.98 |
| B.1.617 | 0.011 | 0.073 | 100 | 0.99 |
| B.1.617.2 | 0.129 | 0.863 | 101 | 1.00 |
| E484Q | 0.010 | 0.067 | 101 | 0.99 |
| L452R | 0.015 | 0.100 | 102 | 0.96 |

| Abs226.1 225/LM18.1.17 | $IC_{50}$ (μg/mL) | $IC_{50}$ (nM) | % Neut | $R^2$ |
|---|---|---|---|---|
| WT | 0.007 | 0.047 | 101 | 0.97 |
| P.1 | 0.001 | 0.007 | 94 | 0.90 |
| B.1.351 | 0.003 | 0.020 | 101 | 0.99 |
| B.1.617 | 0.004 | 0.027 | 100 | 0.99 |
| B.1.617.2 | 0.004 | 0.026 | 100 | 1.00 |
| E484Q | 0.060 | 0.400 | 101 | 1.00 |
| L452R | 0.008 | 0.053 | 101 | 1.00 |

| Abs238.1 237/LM18.1.17 | $IC_{50}$ (μg/mL) | $IC_{50}$ (nM) | % Neut | $R^2$ |
|---|---|---|---|---|
| WT | 0.006 | 0.040 | 102 | 0.96 |
| P.1 | 0.002 | 0.013 | 97 | 0.85 |
| B.1.351 | 0.003 | 0.020 | 101 | 0.93 |
| B.1.617 | 0.004 | 0.027 | 100 | 0.91 |
| B.1.617.2 | 0.004 | 0.028 | 101 | 1.00 |
| E484Q | 0.003 | 0.020 | 101 | 0.93 |
| L452R | 0.003 | 0.020 | 100 | 0.97 |

| Abs241.1 240/LM18.1.17 | $IC_{50}$ (μg/mL) | $IC_{50}$ (nM) | % Neut | $R^2$ |
|---|---|---|---|---|
| WT | 0.015 | 0.100 | 102 | 0.98 |
| P.1 | 0.002 | 0.013 | 97 | 0.98 |
| B.1.351 | 0.004 | 0.027 | 100 | 0.99 |
| B.1.617 | 0.007 | 0.047 | 100 | 1.00 |
| B.1.617.2 | 0.017 | 0.111 | 102 | 0.96 |
| E484Q | 0.010 | 0.067 | 99 | 0.99 |
| L452R | 0.010 | 0.067 | 99 | 0.98 |

| Abs256.1 255/LM18.1.17 | $IC_{50}$ (μg/mL) | $IC_{50}$ (nM) | % Neut | $R^2$ |
|---|---|---|---|---|
| WT | 0.004 | 0.027 | 101 | 0.93 |
| P.1 | 0.002 | 0.013 | 99 | 0.90 |
| B.1.351 | 0.001 | 0.007 | 101 | 0.93 |
| B.1.617 | 0.001 | 0.007 | 100 | 0.94 |
| B.1.617.2 | 0.002 | 0.013 | 100 | 0.97 |
| E484Q | 0.003 | 0.020 | 100 | 0.95 |
| L452R | 0.004 | 0.027 | 100 | 0.98 |

**Table S11.** X-ray crystallography data collection and processing statistics (26).

| | Nb225 + SARS-CoV-2 RBD + CC12.1 Fab | Nb240 + SARS-CoV-2 RBD + CC12.1 Fab | Nb255 + SARS-CoV-2 RBD + CC12.1 Fab |
|---|---|---|---|
| **Data collection** | | | |
| Beamline | SSRL 12-1 | SSRL 12-2 | APS 23-IDB |
| Wavelength (Å) | 0.97946 | 0.97946 | 1.03317 |
| Space group | $C\,2\,2\,2_1$ | $C\,2\,2\,2_1$ | $C\,2\,2\,2_1$ |
| Unit cell parameters | | | |
| a, b, c (Å) | 88.7, 143.1, 147.7 | 90.2, 114.7, 148.1 | 78.5, 140.4, 142.1 |
| α, β, γ (°) | 90, 90, 90 | 90, 90, 90 | 90, 90, 90 |
| Resolution (Å) [a] | 50.0–2.70 (2.75–2.70) | 50.0-2.83 (2.92-2.83) | 50.0 –2.20 (2.24–2.20) |
| Unique reflections [a] | 24,814 (1221) | 22,701 (1431) | 38,288 (1884) |
| Redundancy [a] | 4.1 (4.1) | 3.9 (3.5) | 6.5 (6.3) |
| Completeness (%) [a] | 96.9 (97.5) | 98.0 (96.4) | 96.4 (96.1) |
| $\langle I/\sigma_I \rangle$ [a] | 10.8 (1.3) | 6.3 (1.0) | 12.5 (1.3) |
| $R_{sym}$[b] (%) [a] | 11.7 (>100) | 18.4 (80.1) | 13.1 (>100) |
| $R_{pim}$[b] (%) [a] | 6.1 (64.3) | 10.3 (49.2) | 5.4 (43.0) |
| $CC_{1/2}$[c] (%) [a] | 99.2 (46.7) | 97.6 (49.2) | 99.2 (68.1) |
| **Refinement statistics** | | | |
| Resolution (Å) | 38.0-2.72 | 43.2–2.83 | 49.9-2.21 |
| Reflections (work) | 22,063 | 21,427 | 29,803 |
| Reflections (test) | 1215 | 1198 | 1150 |
| $R_{cryst}$[d] / $R_{free}$[e] (%) | 22.0/26.1 | 23.3/28.2 | 23.7/28.6 |
| No. of atoms | 5,655 | 5,728 | 5,831 |
| Macromolecules | 5,641 | 5,714 | 5,751 |
| Glycans | 14 | 14 | 14 |
| Solvent | - | - | 66 |
| Average $B$-value (Å$^2$) | 55 | 41 | 37 |
| Macromolecules | 55 | 41 | 37 |
| Nanobody | 85 | 74 | 37 |
| Fab | 49 | 35 | 39 |
| RBD | 49 | 33 | 35 |
| Glycans | 77 | 59 | 51 |
| Solvent | - | - | 33 |
| Wilson $B$-value (Å$^2$) | 36 | 35 | 30 |
| **RMSD from ideal geometry** | | | |
| Bond length (Å) | 0.002 | 0.002 | 0.002 |
| Bond angle (°) | 0.60 | 0.56 | 0.53 |
| **Ramachandran statistics (%)** | | | |
| Favored | 97.8 | 98.1 | 98.0 |
| Outliers | 0.0 | 0.0 | 0.0 |
| **PDB code** | 8ELO | 8ELP | 8ELQ |

[a] Numbers in parentheses refer to the highest resolution shell.
[b] $R_{sym} = \Sigma_{hkl} \Sigma_i | I_{hkl,i} - \langle I_{hkl} \rangle | / \Sigma_{hkl} \Sigma_i I_{hkl,i}$ and $R_{pim} = \Sigma_{hkl} (1/(n-1))^{1/2} \Sigma_i | I_{hkl,i} - \langle I_{hkl} \rangle | / \Sigma_{hkl} \Sigma_i I_{hkl,i}$, where $I_{hkl,i}$ is the scaled intensity of the i$^{th}$ measurement of reflection h, k, l, $\langle I_{hkl} \rangle$ is the average intensity for that reflection, and $n$ is the redundancy.
[c] $CC_{1/2}$ = Pearson correlation coefficient between two random half datasets.
[d] $R_{cryst} = \Sigma_{hkl} | F_o - F_c | / \Sigma_{hkl} | F_o | \times 100$, where $F_o$ and $F_c$ are the observed and calculated structure factors, respectively.
[e] $R_{free}$ was calculated as for $R_{cryst}$, but on a test set comprising 5% of the data excluded from refinement.
[f] From MolProbity.

**Table S12.** Cryo-EM data collection, processing, model refinement and validation statistics.

| **Map** | **LM18/Nb136 bsNb$_4$-Ig + CoV-2 6P-Mut7 S (focused refinement)** | **LM18/Nb136 bsNb$_4$-Ig + CoV-2 6P-Mut7 S (global refinement)** |
|---|---|---|
| EMDB | EMD-27692 | EMD-27693 |
| **Data collection** | | |
| Microscope | Thermo Fisher Titan Krios | Thermo Fisher Titan Krios |
| Voltage (kV) | 300 | 300 |
| Detector | Gatan K2 Summit | Gatan K2 Summit |
| Recording mode | Counting | Counting |
| Nominal magnification | 130,000x | 130,000x |
| Movie micrograph pixelsize (Å) | 1.045 | 1.045 |
| Dose rate ($e^-$/[(camera pixel)*s]) | 6.02 | 6.02 |
| Number of frames per movie micrograph | 36 | 36 |
| Frame exposure time (ms) | 250 | 250 |
| Movie micrograph exposure time (s) | 9.0 | 9.0 |
| Total dose ($e^-$/Å$^2$) | 49.6 | 49.6 |
| Defocus range (μm) | -0.5 to -1.8 | -0.5 to -1.8 |
| **EM data processing** | | |
| Number of movie micrographs | 4,296 | 4,296 |
| Number of molecular projection images in map | 50,160 | 61,539 |
| Symmetry | C1 | C1 |
| Map resolution (FSC 0.143; Å) | 3.3 | 3.1 |
| Map sharpening B-factor (Å$^2$) | -52.2 | -67.4 |
| **Structure building and validation** | | |
| *Number of atoms in deposited model* | | |
| CoV-2 6Pmut7 S | 20,691 | *n/a* |
| LM18/Nb136 bsNb$_4$-Ig | 1,923 | *n/a* |
| glycans | 182 | *n/a* |
| MolProbity score | 0.98 | *n/a* |
| Clashscore | 1.13 | *n/a* |
| Map correlation coefficient | 0.82 | *n/a* |
| EMRinger score | 2.81 | *n/a* |
| d FSC model (0.5; Å) | 3.4 | *n/a* |
| *RMSD from ideal* | | |
| Bond length (Å) | 0.005 | *n/a* |
| Bond angles (°) | 0.967 | *n/a* |
| *Ramachandran plot* | | |
| Favored (%) | 97.15 | *n/a* |
| Allowed (%) | 2.85 | *n/a* |
| Outliers (%) | 0.00 | *n/a* |
| Side chain rotamer outliers (%) | 0.20 | *n/a* |
| Cβ outliers (%) | 0.00 | *n/a* |
| PDB | 8dt8 | *n/a* |

**Table S13.** Amino acid frequencies used to generate the trimer phosphoramidite mixtures for the construction of the naïve library.

| Codon | AA | [TriMix1] | [TriMix2] | [TriMix3] | [TriMix4] | [TriMix5] |
|---|---|---|---|---|---|---|
| AAA (Lys) | K | 0.5 | 0.6 | 1 | 0.6 | 0.9 |
| AAC (Asn) | N | 1.5 | 1.9 | 1.5 | 1.6 | 1.8 |
| ACT (Thr) | T | 5.1 | 5.4 | 6.2 | 4.6 | 5 |
| ATC (Ile) | I | 2.5 | 2.5 | 3 | 2.7 | 3.4 |
| ATG (Met) | M | 0 | 0 | 0 | 0 | 0 |
| CAG (Gln) | Q | 3 | 2.4 | 2.1 | 2.2 | 2.6 |
| CAT (His) | H | 2.5 | 1.7 | 1.4 | 1.8 | 2 |
| CCG (Pro) | P | 4.7 | 5.8 | 5.7 | 6.5 | 7.6 |
| CGT (Arg) | R | 6.3 | 7.2 | 5 | 8 | 9.4 |
| CTG (Leu) | L | 4.8 | 4 | 3.5 | 4.3 | 5.3 |
| GAA (Glu) | E | 4.3 | 2.7 | 1.6 | 2.2 | 2.4 |
| GAC (Asp) | D | 6.4 | 9 | 2.8 | 6.2 | 6.3 |
| GCT (Ala) | A | 6.7 | 6 | 7.5 | 5.2 | 5.8 |
| GGT (Gly) | G | 18.1 | 15.3 | 12 | 13.4 | 12.2 |
| GTT (Val) | V | 5.4 | 5.2 | 9.9 | 5.1 | 6.4 |
| TAC (Tyr) | Y | 8.9 | 13.8 | 22.2 | 17.6 | 13.5 |
| TCT (Ser) | S | 11.6 | 8.5 | 7.4 | 7.8 | 7.4 |
| TGC (Cys) | C | 0 | 0 | 0 | 0 | 0 |
| TGG (Trp) | W | 4.5 | 5 | 3.6 | 6.9 | 5 |
| TTC (Phe) | F | 3.1 | 2.9 | 3.6 | 3.3 | 2.9 |

**Table S14.** Primer sequences used in this study.

| Name | 5'-3' sequence |
|---|---|
| CDRH3_L10 | AGCTGTCTACTACTGCGCCAGG[TriMix1][TriMix5][TriMix5][TriMix1][TriMix2]TTCGACTACTGGGGACAAGGTACGTTGGTC |
| CDRH3_L11 | AGCTGTCTACTACTGCGCCAGG[TriMix1][TriMix5][TriMix2][TriMix2][TriMix2][TriMix5]TTCGACTACTGGGGACAAGGTACGTTGGTC |
| CDRH3_L12 | AGCTGTCTACTACTGCGCCAGG[TriMix1][TriMix5][TriMix5][TriMix4][TriMix5][TriMix2][TriMix5]TTCGACTACTGGGGACAAGGTACGTTGGTC |
| CDRH3_L13 | AGCTGTCTACTACTGCGCCAGG[TriMix1][TriMix5][TriMix5][TriMix2][TriMix2][TriMix5][TriMix2][TriMix5]TTCGACTACTGGGGACAAGGTAC TTGGTC |
| CDRH3_L14 | AGCTGTCTACTACTGCGCCAGG[TriMix1][TriMix5][TriMix5][TriMix4][TriMix4][TriMix4][TriMix2][TriMix2][TriMix3]TTCGACTACTGGGGACAAGGTACGTTGGTC |
| CDRH3_L15 | AGCTGTCTACTACTGCGCCAGG[TriMix1][TriMix5][TriMix5][TriMix4][TriMix4][TriMix4][TriMix4][TriMix4][TriMix1][TriMix3]TTCGACTACTGGGGACAAGGTACGTTGGTC |
| CDRH3_L16 | AGCTGTCTACTACTGCGCCAGG[TriMix1][TriMix2][TriMix5][TriMix5][TriMix4][TriMix4][TriMix4][TriMix4][TriMix2][TriMix1][TriMix3]TTCGACTACTGGGGACAAGGTACGTTGGTC |
| CDRH3_L17 | AGCTGTCTACTACTGCGCCAGG[TriMix1][TriMix2][TriMix5][TriMix5][TriMix4][TriMix4][TriMix4][TriMix4][TriMix4][TriMix5][TriMix1][TriMix3]TTCGACTACTGGGGACAAGGTACGTTGGTC |
| CDRH3_L18 | AGCTGTCTACTACTGCGCCAGG[TriMix1][TriMix2][TriMix5][TriMix5][TriMix4][TriMix5][TriMix2][TriMix4][TriMix4][TriMix4][TriMix4][TriMix2][TriMix3]TTCGACTACTGGGGACAAGGTACGTTGGTC |
| CDRH3_L19 | AGCTGTCTACTACTGCGCCAGG[TriMix1][TriMix5][TriMix5][TriMix5][TriMix4][TriMix2][TriMix2][TriMix2][TriMix4][TriMix5][TriMix4][TriMix4][TriMix2][TriMix3]TTCGACTACTGGGGACAAGGTACGTTGGTC |
| CDRH3_L20 | AGCTGTCTACTACTGCGCCAGG[TriMix1][TriMix2][TriMix4][TriMix4][TriMix4][TriMix2][TriMix5][TriMix2][TriMix2][TriMix4][TriMix5][TriMix4][TriMix2][TriMix2][TriMix3]TTCGACTACTGGGGACAAGGTACGTTGGTC |
| NbSS_AmpF1 | CGGTTTGTCATCTACAAATACAACAATCGCATCC |
| NB_Ramp1 | CTAGGAGTTCAGGTGCTGGTGATGGAG |
| hNb323_NGSSeq_Fa | GTCTCGTGGGCTCGGAGATGTGTATAAGAGACAGCTGATTCTGTTAAAGGTAGATTTACTTGTTCTAGAG |
| hNb323_NGSSeq_Fb | GTCTCGTGGGCTCGGAGATGTGTATAAGAGACAGHHCTGATTCTGTTAAAGGTAGATTTACTTGTTCTAGAG |
| hNb323_NGSSeq_Fc | GTCTCGTGGGCTCGGAGATGTGTATAAGAGACAGHHHHCTGATTCTGTTAAAGGTAGATTTACTTGTTCTAGAG |
| hNb323_NGSSeq_Ra | TCGTCGGCAGCGTCAGATGTGTATAAGAGACAGCAGTGACCTGCGTACCTTGTCC |
| hNb323_NGSSeq_Rb | TCGTCGGCAGCGTCAGATGTGTATAAGAGACAGHHCAGTGACCTGCGTACCTTGTCC |
| hNb323_NGSSeq_Rc | TCGTCGGCAGCGTCAGATGTGTATAAGAGACAGHHHHCAGTGACCTGCGTACCTTGTCC |

**SI References**


1. F. Zhao, *et al.*, Broadening a SARS-CoV-1 neutralizing antibody for potent SARS-CoV-2 neutralization through directed evolution *bioRxiv* [preprint] doi.org/10.1101/2021.05.29.443900 (2021). Accessed February 9 2022.

2. S. A. Sievers, L. Scharf, A. P. West Jr, P. J. Bjorkman, Antibody engineering for increased potency, breadth and half-life. *Curr. Opin. HIV AIDS* **10**, 151–159 (2015).

3. Y. Xu, *et al.*, Addressing polyspecificity of antibodies selected from an in vitro yeast presentation system: a FACS-based, high-throughput selection and analytical tool. *Protein Eng. Des. Sel.* **26**, 663–670 (2013).

4. A. Simons, *A quality control tool for high throughput sequence data*. https://www.bioinformatics.babraham.ac.uk/projects/fastqc/

5. B. Bushnell, J. Rood, E. Singer, BBMerge – Accurate paired shotgun read merging via overlap. *PLoS One* **12**, e0185056 (2017).

6. T. Rognes, T. Flouri, B. Nichols, C. Quince, F. Mahé, VSEARCH: a versatile open-source tool for metagenomics. *PeerJ* **4**, e2584 (2016).

7. T. F. Rogers, *et al.*, Isolation of potent SARS-CoV-2 neutralizing antibodies and protection from disease in a small animal model. *Science* **369**, 956–963 (2020).

8. Z. Otwinowski, W. Minor, Processing of X-ray diffraction data collected in oscillation mode. *Methods Enzymol.* **276**, 307–326 (1997).

9. A. J. McCoy, *et al.*, Phaser crystallographic software. *J. Appl. Crystallogr.* **40**, 658–674 (2007).

10. P.-A. Koenig, *et al.*, Structure-guided multivalent nanobodies block SARS-CoV-2 infection and suppress mutational escape. *Science* **371**, eabe6230 (2021).

11. M. Yuan, *et al.*, Structural basis of a shared antibody response to SARS-CoV-2. *Science* **369**, 1119–1123 (2020).

12. P. Emsley, K. Cowtan, Coot: model-building tools for molecular graphics. *Acta Crystallogr. D Biol. Crystallogr.* **60**, 2126–2132 (2004).

13. P. D. Adams, *et al.*, PHENIX: a comprehensive Python-based system for macromolecular structure solution. *Acta Crystallogr. D Biol. Crystallogr.* **66**, 213–221 (2010).

14. A. Cheng, *et al.*, Leginon: New features and applications. *Protein Sci.* **30**, 136–150 (2021).

15. S. Q. Zheng, *et al.*, MotionCor2: anisotropic correction of beam-induced motion for improved cryo-electron microscopy. *Nat. Methods* **14**, 331–332 (2017).

16. A. Punjani, J. L. Rubinstein, D. J. Fleet, M. A. Brubaker, cryoSPARC: algorithms for rapid unsupervised cryo-EM structure determination. *Nat. Methods* **14**, 290–296 (2017).

17. K. Zhang, Gctf: Real-time CTF determination and correction. *J. Struct. Biol.* **193**, 1–12 (2016).

18. A. Punjani, H. Zhang, D. J. Fleet, Non-uniform refinement: adaptive regularization improves single-particle cryo-EM reconstruction. *Nat. Methods* **17**, 1214–1221 (2020).

19. J. Zivanov, T. Nakane, S. H. W. Scheres, Estimation of high-order aberrations and anisotropic magnification from cryo-EM data sets in -3.1. *IUCrJ* **7**, 253–267 (2020).

20. E. F. Pettersen, *et al.*, UCSF Chimera a visualization system for exploratory research and analysis. *J. Comput. Chem.* **25**, 1605–1612 (2004).

21. J. Dunbar, *et al.*, SAbPred: a structure-based antibody prediction server. *Nucleic Acids Res.* **44**, W474–8 (2016).

22. A. Casañal, B. Lohkamp, P. Emsley, Current developments in Coot for macromolecular model building of Electron Cryo-microscopy and Crystallographic Data. *Protein Sci.* **29**, 1069–1078 (2020).

23. P. V. Afonine, *et al.*, Real-space refinement in PHENIX for cryo-EM and crystallography. *Acta Crystallogr D Struct Biol* **74**, 531–544 (2018).

24. B. A. Barad, *et al.*, EMRinger: side chain-directed model and map validation for 3D cryo-electron microscopy. *Nat. Methods* **12**, 943–946 (2015).

25. C. J. Williams, *et al.*, MolProbity: More and better reference data for improved all-atom structure validation. *Protein Sci.* **27**, 293–315 (2018).

26. P.-S. Huang, *et al.*, RosettaRemodel: a generalized framework for flexible backbone protein design. *PLoS One* **6**, e24109 (2011).

27. F. Zhao, *et al.*, Engineering SARS-CoV-2 neutralizing antibodies for increased potency and reduced viral escape pathways. *iScience* **25**, (9) (2022)
